# Phytochemical characterization and comparative studies of four *Cecropia* species collected in Panama using multivariate data analysis

**DOI:** 10.1038/s41598-018-38334-4

**Published:** 2019-02-11

**Authors:** Andrés Rivera-Mondragón, Sebastiaan Bijttebier, Emmy Tuenter, Deborah Custers, Orlando O. Ortíz, Luc Pieters, Catherina Caballero-George, Sandra Apers, Kenn Foubert

**Affiliations:** 1 Natural Products & Food Research and Analysis (NatuRA), Department of Pharmaceutical Sciences, University of Antwerp, Universiteitsplein 1, 2610 Antwerp, Belgium; 2 Flemish Institute for Technological Research (VITO), Business Unit Separation and Conversion Technology (SCT), Mol, Belgium; 3 Herbario PMA, Universidad de Panamá, Estafeta Universitaria, Panama City, Republic of Panama; 4 Centre of Innovation and Technology Transfer, Institute of Scientific Research and High Technology Services (INDICASAT-AIP), Building 208, City of Knowledge, Panama, Republic of Panama

## Abstract

Plant species of the genus *Cecropia* (Urticaceae) are used as traditional medicine in Latin-America, and are commercially available as food supplements. The aim of this study was to characterize and compare the phytochemical constituents of four *Cecropia* species collected in Panama. The structures of 11 compounds isolated from leaves of *C. obtusifolia* were elucidated based on high resolution mass spectrometry (HRMS) and nuclear magnetic resonance (NMR) spectroscopic analysis; the polyphenolic constituents of leaves of all four *Cecropia* species and commercial products were characterized using high performance liquid chromatography-diode array detection-quadrupole time of flight-tandem high resolution mass spectrometry (HPLC-DAD-QTOF). Forty-seven compounds were fully identified or tentatively characterized. Thirty-nine of these have not been previously reported for the species under investigation. Multivariate analysis revelead that *C. obtusifolia* and *C. insignis* are the most related species, while *C. hispidissima* is the most segregated one. Considering the importance of the description of novel chemical entities and the increasing interest and use of natural products, this study may be of great help for chemotaxonomic purposes, the interpretation of medicinal properties and for quality assessment of herbal supplements containing *Cecropia* leaves.

## Introduction

The use of medicinal plants has been evidenced since ancient times for the treatment of a wide range of illnesses, and they have become increasingly important in healthcare. Although the use of phytomedicines was based on empiric experience in the past, nowadays is increasingly based on scientific evidence regarding their chemical composition and associated medicinal properties^1^.

It is well known that many medicinal plants with similar morphology and the same folk name among species within the same genus can be misidentified and intentionally or accidentally be substituted in commercial products during the manufacturing process, and may result in the loss of efficacy and safety. The authentication of biological species is usually performed by experienced taxonomists, however, morphological identification can be limited by the absence of different phenotypic characteristics. Furthermore, herbal products are available in the marked as their processed forms, such as herbs (i.e. leaf powder and crushed material), herbal material (i.e. essential oils), herbal preparations (i.e. extracts) and finished herbal products (i.e. capsules and tablets), and consequently morphological identification is quite challenging^2,3^. Therefore, phytochemical studies may provide a useful tool for the authentification and discrimination between similar plant species.

The genus *Cecropia* Loefl. (Urticaceae) is a widespread and abundant fast-growing dioecious tree distributed across the tropical and subtropical rainforest from Mexico to Central and South America below 2600 m above sea level. It is comprised of 61 species^4^. Most of the species are known as ‘yarumo’, ‘guarumo’, ‘guarumbo’, ‘embauba’, ‘ambay’, ‘trumped tree’, among other common names, and are ant- plants or myrmecophytes, that is, they live in a mutualistic relationship with a colony of symbiotic ants (especially associated to the genus *Azteca*)^5^.

Some species of this genus are extensively used in several countries of Latin America for the treatment of a variety of life threatening diseases. A literature revision has previously been published by Costa *et al*.^6^ and Rivera-Mondragón *et al*.^5^ regarding a considerable amount of scientific reports on the ethnomedicinal use, chemical and pharmacological features of the genus *Cecropia*. It has been documented that a water infusion is conventionally prepared (mainly from the leaves of the plant) and drunk throughtout the day for the control of diabetes, hypertension, respiratory diseases and inflammation. Furthermore, wound healing and antimalarial properties have been reported.

Latin American folk medicine emphasizes on the widespread use of *C. obtusifolia* and *C. peltata* for the treatment of diabetes mellitus and hypertention^7–13^. These two species are reported to be distributed in México, Central America, the Caribbean region, Colombia and Ecuador^14^. On the other hand, *C. glaziovii*, *C. pachystachya* and *C. hololeuca* (distributed in South America, mainly in Brazil and Argentina) are frequently used for the control of inflammation, hypertension and respiratory conditions^15–21^.

These species have been largely studied through several phytochemical and pharmacological investigations reported in literature, where most of them have focused on polar compounds from leaf extracts. The range of therapeutic properties attributed to these plants has been correlated to their content of phenolic acids, flavonoids and triterpenoids. Chlorogenic acid and flavone *C*-glycosides (such as orientin, isoorientin, vitexin, isovitexin) have been consistently reported as the main compounds in *C. obtusifolia*, *C. peltata*, *C. glaziovii*, *C. pachystachya* and *C. hololeuca*^7,8,15,19,21–26^. Additionally, *O*-glycosyl flavonols (quercetin, rutin and isoquercitrin), flavan-3-ols (catechin and epicatechin) and condensed tannins (procyanidin B2, B3, B5 and C1) have been described in *C. glaziovii*, *C. hololeuca* and *C. pachystachya*^15,25–30^. On the other hand, ß-sitosterol, ursolic, tormentic, euscaphic, isoarjulonic and pomolic acid from root and leaf extracts from *C. pachystachya* have also been reported^31–33^.

Few analytical methods have been published concerning the determination of the main phenolic compounds in *Cecropia* species. Detection has mainly been performed by high-performance liquid chromatography coupled to photodiode-array detection (HPLC-DAD) to determine isoorientin and isovitexin in aqueous and butanolic extracts of *C. obtusifolia* and *C. peltata*^7,34^; to examine the seasonal variation of chlorogenic acid, caffeic acid, isoorientin, isovitexin, total flavonoids and proanthocyanidins in *C. glaziovii*^35–37^; and for the fingerprinting and quantification of methanolic and ethyl acetate extracts of *C. pachystachya*^21,23,28^. Furthermore, only one validated HPLC-DAD method for the quantification of chlorogenic acid, isoorientin, orientin, isovitexin and isoquercitrin from leaves of *C. glaziovii* and *C. pachystachya* has been reported^38^.

In contrast, *C. insignis* and *C. hispidissima* have scarcely been described in high quality scientific literature regarding their phytochemical composition and related pharmacological properties, maybe due to their restricted geographical distribution^14^. In spite of this, the traditional use of *C.insignis* as diuretic, for the treatment of hypertension, asthma, bronquitis and inflammation has been reported^39,40^. On the other hand, the use of *C. hispidissima* is not clearly documented in folk medicine.

Due of their extensive use in several countries of Latin America, there is a growing interest in the study of medicinal plants of the *Cecropia* genus. For instance, the inclusion of some *Cecropia* species in national pharmacopoeias such as *Cecropia hololeuca*. (Brazilian Pharmacopeia, 1929), *Cecropia pachystachya* (Argentinian Pharmacopeia, 1978) and *Cecropia obtusifolia* (Herbal Pharmacopeia of the United Mexican States, 2001) shows the relevance of their medicinal use and official recognition in these countries^5,6^. In addition, the development of new delivery system of herbal nanoparticle formulations containing enriched leaf extracts from *C. obtusifolia*^41^ and *C. glaziovii*^42,43^ has also been reported.

The coexistence of different *Cecropia* species with the same folk name in the same territory (i.e. Mexico and Central America) may lead to misidentification and potentially inappropriate use. However, little is known about whether substitution of one plant species for another one has serious implicatios or any significant negative impact on human health (efficacy/safety). In this respect, the aim of this study was to characterize and compare the phytochemical composition in methanolic extracts of four species belonging to the *Cecropia* genus. Although two LC-HRMS chemical profiling studies have been conducted on *C. pachystachya* and *C. holoceuca*^44,45^ collected in Brazil; in the present work we report for the first time a comprehensive chemical profiling method using HPLC-DAD-QTOF and a multivariate analytical aproach to reveal differences and similarities in the phytochemical composition of *C. obtusifolia*, *C. peltata*, *C. insignis* and *C. hispidissima* collected in Panama. The current study attempts to provide a global overview of the chemical composition of these four species, thus giving a better insight in its application for chemotaxonomic studies, understanding of their medicinal properties and further studies aiming for the quality control of herbal supplements containing *Cecropia* leaves.

## Results and Discussion

### Isolation and identification of compounds from *Cecropia obtusifolia*

**Figure 1:**
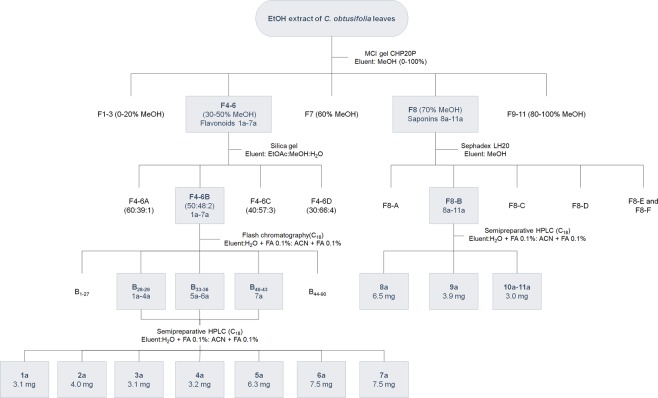
Isolation scheme of the 70% EtOH extract from the leaves of *Cecropia obtusifolia* (O1).

**Figure 2:**
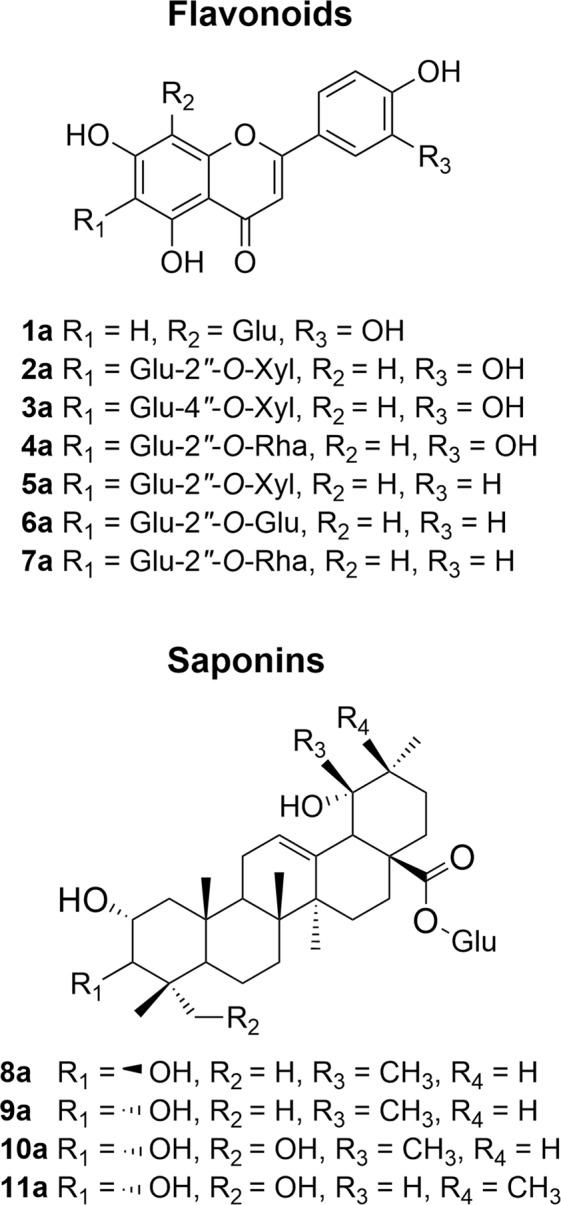
Chemical structure of compounds **1a**-**11a** isolated from *Cecropia obtusifolia* (O1).

Eleven (**1a**-**11a**) compounds were isolated from a 70% EtOH extract from the leaves of *C. obtusifolia* (O1) by successive chromatographic separations (MCI gel, silica gel, sephadex LH-20 and automated flash chromatography) and semi-preparative HPLC (Figs 1 and 2).

Seven known flavone glycosides (**1a**-**7a**) and four triterpenoid saponins (**8a**-**11a**) were identified as orientin (**1a**)^46^, isoorientin-2″-*O*-xyloside (**2a**)^47^, isoorientin-4″-O-xyloside (3a)^48^, isoorientin-2″-*O*-rhamnoside (**4a**)^48,49^, isovitexin-2″-*O*-xyloside (**5a**)^47,50^, isovitexin-2″-*O*-glucoside (**6a**), isovitexin-2″-*O*-rhamnoside (**7**)^49^, tormentic acid 28-*O*-glucoside (tormentoside) (**8a**)^51,52^, euscaphic acid 28-*O*-glucoside (**9a**) (Kaji-ichigoside F1)^52^, niga-ichigoside F2 (**10a**)^52,53^ and buergericic acid 28-*O*-glucoside (**11a**)^53^ by direct comparison of their spectroscopy data (^1^H and ^13^C NMR, and MS) with those previously reported in the literature. All assignments and spectra are available as Supplementary Information (Tables S1–S5, Figs S1–S46). Isoorientin and isovitexin *O*-glycosides (**2a**-**7a**) and saponins (**10a**-**11a**) were previously found to be undescribed for *Cecropia obtusifolia*.

### Phytochemical analysis of species of the genus *Cecropia*

The polyphenolic content of four authentic plant species and commercial products of the genus *Cecropia* species (15 samples) were analized. The plant materials were collected in October 2015 and July 2016. According to Electricity Transmission Company (ETESA)^54^, October was characterized by heavy rainfalls and thunderstorms. In contrast, July showed a decrease in rainfalls, giving rise to a second dry period during the rainy season (known as First Canicula).

**Figure 3:**
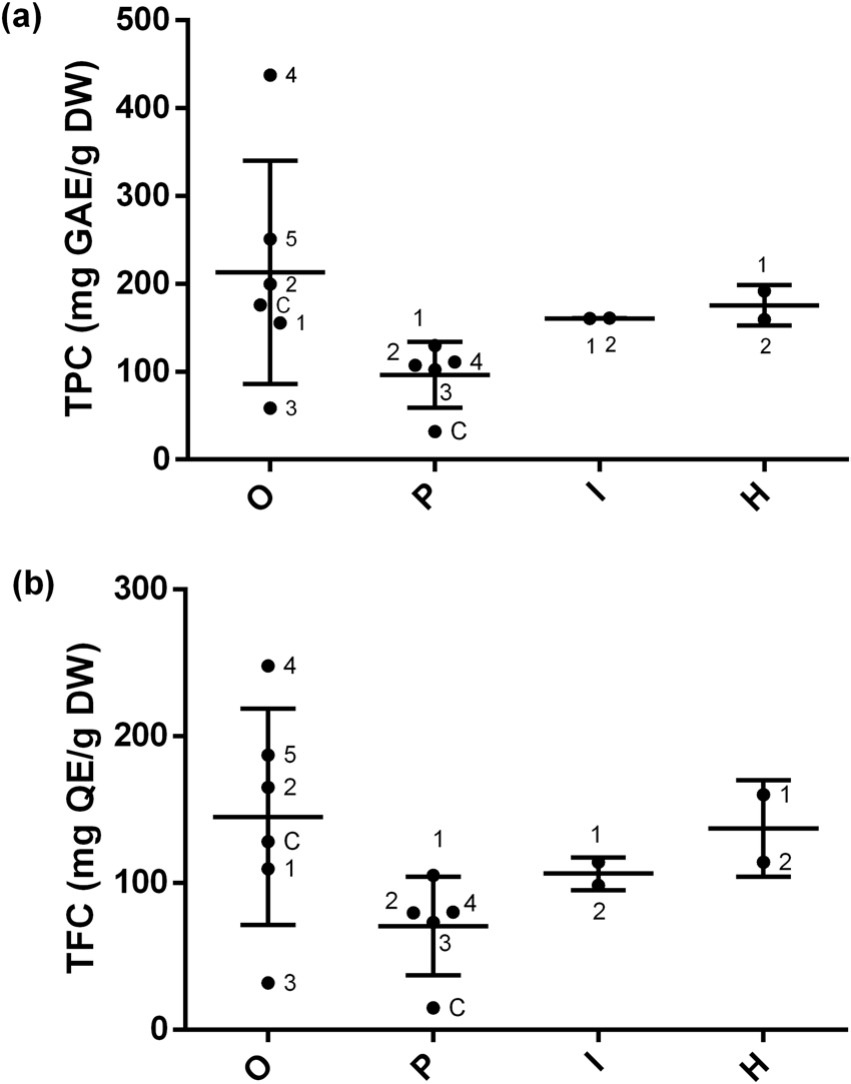
Variability of TPC (**a**) and TFC (**b**) between the four *Cecropia* species. O: *C. obtusifolia*; P: *C. peltata*; I: *C. insignis*; and H, *C. hispidissima*. TPC: total phenol content; mGAE/g DW, GAE: gallic acid equivalents. TFC: total flavonoid content; mg QE/g DW: quercetin equivalents, gram of dried weight (% w/w). The error bar was calculated from SD. ANOVA followed by multiple comparison test (Tukey method) showed no significant differences between the mean of each group.

As a first step, extracts were assessed by the determination of their total phenolic (TPC) and flavonoid (TFC) content (Fig. 3 and Table S6). In general, TPC and TFC showed a similar pattern in each individual leaf extract. Although no significant difference between the mean of each group was found, *C. obtusifolia* was shown to have the highest TPC and TFC compared to *C. hisspidissima*, *C. insignis* and *C. peltata* (in this order). The extract of *C. obtusifolia* (O4) contained the highest level of total phenolics and flavonoids (437.9 mg/GAE g DW and 247.9 mg/EQ g DW, respectively). Among the four analyzed species the commercial product from *C. peltata* (PC) presented the lowest amounts in terms of TPC and TFC (31.7 mg/GAE g DW and 14.9 mg/EQ g DW, respectively).

**Figure 4:**
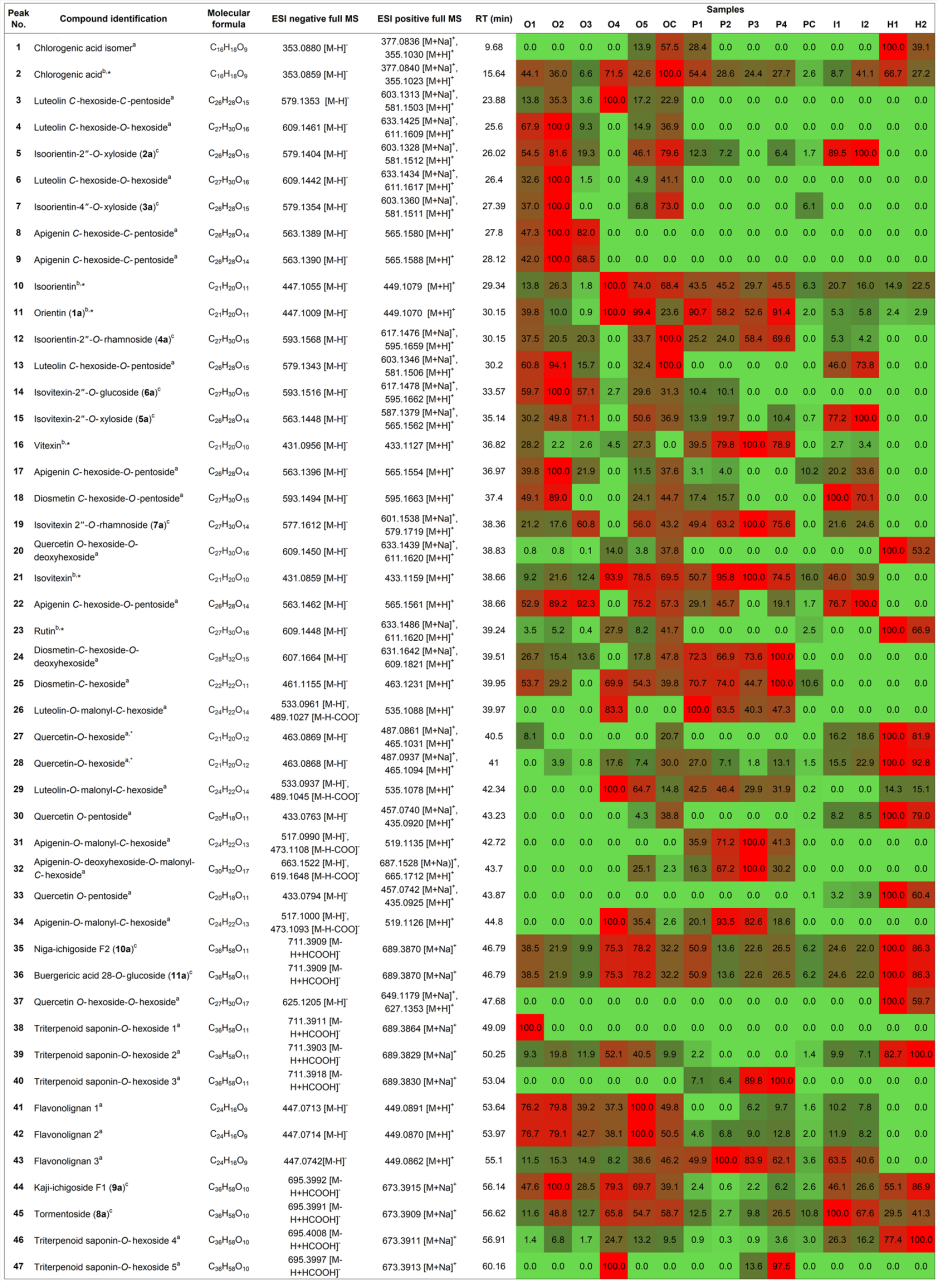
Average relative abundances (peak area/mg DW, %) of the identified compounds from *Cecropia* species leaves, analyzed by HPLC-DAD-QTOF. ^a^Tentative identification based on accurate mass. ^b^Identification with an analytical standard. ^c^Identification by comparison with pure compounds isolated from *C. obtusifolia* (identification number as described in *Isolation and identification of compounds from Cecropia obtusifolia*).*Previously reported in *C. obtusifolia*, *C. peltata*, *C. insignis* and/or *C.hisspidisima*. Light green = 0% and red = 100% relative abundance.

**Figure 5:**
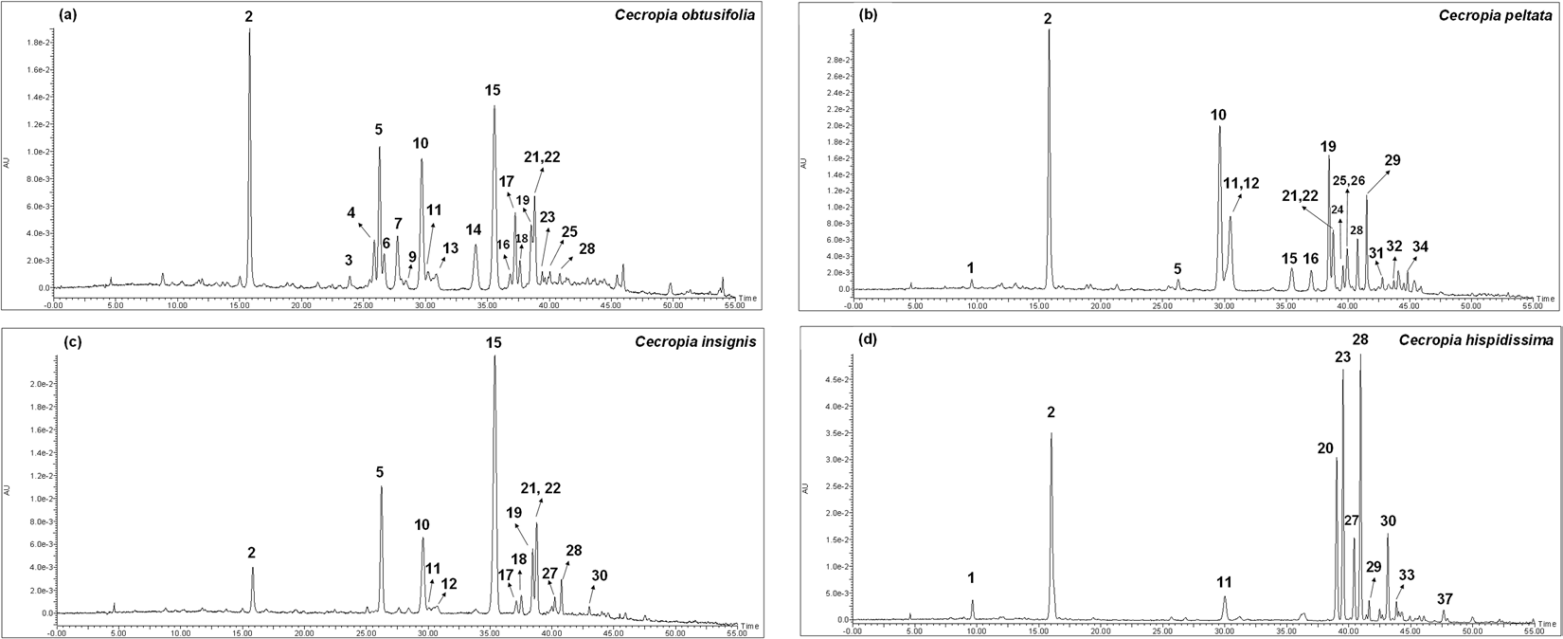
HPLC-UV chromatograms recorded at 340 nm of C*. obtusifolia* O2 (**a**), *C. peltata* P2 (**b**), *C. insignis* I1 (**c**) and *C. hispidissima* H1 (**d**).

The phytochemical profile of methanolic leaf extract from *Cecropia* species was further explored using an HPLC method coupled to both DAD and MS detectors. HPLC-DAD-QTOF analysis, in negative and in positive mode, allowed the identification or tentative characterization of a total of 47 phenolic compounds, including 2 phenolic acids, 33 flavonoids, 3 flavonolignans and 9 saponins, many of which were found to be unreported in *C. obtusifolia*, *C. peltata*, *C. insignis* and *C. hispidissima* (see Fig. 4). Compound identification was established by comparison of their retention time, ultraviolet (UV) absorption and electrospray ionization (ESI) mass spectra (based on main molecular ions and observed fragmentations) with analytical standards (**2**, **10**, **11**, **16**, **21** and **23**) or pure compounds isolated from *C. obtusifolia* (**5**, **7**, **12**, **14**, **15** and **19**). The remaining compounds were tentatively assigned based on the exact masses and fragmentation patterns. See Supplementary Table S7 for detailed information. Representative HPLC-DAD chromatograms are shown in Fig. 5.

#### Phenolic acids

Two phenolic acids (**1**, **2**) were observed in *Cecropia* species. Compound **2** was identified as chlorogenic acid by comparison with an authentic standard, while compound **1** was assigned as a chlorogenic acid isomer according to its [M − H]^−^ and [M + H]^+^ ions at *m/z* 353.0880 and 355.1030, respectively (Fig. 4). Moreover, compound **1** both in negative and positive modes showed characteristic product ions similar to authentic chlorogenic acid. It was observed a peak at *m/z* 191.1, corresponding to quinic acid product ion [M-H-162]^−^ and a subsequent product ion derived from quinic acid at *m/z* 173.1 [M-H-162-H_2_O]^−^. Similarly, typical caffeic acid-based products ions were observed at *m/z* 179.0 [M-H-174]^−^, 161.0 [M-H-174-H_2_O]^−^/163.0 [M + H-174-H_2_O]^+^, 145.0 [M + H-174-2H_2_O]^+^, 135.0 [M + H-174-H_2_O-CO]^+^, 117.0 [M + H-174-2H_2_O-CO]^+^. See Supplementary Table S7.

#### Flavonoids

Thirty three flavonoids (**3**–**34**, **37**) were detected in *Cecropia* species. UV spectra of most of the chromatographic peaks were characteristic of flavone and flavonol glycosides (see Supplementary Table S7). Typical UV-absorption bands with maxima in the 240–285 and 300–354 nm range were observed^55^. Flavonoids were detected as glycoconjugates, such as hexose (**10**, **11**, **16**, **21**, **25**, **27** and **28**), pentose (**30** and **33**), dihexose (**4**, **6**, **14** and **37**), hexose-deoxyhexose (**12**, **19**, **20**, **23** and **24**), hexose-pentose (**3**, **5**, **7**, **8**, **9**, **13**, **15**, **17**, **18** and **22**), malonyl-hexose (**26**, **29**, **31** and **34**), malonyl-hexose-deoxyhexose (**32**) (Fig. 4). Characteristic mass losses of *C*- and *O*-glycosides in hexoses, deoxyhexoses and pentoses (– ^0,1^X, – ^0,2^X, – ^0,3^X, – ^1,5^X, – ^2,3^X-2H_2_O, – ^0,4^X-2H_2_O, – ^0,2^X-H_2_O, – ^0,2^X-2H_2_O, – ^2,3^X-3H_2_O, – Y_i_, – Z_i_) were observed in MS^E^ spectra (MS fragmentation data)^56–58^ (see Supplementary Table S7). The main flavonoid aglycones of detected glycosides were suggestive of luteolin (**3**, **4**, **5**, **6**, **7**, **10**, **11**, **12**, **13**, **26** and **29**), apigenin (**8**, **9**, **14**, **15**, **16**, **17**, **19**, **21**, **22**, **31**, **32** and **34**), diosmetin (luteolin 4′-methyl ether: **18**, **24** and **25**) and quercetin (**20**, **23**, **27**, **28**, **30**, **33** and **37**), similar to has been described for *C. pachystachya* and *C. hololeuca*^44,45^. For example, compounds **18**, **24** and **25** revealed [M − H]^−^ ions at *m/z* 593.1494, 607.1664 and 461.1155; and [M + H]^+^ ions at *m/z* 595.1663, 609.1821 and 463.1231, respectively. MS^E^ product ion spectra showed characteristic losses of 96, 120 and 150 u suggesting that they are *C*-hexosides. Furthermore, compounds **18** and **24** produced [M + H-132]^+^ and [M + H-146]^+^ ions at *m/z* 463.1 corresponding to the loss of *O*-pentoside and *O*-deoxyhexoside moieties, respectively. Compounds **18**, **24** and **25** were tentatively assigned as diosmetin *C*-hexoside-*O*-pentoside, diosmetin *C*-hexoside-*O*-deoxyhexoside and diosmetin-*C*-hexoside. This is the first report of compounds **18**, **24** and **25** in *C. obtusifolia*, *C. peltata* and *C. insignis*. The latter (**25**) has been previously reported in *C. pachystachya*^45^.

Compounds **26** and **29** were tentatively assigned as luteolin-*O*-malonyl-*C*-hexoside ([M − H]^−^ at *m/z* 533.09 and [M + H]^+^ at *m/z* 535.10); **31** and **34** as apigenin-*O*-malonyl-*C*-hexoside ([M − H]^−^ at m/z 517.09 and [M + H]^+^ at *m/z* 519.11); and **32** as apigenin-*O*-deoxyhexoside-*O*-malonyl-*C*-hexoside ([M − H]^−^ at *m/z* 663.1522, [M + H]^+^ at *m/z* 665.1722, and [M + Na]^+^ at *m/z* 687.1528). The MS fragmentation of their parent ions [M − H]^−^ and [M + H]^+^ yielded product ions characteristic of losses of a malonyl residue [44 (CO_2_) and 86 (C_3_H_2_O_3_) Da]^59,60^, C-hexosyl (66, 96, 120 and 150 Da) and *O*-deoxyhexosyl (146 Da). These acetylated (malonyl) flavonoids have not previously reported in any species of the genus *Cecropia*.

#### Flavonolignans

**Figure 6:**
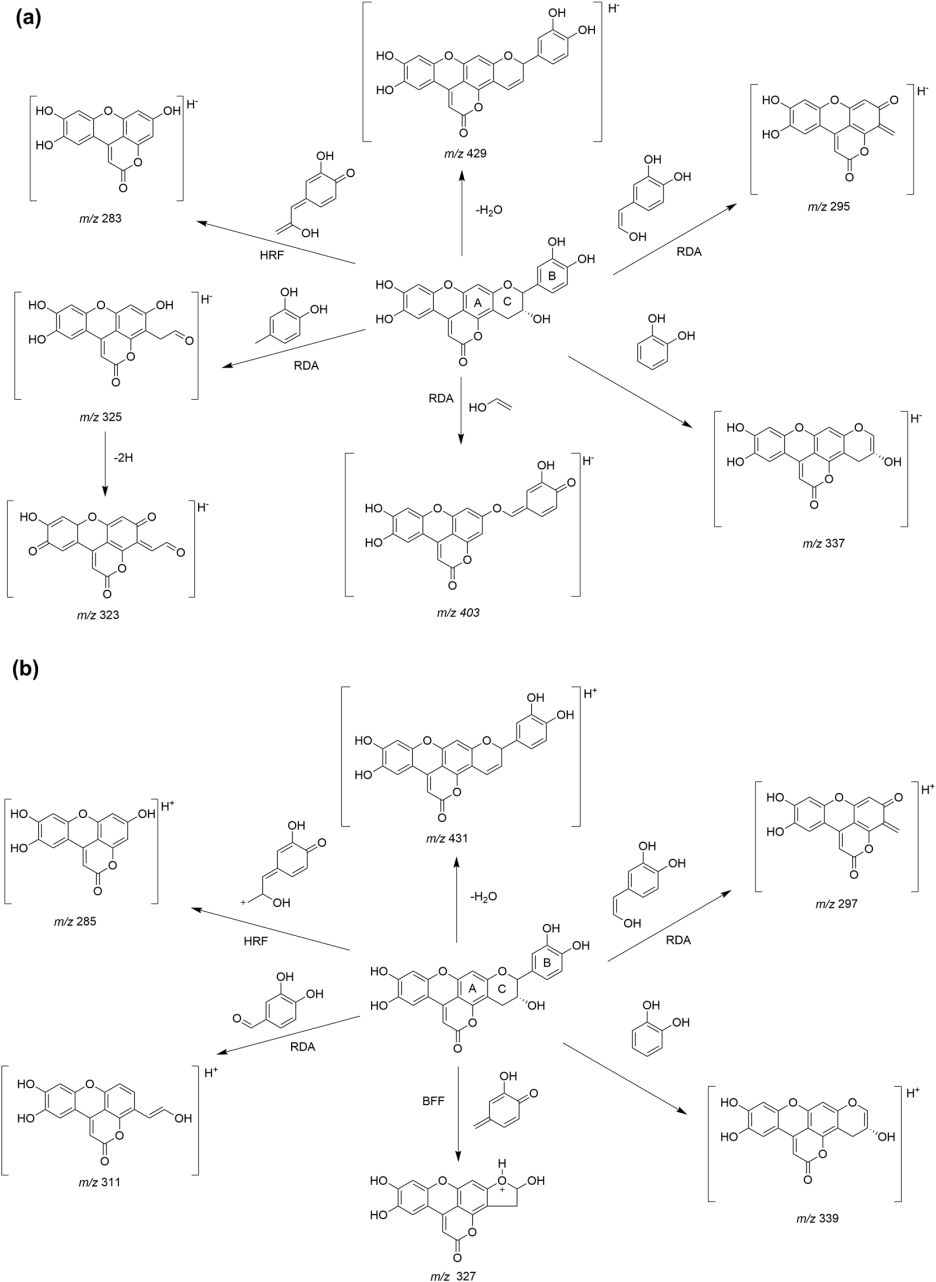
Proposed fragmentation pathway of flavonolignans in (**a**) negative and (**b**) positive ion-mode. RDA = retro-Diels-Alder fission, HRF = heterocyclic ring fission and BFF = benzofuran forming fission.

Three flavonolignans (**41**–**43**) were only found in their algycone form. Compounds **41**–**43** gave the same molecular ions at *m/z* 447.07 [M − H]^−^ and 449.08 [M + H]^+^, which were observed as base peaks (Fig. 4). MS^E^ data showed characteristic ions of catechin and epicatechin fragmentation described previously in literature^61–65^. The proposed fragmentation pathway and MS^E^ product ions of flavonolignans are shown in Fig. 6 and Supplementary Table S7. The product ions at *m/z* 429 [M-H-18]^−^ and *m/z* 431 [M + H-18]^+^ resulted from to the neutral loss of a molecule of water. Moreover, characteristic ions ^1,3^A^−^/^1,3^A^+^ (at *m/z* 295.0 [M-H-152]^−^ and 297.0 [M + H-152]^+^), ^0,2^A^+^ (at *m/z* 311.1 [M + H-138]^+^), ^1,2^A^−^ (325.1 [M-H-122]^−^ and 323.0 [M-H-122-2H]^−^) and ^2,4^A^−^ (at *m/z* 403.1[M-H-44]^−^) were produced by the Retro-Diel-Alder fission (RDA) on the C-rings. The product ions at *m/z* 337.0 [M-H-110]^−^ and 339.1 [M + H-110]^+^ are probably due to the loss of ring B, whereas *m/z* 283.0 [M-H-164]^−^ and 285.1 [M + H-164]^+^ may be formed after the elimination of rings B and C from the catechin unit by heterocyclic ring fission (HRF). In addition, the ion at *m/z* 327.1 [M + H-122]^+^ corresponds to benzofuran forming fission (BFF).

Compounds **41**, **42** and **43** were tentatively identified as vaccinin A or mururin A isomers, based on their accurate masses and fragmentation patterns. In a previous study, the presence of two flavonolignans in leaves of *C. schreberiana*, identified as cinchonain Ia and cinchonain Ib, was demonstrated^66^; however **41**, **42** and **43** (maybe derived from caffeic acid and catechin fusion through tri-substitution in ring-A) are reported here for the first time from the genus *Cecropia* as well as in the Urticaceae family.

#### Saponins

Compounds **35**, **36**, **38**, **39** and **40** showed [M-H + HCOOH]^−^ and [M + Na]^+^ base peak ions at *m/z* 711.39 and 689.38, while compounds **44**, **45**, **46** and **47** at *m/z* 695.39 and 673.39, respectively (Fig. 4). Furthermore, the negative MS spectra displayed product ions at *m/z* 503.3 [M-H-162]^−^ (**35**, **36**, **38**, **39** and **40**) and at *m/z* 487.3 [M-H-162]^−^ (**44**, **45**, **46** and **47)**, suggesting the loss of a glucose moiety (Supplementary Table S7). Compounds **35**, **36**, **44** and **45** were identified as niga-ichigoside F2 (**10a**), buergericic acid 28-*O*-glucoside (**11a**), kaji-ichigoside F1(**9a**) and tormentoside (**8a**), respectively, by comparison with isolated compounds from *C. obtusifolia*. The remaining compounds (**38**, **39**, **40**, **46** and **47**) were tentatively identified as triterpenoid saponin-*O*-hexosides based on their accurate mass and similarity on fragmentation patterns of the saponins previously described (Fig. 4). This is the first report on saponins as hexose conjugates in *C. obtusifolia*, *C. peltata*, *C. insignis* and *C. hispidissima*.

### Multivariate analysis of phytochemical composition in leaves of *Cecropia* species

**Figure 7:**
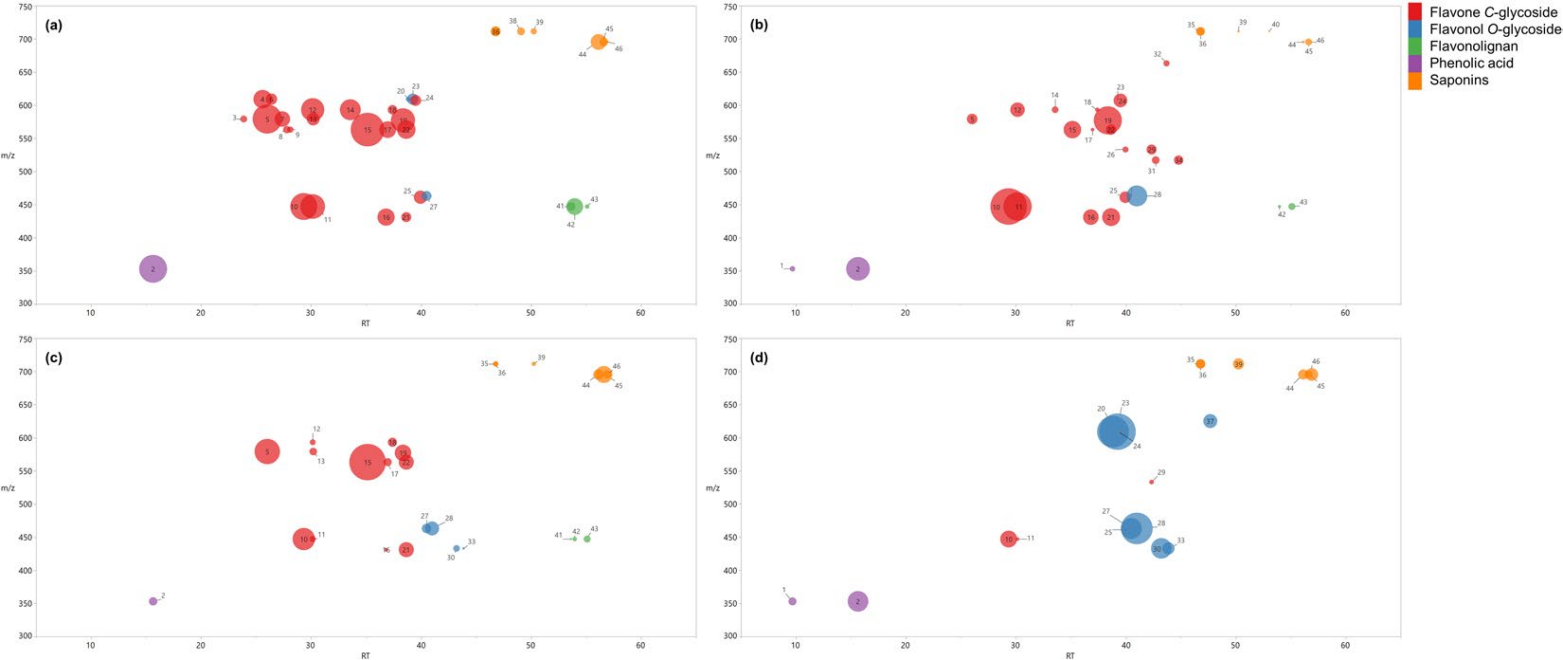
Bubble plot of C*. obtusifolia* O1 (**a**), *C. peltata* P1 (**b**), *C. insignis* I1 (**c**) and *C. hispidissima* H1 (**d**). Each bubble in the plot corresponds to a compound feature. Compounds are projected depending on their retention time (RT) (x-axis) and m/z (y-axis). The color of the bubble are based oh the chemical class. Bubble size denotes the relative abundance. Numbers are referred to compound names at Fig. 4.

A visual inspection of the phytochemical profile of *C. obtusifolia*, *C. peltata*, *C. insignis* and *C. hispidissima* samples revealed important similarities and differences regarding their phenolic acids, glycosyl flavonoids and saponins contents (see representatives bubble plots in Fig. 7). In general, the main phenolic compounds identified in all four species were chlorogenic acid (**1**), isoorientin (**10**), orientin (**11**), rutin (**23**) and saponins. Besides, we observed significant differences between *C. hispidissima* and the other species. Firstly, The most abundant flavonoids detected in *C. obtusifolia*, *C. peltata* and *C. insignis* samples were flavane *C*-glycosides flavanes (apigenin and luteolin *C*-glycosides). Secondly, flavonol mono- and diglycosides were mainly found in *C. hispidissima* samples. Lastly, it was observed that flavonolignans were not detected in *C. hispidissima* (see Fig. 7).

Qualitative analysis of the polyphenolic content in the leaves of *Cecropia* species revealed minor differences within species between the collection periods in 2015 and 2016 when samples were collected at the same locations (Table 1). As shown in Fig. 4 and Figs S47–61, similar chromatographic profiles of *C. obtusifolia* (O1 and O2), *C. peltata* (P1 and P2), *C. insignis* (I1 and I2) and *C. hispidissima* (H1 and H2) have been observed in this investigation.

**Table 1:** *Cecropia* samples: collection points in Panama.

ID	Specie	Author	Voucher specimen	Province	Coordenates	Date
O1	*C. obtusifolia*	Bertol.	2519	Panama (Cerro Azul)	9°12′33″N, 79°24′49″W	10/11/2015
O2	*C. obtusifolia*	Bertol.	2616	Panama (Cerro Azul)	9°11′10″N, 79°24′21″W	7/21/2016
O3	*C. obtusifolia*	Bertol.	2623	Panama (Cerro Azul)	9°11′10″N, 79°24′21″W	7/21/2016
O4	*C. obtusifolia*	Bertol.	2527	West Panama (Cerro Campana)	8°41′11″N, 79°55′19″W	10/17/2015
O5	*C. obtusifolia*	Bertol.	2620	West Panama (Cerro Campana)	8°41′21″N, 79°54′55″W	7/22/2016
P1	*C. peltata*	L.	2521	Panama (Camino de Cruces)	9°00′40″N, 79°35′44″W	10/11/2015
P2	*C. peltata*	L.	2625	Panama (Camino de Cruces)	9°00′40″N, 79°35′44″W	7/21/2016
P3	*C. peltata*	L.	2617	Panama (Cerro Azul)	9°11′10″N, 79°24′21″W	7/21/2016
P4	*C. peltata*	L.	2624	Panama (Cerro Azul)	9°11′10″N, 79°24′21″W	7/21/2016
I1	*C. insignis*	Liebm.	2520	Panama (Cerro Azul)	9°11′10″N, 79°24′21″W	10/11/2015
I2	*C. insignis*	Liebm.	2621	Panama (Cerro Azul)	9°11′10″N, 79°24′21″W	7/21/2016
H1	*C. hispidissima*	Cuatrec.	2518	Panama (Cerro Azul)	9°11′10″N, 79°24′21″W	10/11/2015
H2	*C. hispidissima*	Cuatrec.	2619	Panama (Cerro Azul)	9°11′10″N, 79°24′21″W	7/21/2016

**Figure 8:**
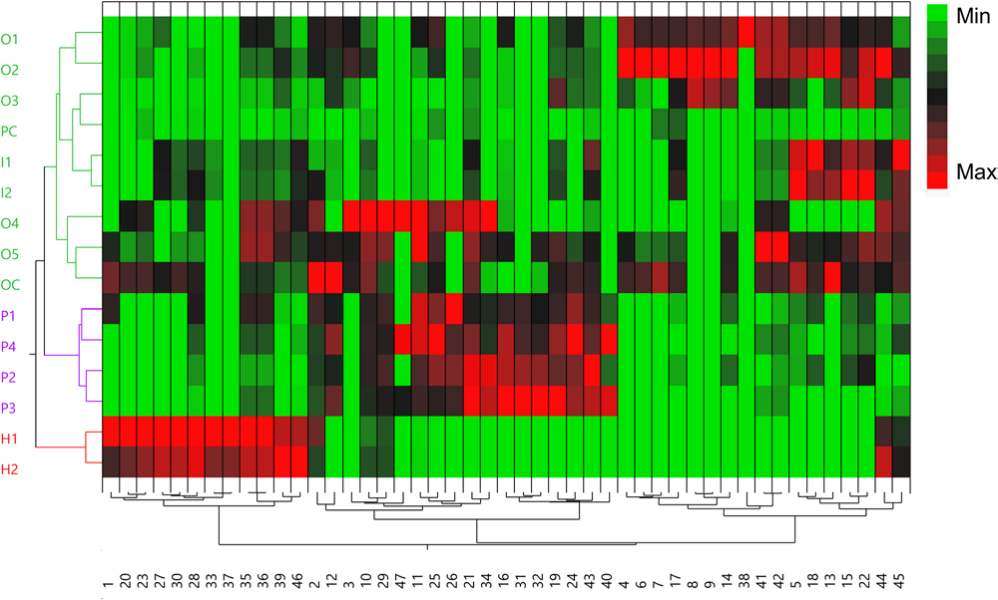
Hierarchical cluster analysis (HCA) for authentic plant species and commercial products of *Cecropia* shown as a heatmap. Colors represent the relative abundance in the samples from minimum (green) to maximum (red). Numbers are referred to compound names at Fig. 4. Clusters are presented by the same color: Cluster1 (green), Cluster 2 (purple) and Cluster 3 (red).

**Figure 9:**
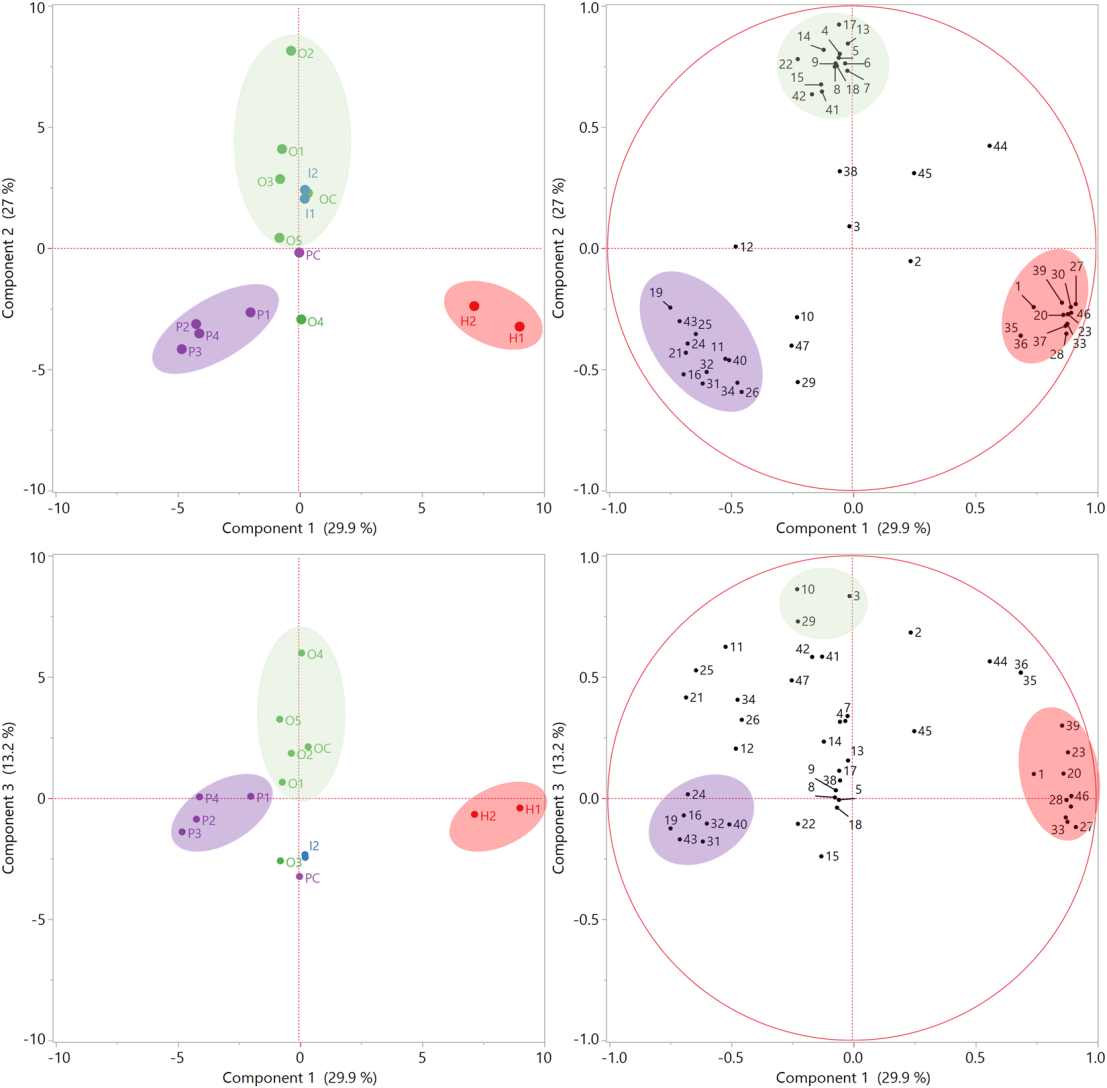
Score plots (left) and loading plots (right) from principal component analysis (PCA) for *Cecropia* species. (**a**) PC1 vs. PC2 and (**b**) PC1 vs. PC3.

A multivariate analytical approach was chosen to evaluate and characterize phytochemical differences and similarities between the four species of *Cecropia* leaves. The relative abundance of the phytochemical constituents was analysed by HCA and PCA as shown in Figs 8 and 9, respectively. The HCA generated a heatmap dendogram in wich three main clusters were observed (cluster 1, cluster and cluster 3) (Fig. 8). Inspection of cluster 1 revelead that *C. obtusifolia* (O1, O2, O3, O4, O5 and OC) and *C. insignis* (I1 and I2) were grouped together, while *C. hispidissima* samples (H1 and H2) from cluster 3 were the most distant in comparison to the other species. *C. peltata* individuals (P1-P4) were classified as belonging to cluster 2, except for the commercial product PC, which was located in cluster 1. The first three components PC1, PC2 and PC3 accounted for 70.1% of the cumulative percentage of variance of the original variables. Figure 9 shows the score (left) and loading (right) plots. The two-dimensional plots (PC1 vs PC2 and PC1 vd PC3) generated from PCA supported the clustering pattern of the HCA dendrogram (Fig. 8). Both score plots separate the species into three groups. *C. hispidissima* and *C. peltata* individuals were clustered to their respective species, while *C. obtusifolia* and *C. insignis* were mixed. PC1 separates *C. peltata* (PC1 negative scores) from *C. hispidissima* (PC1 positive scores), while PC2 and PC3 separate *C. obtusifolia* and *C. insignis* (PC2 and PC3 positive scores) from the other species (PC2 and PC3 negative scores).

Both PCA plots and the HCA dendogram (Figs 8 and 9) indicate a clear segregation of *Cecropia* species. The results obtained from the fifteen samples indicated differences regarding to the classes of compounds present. The separation observed in PCA and HCA can be explained using the loading plots for the principal component exposing the most discriminatory signals. Analysis of the loadings plot (Fig. 9) showed that chlorogenic acid (**2**), flavone *C*-glycosides/di-*C*,*O*-glycosides (**3**–**10**, **13**, **14**, **15**, **17**, and **22**) and flavonolignans (**41** and **42**) differentiate *C. obtusifolia* and *C. insignis* from the other two species. Besides, *C. peltata* (**P1**-**P4**) was found to be different from the other samples due to the high level of vitexin (**16**), isovitexin 2″-O-rhamnoside (**19**), isovitexin (**21**), *O*-methyl luteolin derivatives such as diosmetin-*C*-hexoside-*O*-deoxyhexoside (**24**) and diosmetin-*C*-hexoside (**25**), and malonylated *C*-glycosides [luteolin-*O*-malonyl-*C*-hexoside (**26**), apigenin-*O*-malonyl-*C*-hexoside (**31**), apigenin-*O*-deoxyhexoside-*O*-malonyl-*C*-hexoside (**32**), apigenin-*O*-malonyl-*C*-hexoside (**34**)]. On the other hand, *C*. *hispidissima* samples (H1 and H2) were characterized by a high relative abundance of chlorogenic acid isomer (**1**), flavonol *O*-glycosides (**20**, **23**, **27**, **28**, **30**, **33** and **37**) and saponins (niga-ichigoside F2, buergericic acid 28-*O*-glucoside, **39** and **46**).

According to Xiao *et al*.^67^, *C*-glycosylflavonoids in many cases have different pharmacokinetics and a higher potential in pharmacological activities than their corresponding *O*-glycosylflavonoids and aglycones. This fact might explain why *C. hispidissima* has not extensively been documented in traditional medicine in contrast to *C. obtusifolia*, *C. peltata* and *C. insignis*.

Phylogenetic relationships within the genus *Cecropia* revealed a pattern of divergence time between *C. hispidissima* (associated with *Neoponera* ants) and other species housing *Azteca* ants, such as *C. peltata* and *C. obtusifolia* (*Cecropia* Clade I) and *C. insignis* (*Cecropia* Clade II)^68^. This diversification is in agreement with the phytochemical profiles (patterns of flavonoids) that we have reported in this study. The present research could be particularly valuable for taxonomic classification of plants species of the genus *Cecropia* based on their phytochemical characteristics.

In this investigation the chromatographic fingerprints obtained by HPLC-DAD-MS were suitable for distinguishing between leaves of *C. hispidissima* and *C. peltata* samples from the other species. The description of novel chemical constituents associated to *Cecropia* species can be of great help to develop appropriate quality control parameters for commercial products containing these plants.

## Methods

### General Experimental Procedures

NMR spectra were recorded on a Bruker DRX-400 instrument equipped with either a 3 mm inverse broadband (BBI) probe or a 5 mm dual 1 H/13 C probe using standard Bruker pulse sequences and operating at 400 MHz for ^1^H and at 100 MHz for ^13^C NMR spectra. The spectra were processed with Topspin version 1.3 and ACD/NMR Processor Academic Edition (version 12.01, Advance Chemistry Development, Inc.). NMR spectra were recorded in DMSO-*d*_6_, MeOH-*d*_4_ or Me_2_CO-*d*_6_. For accurate mass measurements of the isolated compounds from *Cecropia obtusifolia*, a LC-HRMS method was used according to Bijttebier *et al*.^69^. For the HPLC-DAD-QTOF analyses of the *Cecropia* species, accurate mass measurements were done using a Xevo G2-XS QTof spectrometer (Waters, Milford, MA, USA) coupled with an ACQUITY LC system equipped with MassLynx version 4.1 software. For analysis, 5 µL of samples were injected on a RP-18 Kinetex column (100 mm × 2.10 mm, 2.6 μm, Phenomenex Corporation, Torrence, CA, USA). The mobile phase solvents consisted of H_2_O + 0.1% FA (A) and ACN + 0.1% FA (B), and the gradient was set as follows (min/B%): 0.0/10, 5.0/10, 20.0/15, 30/15, 40.0/25, 45.0/25, 55.0/40, 60.0/40, 65.0/100, 70.0/100, 75.0/10, 85.0/10. The flow rate was 0.6 mL/min. During the first analysis, full scan data were recorded in ESI (−) and ESI (+) mode from *m/z* 50 to 1500 and the analyzer was set in sensitivity mode (approximate resolution: 22,000 FWHM). The spray voltage was set at either +1.5 kV and −1.0 kV; cone gas flow and desolvation gas flow at 50.0 L/h and 1000.0 L/h, respectively; and source temperature and desolvation temperature at 120 °C and 550 °C, respectively. Data were also recorded using MS^E^ in the positive and negative ionization modes (two analyses per mode), and a ramp collision energy from 20 till 30 V was applied to obtain additional structural information. Leucine Encephalin was used as lock mass. DAD spectra were recorded between 190 and 500 nm. Optical rotations were recorded on a Jasco P-2000 spectropolarimeter (Easton, MD, USA) at 20 °C with Spectra ManagerTM software. The samples were dissolved in MeOH or Me_2_CO and specific rotation was determined at 589 nm with a path length of 50 mm. See Supplementary Information S.Methods for detailed information on reagents and general experimental prodecures.

### Plant Material

Leaves of *Cecropia* species were collected in the Republic of Panama. The taxonomical classification was carried out by the botanist Orlando O. Ortiz and deposited at the Herbarium of the University of Panama (Table 1). Two commercial products were purchased online: Guarumbo Tea 200 g (Nopalife, batch No. 5000 603, *Cecropia obtusifolia* leaves) (OC) and Embauba tea poweder (NaturVitae, batch No. GEMPWD11123, *Cecropia peltata* leaves) (PC).

### Phytochemical composition of *Cecropia* species

#### Extraction and isolation of chemical compounds from *Cecropia obtusifolia* (O1)

The plant material was air-dried in a general protocol oven (Heratherm^TM^, Thermo Scientific, MA, USA) at 40 °C and subsequently grounded using a mill (1.0 mm mesh size, MF 10 Basic, IKA, Staufen, Germany). The dried leaves (0.5 kg) were cleaned with *n*-hexane and consecutively macerated with 70% EtOH (v/v) at room temperature. The extracts were filtered through Whatman No. 1 filter paper. The solvent was reduced using a rotary evaporator under reduced pressure below 40 °C. The resulting reduced filtrate was lyophilized (52.2 g) and stored at −20 °C. The dried EtOH extract was dissolved in 300 mL of H_2_O and subjected to 125 g of MCI gel (MeOH in H_2_O, 0–100%) to yield eleven fractions (F1-11) (Fig. 1).

Fractions F4-6 (9.2 g) were pooled based on similar TLC profiles, and then applied onto a silica gel (200 g) column (120 × 3.5 cm). Successive elution with EtOAc: MeOH: H_2_O (60: 39: 1, 50: 48: 2, 40: 57: 3 and 30: 66: 4) yielded four fractions (F4-6: A-D). Fraction F4-6B (3.7 g) was subjected to further purification by Flash chromatography. Subfractions were collected in volumes of 25.0 mL. Fractions showing a similar pattern were combined, resulting in three flavonoid-rich fractions (B_28-29_, B_33-36_ and B_40-43_). These fractions were further purified by semi-preparative HPLC. A gradient was set as follows (min/B%): 0.0/15, 30.0/20, 50.0/25, 55.0/100, 60.0/100, 62.0/15, 67.0/15. Compounds **1a** (3.1 mg), **2a** (4.0 mg), **3a** (3.1 mg), **4a** (3.2 mg), **5a** (6.3 mg), **6a** (7.5 mg) and **7a** (7.5 mg) were obtained.

Fraction F8 (0.78 g) was separated by Sephadex LH-20 (MeOH). During the experiment the eluent was collected in sub-fractions of 5 mL (1–54). Taking into account the obtained HPLC chromatograms and the results of TLC analysis of the collected eluates, test tubes showing similar profiles were combined. This resulted in six fractions (F8: A-F). Fraction F8-B was further purified by semi-preparative HPLC. A gradient was set as follows (min/B%): 0.0/20, 45.0/35, 47.0/100, 55.0/100, 52.0/20, 57.0/20. Compounds **8a** (6.5 mg), **9a** (3.9 mg) and a mixture of **10a**-**11a** (3.0 mg) were obtained.

**Orientin (1a)**. Yellow amorphous solid; $$[\alpha ]\begin{array}{c}20\\ D\end{array}$$ =  + 20.29 (*c* 0.0031, MeOH). ^1^H and ^13^C NMR: Tables S1 and S2. HRMS spectra displayed a molecular ion at *m/z* 447.09336 [M − H]^–^ (calculated for C_21_H_19_O_11_, 447.09328) and a FA adduct ion at *m/z* 493.09885 [M-H + HCOOH]^−^ (calculated for C_22_H_21_O_13_, 493.09876) (See Table S3).

**Isoorientin-2″-O-xyloside (2a)**. Yellow amorphous solid, $$[\alpha ]\begin{array}{c}20\\ D\end{array}$$ = −10.63 (*c* 0.0038, MeOH). ^1^H and ^13^C NMR: Tables S1 and S2. HRMS *m/z* 579.13597 [M − H]^−^ (calculated for C_26_H_27_O_15_, 579.13554) (See Table S3).

**Isoorientin-4″-O-xyloside (3a)**. Yellow amorphous solid, $$[\alpha ]\begin{array}{c}20\\ D\end{array}$$ = −5.71 (*c* 0.0155, MeOH). ^1^H and ^13^C NMR: Tables S1 and S2. HRMS *m/z* 579.13596 [M − H]^−^ (calculated for C_26_H_27_O_15_, 579.13554) (See Table S3).

**Isoorientin-2″-O-rhamnoside (4a)**. Yellow amorphous solid, $$[\alpha ]\begin{array}{c}20\\ D\end{array}$$ = −20.30 (*c* 0.0032, MeOH). ^1^H and ^13^C NMR: Tables S1 and S2. HRMS *m/z* 593.15176 [M − H]^−^ (calculated for C_27_H_29_O_15_, 593.15119) (See Table S3).

**Isovitexin-2″-O-xyloside (5a)**. Yellow amorphous solid, $$[\alpha ]\begin{array}{c}20\\ D\end{array}$$ = −15.03 (*c* 0.0105, MeOH). ^1^H and ^13^C NMR: Tables S1 and S2. HRMS *m/z* 563.14069 [M − H]^−^ (calculated for C_26_H_27_O_14_, 563.14063) (See Table S3).

**Isovitexin-2″-O-glucoside (6a)**. Yellow amorphous solid, $$[\alpha ]\begin{array}{c}20\\ D\end{array}$$ = −29.31 (*c* 0.0075, MeOH). ^1^H and ^13^C NMR: Tables S1 and S2. HRMS *m/z* 593.15186 [M − H]^−^ (calculated for C_27_H_29_O_15_, 593.15119) (See Table S3).

**Isovitexin-2″-O-rhamnoside (7a)**. Yellow amorphous solid, $$[\alpha ]\begin{array}{c}20\\ D\end{array}$$ = −15.88 (*c* 0.0013, MeOH). ^1^H and ^13^C NMR: Tables S1 and S2. HRMS *m/z* 577.15641 [M − H]^−^ (calculated for C_27_H_29_O_14_, 577.15628) (See Table S3).

**Tormentic acid 28-O-glucoside (tormentoside) (8a)**. Pale yellow amorphous powder, $$[\alpha ]\begin{array}{c}20\\ D\end{array}$$ = +7.91 (*c* 0.0039, MeOH). ^1^H and ^13^C NMR: Tables S4 and S5. HRMS spectra displayed a FA adduct ion at *m/z* 695.40228 [M − H + HCOOH]^−^ (calculated for C_37_H_59_O_12_, 695.40120).

**Euscaphic acid 28-O-glucoside (Kaji-ichigoside F1) (9a)**. Pale yellow amorphous powder, $$[\alpha ]\begin{array}{c}20\\ D\end{array}$$ = +12.92 (*c* 0.0065, MeOH). ^1^H and ^13^C NMR: Tables S4 and S5. HRMS spectra displayed a FA adduct ion at *m/z* 695.40157 [M − H + HCOOH]^−^ (calculated for C_37_H_59_O_12_, 695.40120).

**Niga-ichigoside F2 (10a) and buergericic acid 28-O-glucoside (11a)**. Inseparable mixture. Pale yellow amorphous powder. ^1^H and ^13^C NMR: Tables S4 and S5. HRMS spectra displayed a molecular ion at *m/z* 665.3896 [M + H]^−^ (calculated for C_36_H_57_O_11_, 665.3906) and a FA adduct ion at *m/z* 711.3905 [M − H + HCOOH]^−^ (calculated for C_37_H_59_O_13_, 711.3961).

#### Extract preparation for phytochemical analysis

Dried leaves of *Cecropia* species were used for extraction and three independent extractions for each plant material were performed. The powdered plant material (1.0 g, particle size: ≤125 µm), weighed in a 50 mL conical tube (VWR®, Radnor, USA), was mixed with 15 mL of 70% MeOH (v/v) and extracted by sonication (42 kHz, 100 W) (Branson 3510, Danbury, USA) for 30 minutes at 65 °C. Extractions were performed three times. After treatment, the extract was centrifuged for 5 min at 3000 x g using a Heraeus Labofuge 400 Centrifuge (Langenselbold, Germany). Extraction was repeated another two times and the three supernatants were merged and diluted to 50 mL with 70% MeOH (v/v). Then, 10 mL of this solution was diluted to 20 mL with 10% MeOH (v/v). Samples were stored at 4 °C prior to analysis.

#### Total phenolic (TPC) and flavonoid (TFC) content

TPC and TFC were estimated by the Folin-Ciocalteau assay and aluminium chloride colorimetric method, respectively, as described by Rodrigues *et al*.^70^. Galic acid and quercetin were used as standards and results are reported as milligrams of standard equivalents per gram of extract dry weigth (GAE and QE, respectively; mg/g DW).

#### HPLC-DAD-QTOF-MS analysis

The samples were analysed by HPLC-DAD-QTOF as described previously in *General experimental procedures*. The sample extracts were analized two independent times both in negative and positive ionization modes.

### Data processing and statistical analysis

Results were expressed as mean ± standard deviation (SD). Raw data files acquired from the HPLC-QTOF-MS analysis were processed with MassLynx (Waters, Milford, MA, USA). A table was generated which included information on retention time, mass/charge ratio (*m/z*) and MS peak area of the compounds in the samples. Average of relative abundances (peak area/mg DW,%) was calculated. Multivariate data analysis was performed using JMP Pro 13 (SAS Institute Inc., Cary, NC, USA). Hierarchical clustering algorithm (HCA) using Euclidian distance measurements and Ward´s method without a second data standardization was carried out. The number of clusters in HCA was chosen arbitrarily. The data was subjected to PCA to visualize general clustering, trends and differences among samples.

## Supplementary information

Supplementary Information

## Data Availability

All data generated or analysed during this study are included in this published article (and its Supplementary Information Files).
