# Supplementary material for: Phytochemical characterization and comparative studies of four Cecropia species collected in Panama using multivariate data analysis
Source: Sci Rep. 2019 Feb 11;9:1763. doi: 10.1038/s41598-018-38334-4 (PMC6370824; doi:10.1038/s41598-018-38334-4)
Supplement: Supplementary file 1 — Supplementary Information [file 41598_2018_38334_MOESM1_ESM.pdf]

Phytochemical characterization and comparative studies of four *Cecropia* species collected in Panama using multivariate data analysis

Andrés Rivera-Mondragón[a,*], Sebastiaan Bijttebier[a,b], Emmy Tuenter[a], Deborah Custers[a], Orlando O. Ortíz[c], Luc Pieters[a], Catherina Caballero-George[d], Sandra Apers[a], Kenn Foubert[a]

[a]Natural Products & Food Research and Analysis (NatuRA), Department of Pharmaceutical Sciences, University of Antwerp, Universiteitsplein 1, 2610, Antwerp, Belgium

[b]Flemish Institute for Technological Research (VITO), Business Unit Separation and Conversion Technology (SCT), Mol, Belgium

[c]Herbarium PMA, Universidad de Panamá, Estafeta Universitaria, Panama City, Republic of Panama

[d]Centre of Innovation and Technology Transfer, Institute of Scientific Research and High Technology Services (INDICASAT-AIP), Building 208, City of Knowledge, Panama, Republic of Panama

*Corresponding author at:

Natural Products & Food Research and Analysis (NatuRA), Department of Pharmaceutical Sciences, University of Antwerp, Universiteitsplein 1, 2610, Antwerp, Belgium.

E-mail address: andres.riveramondragon@student.uantwerpen.be (A. Rivera-Mondragón)

S. Methods

Reagents

MeOH (HPLC grade), acetonitrile (ACN) (HPLC grade), *n*-hexane (HPLC grade), absolute EtOH (analytical reagent grade) and glacial acetic acid (analytical reagent grade) were acquired from Fisher Chemical UK Ltd. Formic acid (FA) (98+%, pure, analytical reagent grade) was obtained from Acros Organics™ (Belgium). Ultrapure water with a resistivity of 18.2 × MΩ × cm at 25 °C (Milli-Q, Waters) was used as extraction solvent and for mobile phase preparation. For the NMR experiments, DMSO-d_6 (99.9% atom D, Sigma-Aldrich USA), MeOH-d_4 (99.8% atom D, Sigma-Aldrich USA) and Me_2CO-d_6 (99.9% atom D, Sigma Aldrich Switzerland) were purchased.

General and experimental procedures

Analytical thin layer chromatography (TLC). TLC was performed on pre-coated silica gel F_{254} plates (Merck, Darmstadt, Germany), and the bands were observed under UV light (254 and 366 nm). For flavonoid analysis, TLC was performed with a mobile phase of BuOH : FA : H_2O (20 : 6 : 10). TLC plates were examined in UV light at 366 nm after spraying with a 10 g/L solution of diphenylboric acid aminoethyl ester in NeOH and subsequently spraying with a 50 g/L solution of polyethylene glycol-400 (PEG-400) in EtOH[32]. For detection of triterpenoids, the plates were sprayed with a Liebermann-Burchard reagent, containing 5 mL of acetic anhydride and 5 mL of concentrated sulfuric acid in 50 mL of absolute EtOH, and subsequently heated for 5-10 minutes at 100 °C[33].

MCI gel Column. The first isolation steps were performed by column chromatography with MCI gel CHP20P, particle size 75-150 µm (Supelco, Mitsubishi Chemical Corp.,

Bellefonte, USA), silica gel particle size 40-63 µm (Merck, Darmstadt, Germany) and Sephadex LH-20 (Pharmacia Fine Chemicals AB, Uppsala Sweden) as stationary phases.

Flash column chromatography. It was performed on a Grace Reveleris X2 system (Columbia, MD, USA) using the Reveleris® Navigator™ software. The system was equipped with a binary pump, an UV detector and a fraction collector. A pre-packed Flash Grace Reveleris C_{18} cartridge (40 g) with particle size of 40 µm, a mobile phase consisting of H_2O + 0.1% FA (A) and ACN + 0.1% FA (B) at a flow rate of 40 mL/min were used. During the run, a gradient was set as follows (min/B%): 0.0/10, 5.0/10, 25.0/15, 45/15, 53.0/38, 58.0/38, 64.0/50, 69.0/100, 75.0/100. The UV detector was set at 340 nm.

Semi-preparative HPLC system. It comprised of a sample manager, injector and collector (2767), a quaternary gradient module (2545), a System Fluidics Organizer, an HPLC-pump (515), a DAD (2998) and a Micromass Quattro mass spectrometer with TQD, all supplied by Waters (Milford, MA, USA) and equipped with a Luna C_{18} column (250 x 10.0 mm, 5 µm) (Phenomenex, Torrance, CA, USA), was used for isolation of pure compounds. As the mobile phase H_2O + 0.1% FA (A) and ACN + 0.1% FA (B) at a flow rate of 3.0 mL/min was used, a make-up flow was used containing 80% MeOH + 0.1% FA. Detection was performed at negative ion mode under the following conditions: capillary voltage, 3.50 KV; Cone voltage, 50 V; extractor voltage, 3 V; source temp. 140 °C; desolvation temp. 400°C; desolvation gas flow, 850 L/h. The UV detector was set at 340 nm. MassLynx version 4.1 was used to process the data.

Nuclear magnetic resonance (NMR). NMR spectra were recorded on a Bruker DRX-400 instrument equipped with either a 3 mm inverse broadband (BBI) probe or a 5 mm dual $^{1}H/^{13}C$ probe using standard Bruker pulse sequences and operating at 400 MHz for ^{1}H and

at 100 MHz for ^{13}C NMR spectra. The spectra were processed with Topspin version 1.3 and ACD/NMR Processor Academic Edition (version 12.01, Advance Chemistry Development, Inc.). NMR spectra were recorded in DMSO-d_6, MeOH-d_4 or Me_2CO-d_6.

Liquid Chromatography coupled with high resolution mass spectrometry (LC-HRMS). For accurate mass measurements of the isolated compounds from *Cecropia obtusifolia*, a LC-HRMS method was used according to Bijttebier et al. (2016)[34]. For analysis, 5 µL of pure compounds were injected with a CTC PAL™ autosampler (CTC Analytics) on a Waters Acquity UPLC BEH SHIELD RP18 column (3.0 mm × 150 mm, 1.7 µm; Waters) and thermostatically (40 °C) eluted with an Accela™ quaternary solvent manager and a “Hot Pocket” column oven (Thermo Fisher Scientific). The mobile phase solvents consisted of H_2O + 0.1% FA (A) and ACN + 0.1% FA (B), and the gradient was set as follows (min/B%): 0.0/0, 9.91/26, 18.51/65, 18.76/100, 20.76/100, 20.88/0, 23.00/0. For detection an orbitrap MS (Q Exactive™, Themo Fisher Scientific, San Jose, CA, USA) with Thermo Xcalibur version 3.0 software (Analysis of moderately polar phytochemicals) were used.

Table S1. ^{1}H NMR assignments (*δ* in ppm, *J* in Hz) for compounds **1a-7a**.

Position	1a	2a	3a	4a	5a	6a	7a
3	6.61, s	6.65, s	6.65, s	6.62, s	6.74, s	6.77, s	6.73, s
5 (OH)	13.17, brs	13.68, brs	13.71, brs	13.56, brs	13.66, brs	13.68, brs	13.53, s
6	6.22, s	-	-	-	-	-	-
8	-	6.44, s	6.43, s	6.46, s	6.45, s	6.47, s	6.46, s
2′	7.47, d (2.0)	7.39, d (1.8)	7.39, d (2.0)	7.37, d (2.0)	7.90, d (8.8)	7.94, d (8.8)	7.90, d (8.8)
3′	-	-	-	-	6.91, d (8.8)	6.93, d (8.8)	6.90, d (8.8)
5′	6.86, d (8.3)	6.87, d (8.0)	6.87, d (8.3)	6.84, d (8.0)	6.91, d (8.8)	6.93, d (8.8)	6.90, d (8.8)
6′	7.50, dd (8.3, 2.0)	7.42, dd (8.5, 1.8)	7.42, dd (8.3, 2.0)	7.39, dd (8.5, 2.0)	7.90, d (8.8)	7.94, d (8.8)	7.90, d (8.8)
1″	4.68, d (9.0)	4.65, d (10.0)	4.63, d (9.8)	4.63, d (9.5)	4.65, d (9.8)	4.65, d (9.8)	4.64, d (9.8)
2″	n.o.	<u>4.40</u>[b] , *4.33*[b]	3.15[b]	<u>4.38</u>, t, (8.78), *4.21*, t (9.79)	4.38[b]	4.42[b]	<u>4.38</u>, t (9.5), *4.23*, t (9.5)
3″	n.o.	3.42[b]	3.17[b]	3.34[b]	3.42[b]	3.42[b]	3.34[b]
4″	n.o.	3.25[b]	4.42[b]	3.60[b]	3.17[b]	3.15[b]	3.16[b]
5″	n.o.	3.16[b]	3.15[b]	3.12[b]	3.17[b]	3.15[b]	3.14[b]
6″	n.o.	3.68, d (11.5), 3.38[b]	3.67, d (11.5), 3.37b	3.69, d (11.5), 3.37[b]	3.68, d (11.5), 3.39[b]	3.68, d (10.5), 3.38[b]	3.67, d (11.5), 3.41[b]
1‴	-	4.23, d (6.02)	4.12 (1H, d, *J=6.02*)	<u>5.07</u>, brs, *5.00*, brs	4.23, d (6.0)	4.11[b]	<u>5.08</u>, brs, *5.00*, brs
2‴	-	3.41[b]	2.97, t (8.3)	n.o.	3.26[b]	2.94[b]	3.60[b]
3‴	-	3.16[b]	2.85, t (7.5)	3.60[b], 3.12[b]	3.3[b]	3.19[b]	3.14[b]
4‴	-	3.29[b]	3.02[b]	2.91[b]	3.42[b]	3.19[b]	2.91, t (9.2)
5‴	-	2.88[b], 3.06[b]	3.07[b], 2.54[b]	2.32[b]	3.02[b], 2.88[b]	3.51[b]	2.31, m
6‴	-	-	-	0.51, d (6.0), 0.60, d (5.7)	-	3.12[b], 2.80[b]	0.53, d (7.0), 0.61, d (5.8)

[a] NMR data (δ) were measured in DMSO-d_6. Coupling constants (J) in Hz are given in parentheses.
[b] Overlapping signals
[c] n.o.: not observed
[d] <u>Underlined</u> values: major rotamer. Values in *italics*: minor rotamer.

Table S2. ^{13}C NMR assignments (δ in ppm) for compounds **1a-7a**.

Position	1a	2a	3a	4a	5a	6a	7a
2	164.04	163.49	163.56	163.69	163.36	163.31	163.32
3	102.28	102.66	102.74	102.47	102.73	102.72	102.55
4	181.90	181.20	181.87	181.37	181.90	181.74	181.99
5	160.39	161.84	161.74	163.42	162.19	162.31	159.97
6	98.01	108.42	108.00	108.63	108.17	108.05	109.17
7	162.29	161.10	161.99	161.25	162.86	163.23	163.19
8	104.54	93.36	93.33	92.93, *94.15*	93.82, *93.12*	93.86, *93.02*	93.15
9	155.99	156.38	156.33	156.24	156.45	156.40	156.37
10	103.81	103.38	103.97	103.56	103.20	103.42	103.43
1′	121.75	121.27	121.39	121.40	121.11	121.16	120.99
2′	113.90	113.19	113.27	113.04	128.45	128.43	128.40
3′	145.89	145.82	145.75	145.95	116.02	116.00	116.13
4′	149.95	149.90	149.69	150.20	161.22	161.19	161.39, *161.25*
5′	119.35	116.06	116.05	116.10	116.02	116.00	116.13
6′	115.70	118.92	118.96	118.93	128.45	128.43	128.40
1″	73.63	71.43	71.22	71.37	71.42	71.17	71.69
2″	70.82	80.22	70.61	74.56, *75.67*	80.22	81.31	75.81, 76.20
3″	78.75	78.66	78.35	*79.68*, 80.06	78.69	78.58	80.11, 79.71
4″	70.70	72.38	80.98	70.97	70.35	70.72	70.01
5″	81.97	81.61	81.65	81.54	81.62	81.62	81.50
6″	61.66, 61.11	61.23	61.85	61.70, 61.74	61.52	61.33	61.26, 61.79
1‴	-	105.39	106.29	100.71, *100.36*	105.23	106.30	100.75, 100.40
2‴	-	66.88	76.21	70.65	72.39	74.44	70.69
3‴	-	70.79	74.17	70.34, *70.29*	71.23	72.19	70.59
4‴	-	71.42	69.31	71.61	66.89	73.36	71.69
5‴	-	64.47	65.69	68.25	64.48	66.98	68.28
6‴	-	-	-	17.58, *17.85*	-	58.43	17.60, *17.82*

[a] NMR data (δ) were measured in DMSO-d_6.
[b] Underlined values: major rotamer. Values in italics: minor rotamer.

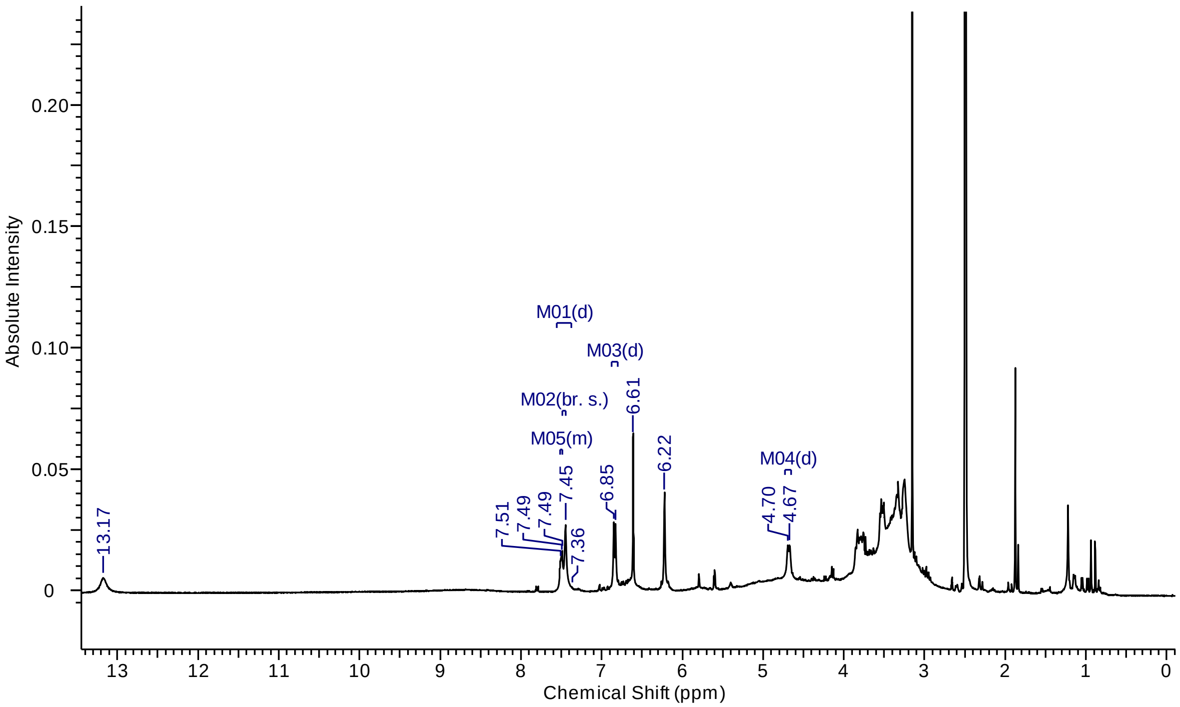

Figure S1. ^{1}H-NMR spectrum of orientin (**1a**) recorded in DMSO-d_6.

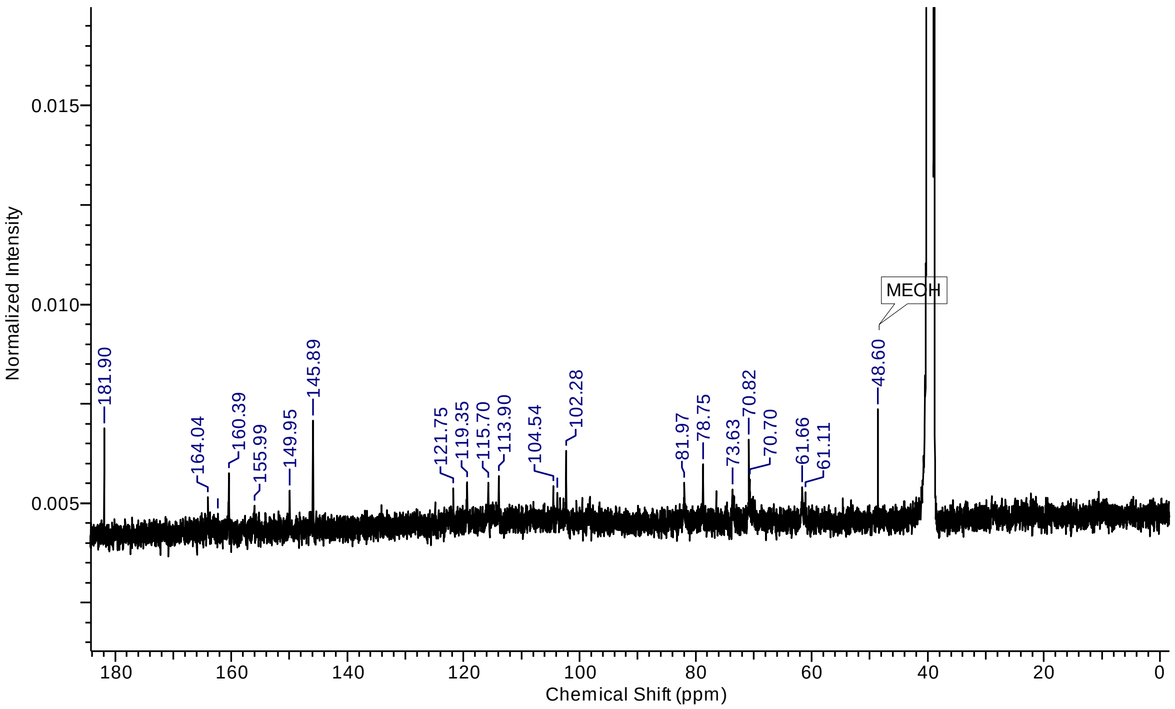

Figure S2. ^{13}C-NMR spectrum of orientin (**1a**) recorded in DMSO-d_6.

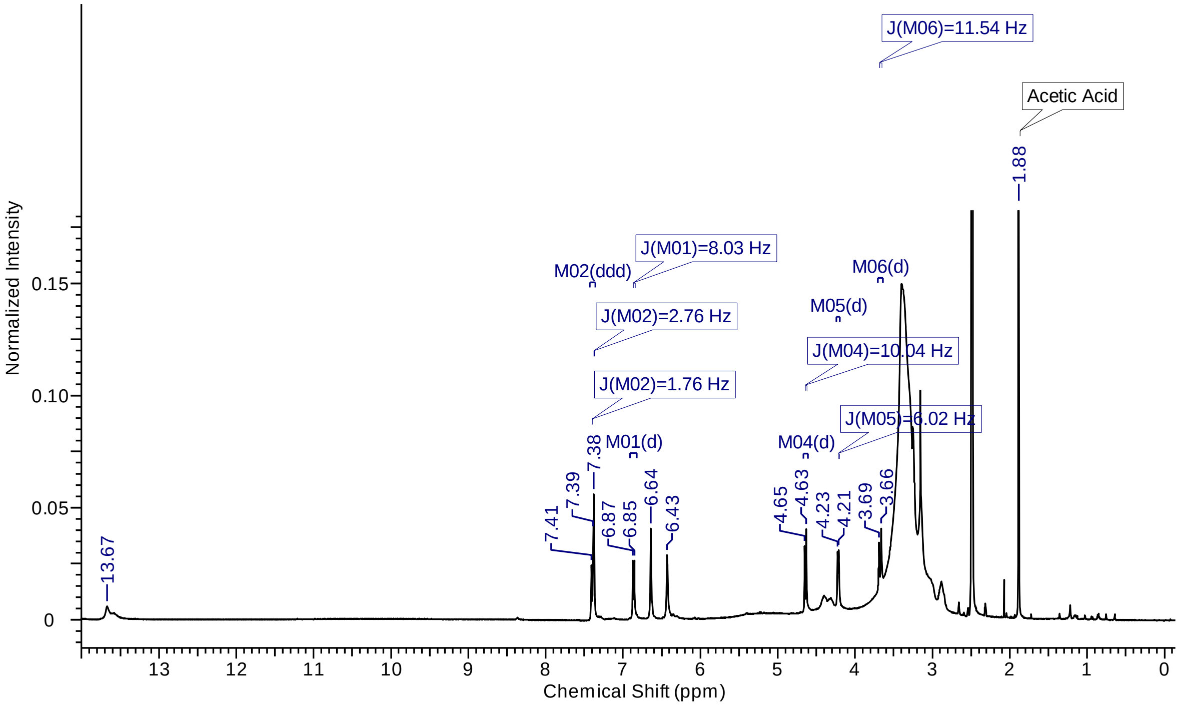

Figure S3. ^{1}H-NMR spectrum of isoorientin-2″-*O*-xyloside (**2a**) recorded in DMSO-d_6.

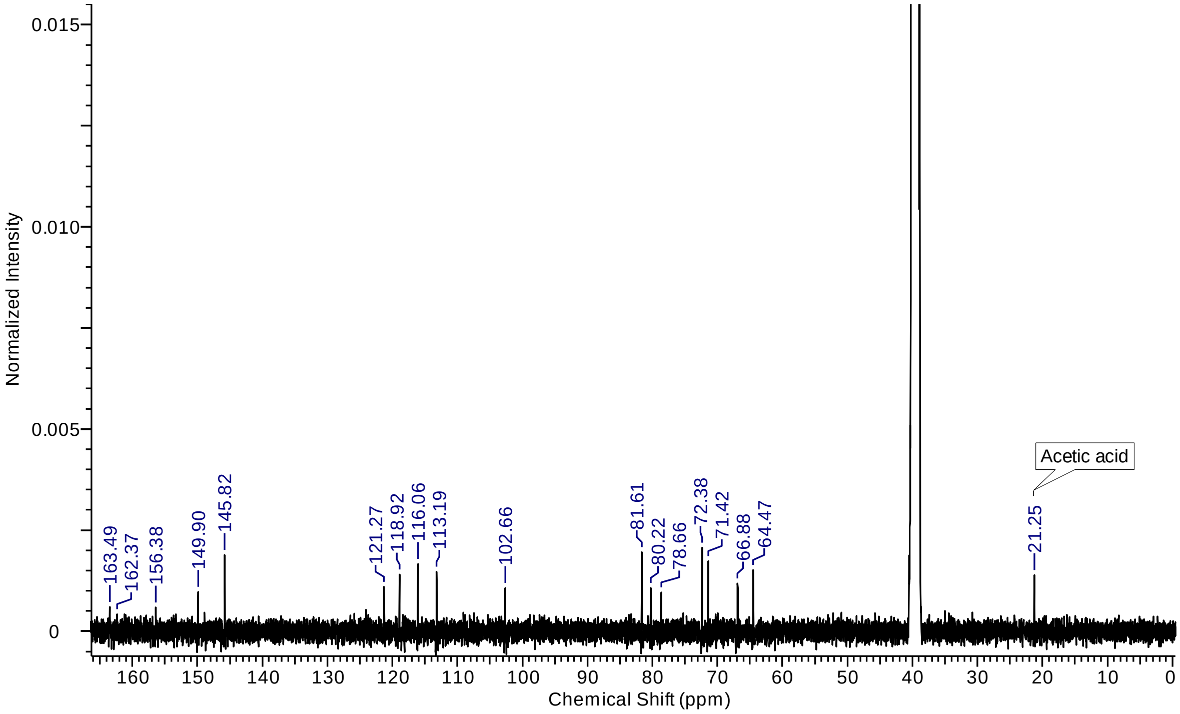

Figure S4. ^{13}C-NMR spectrum of isoorientin-2″-*O*-xyloside (**2a**) recorded in DMSO-d_6.

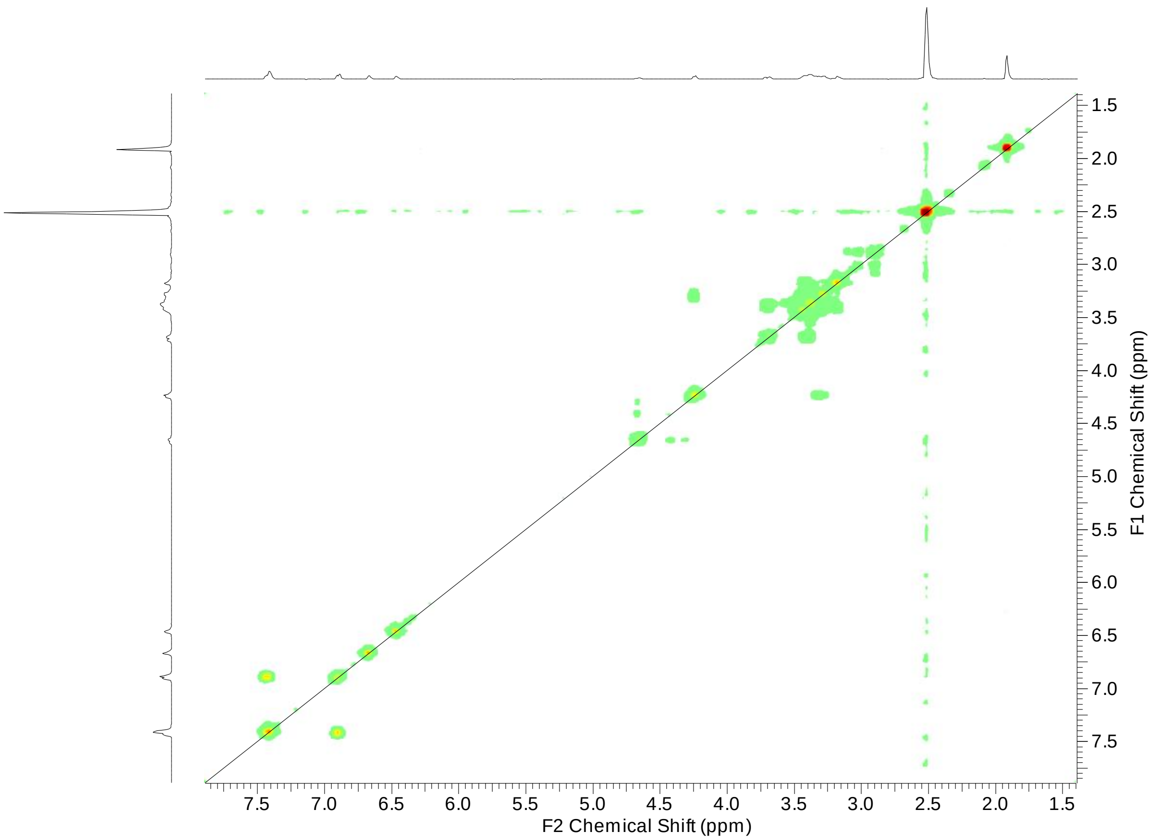

Figure S5. COSY spectrum of isoorientin-2″-*O*-xyloside (**2a**) recorded in DMSO-d_6.

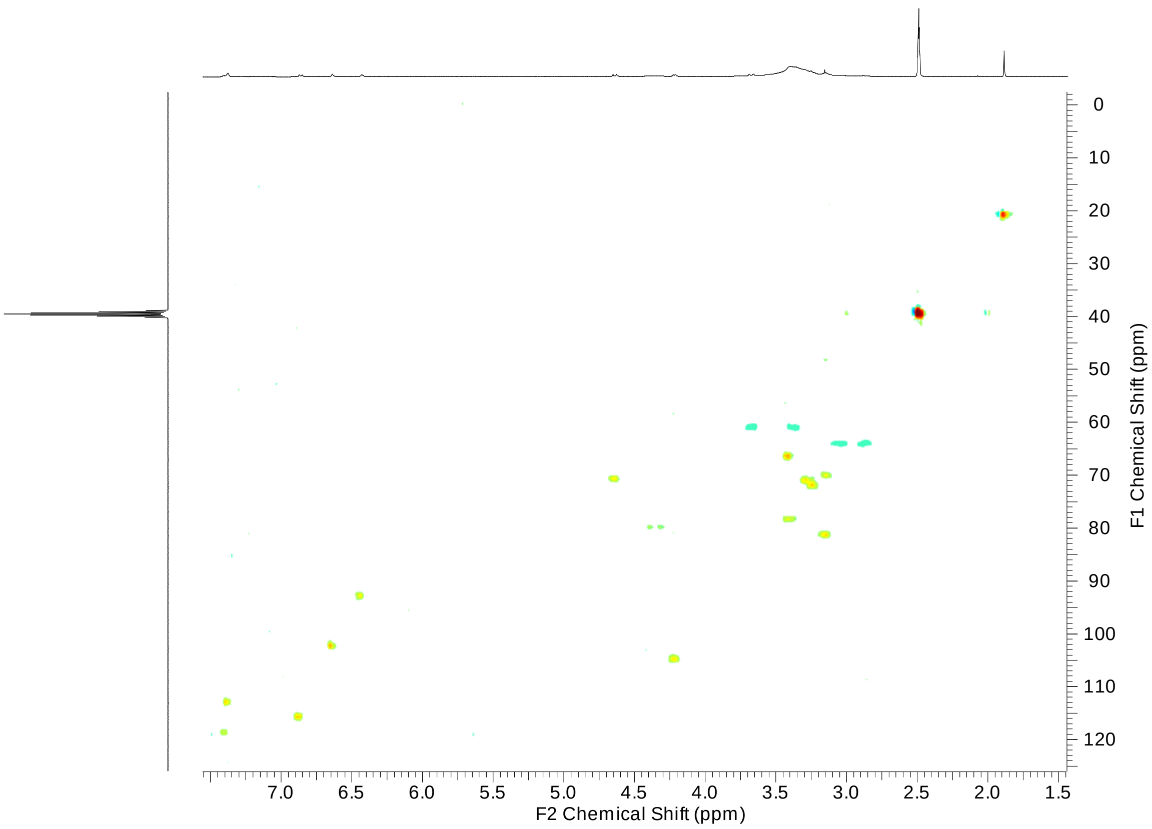

Figure S6. HSQC spectrum of isoorientin-2″-*O*-xyloside (**2a**) recorded in DMSO-d_6.

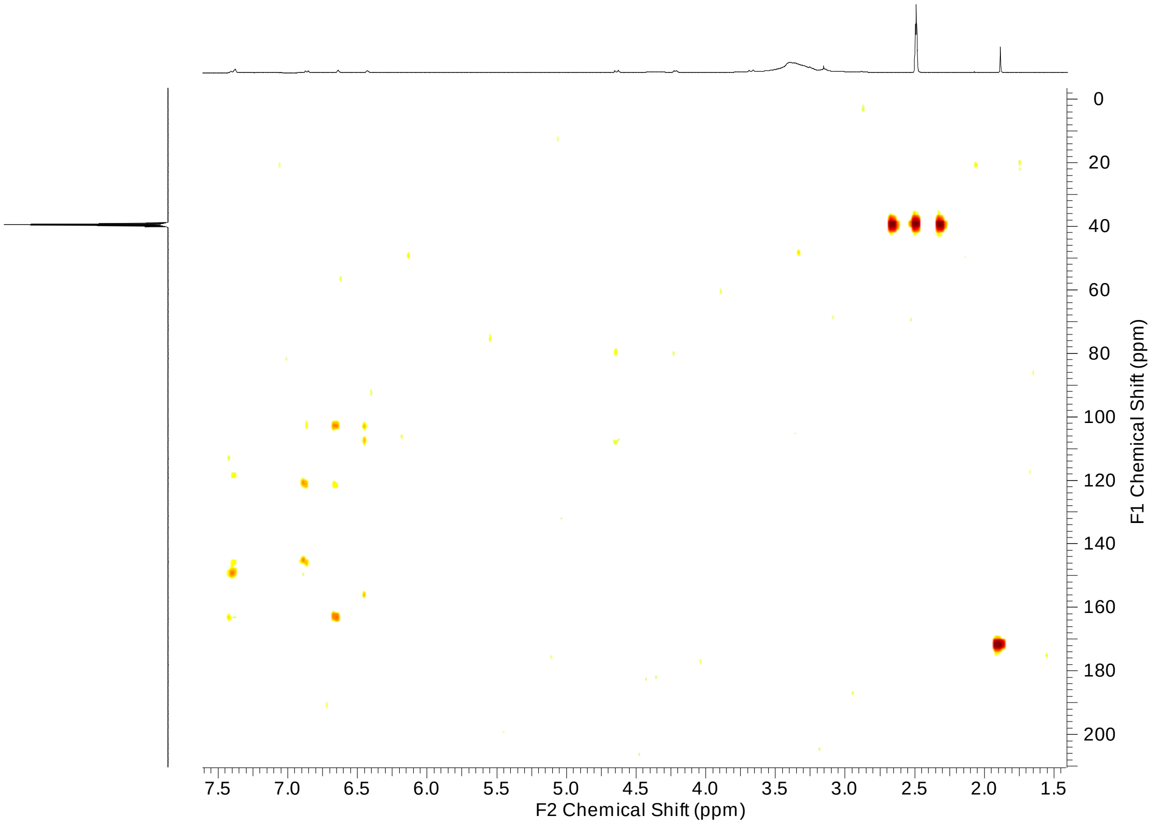

Figure S7. HMBC spectrum of isoorientin-2″-*O*-xyloside (**2a**) recorded in DMSO-d_6.

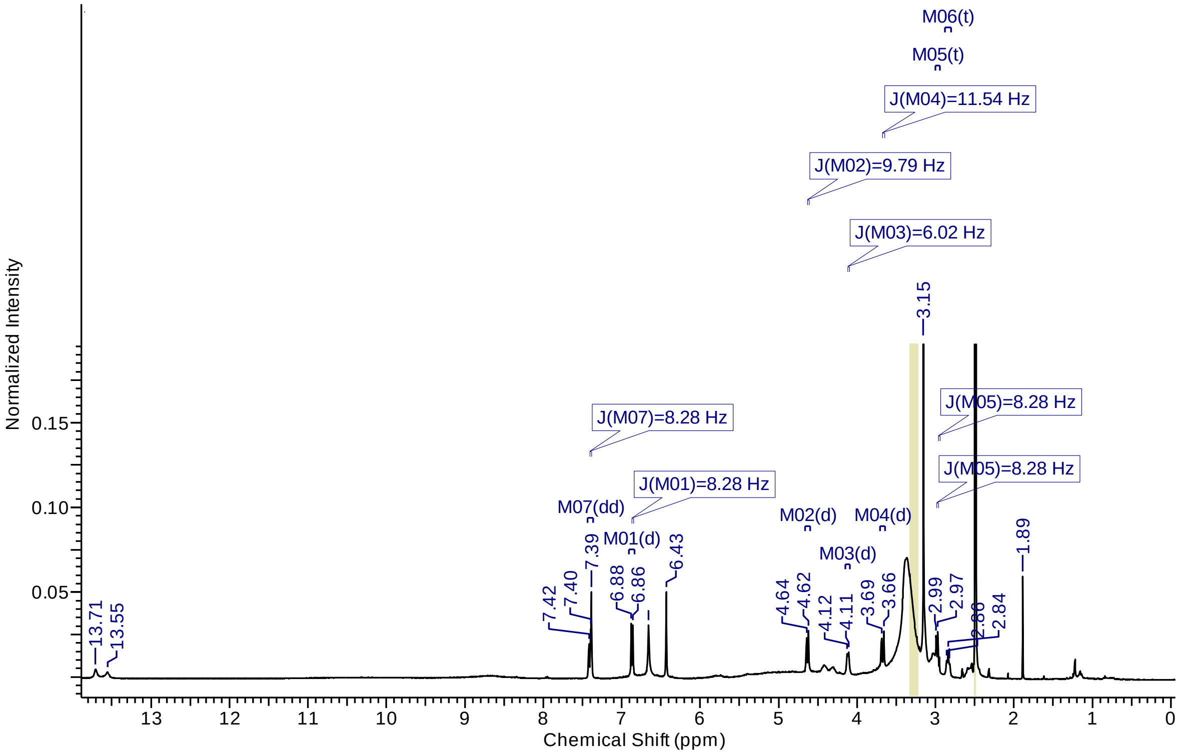

Figure S8. ^{1}H-NMR spectrum of isoorientin-4″-*O*-xyloside (**3a**) recorded in DMSO-d_6.

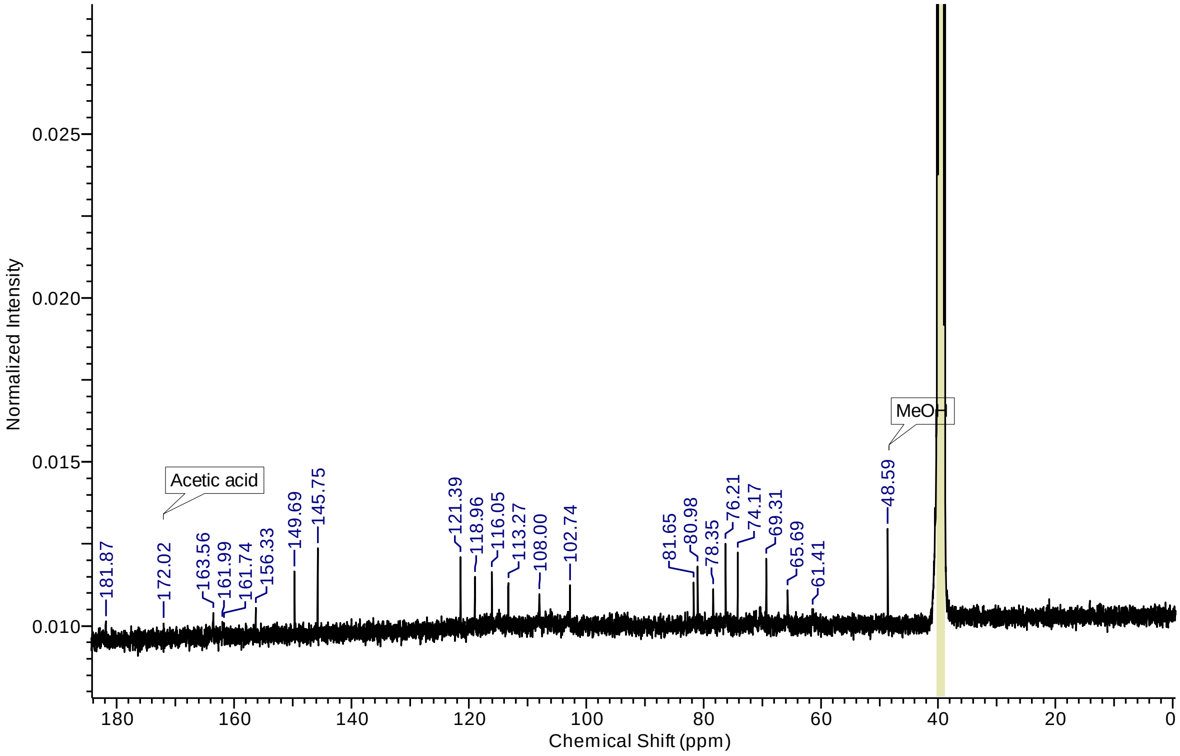

Figure S9. ^{13}C-NMR spectrum of isoorientin-4″-*O*-xyloside (**3a**) recorded in DMSO-d_6.

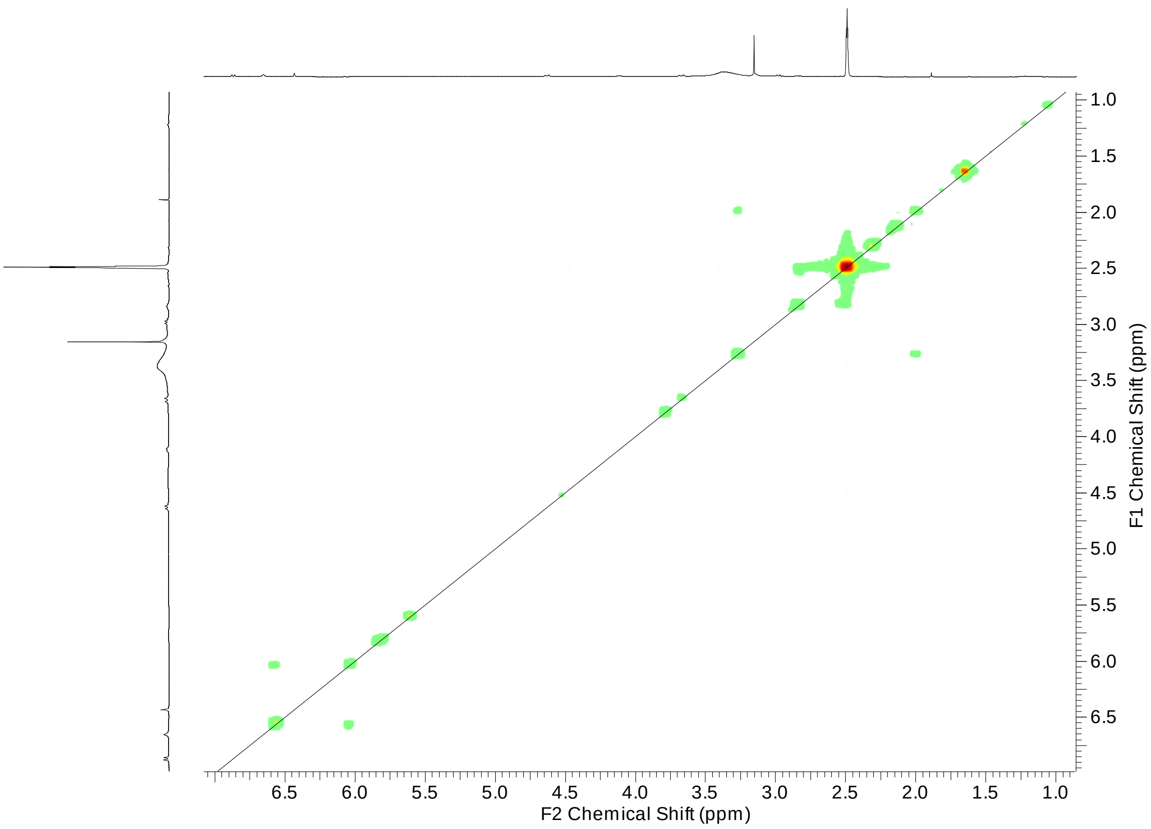

Figure S10. COSY spectrum of isoorientin-4′′-*O*-xyloside (**3a**) recorded in DMSO-d_6.

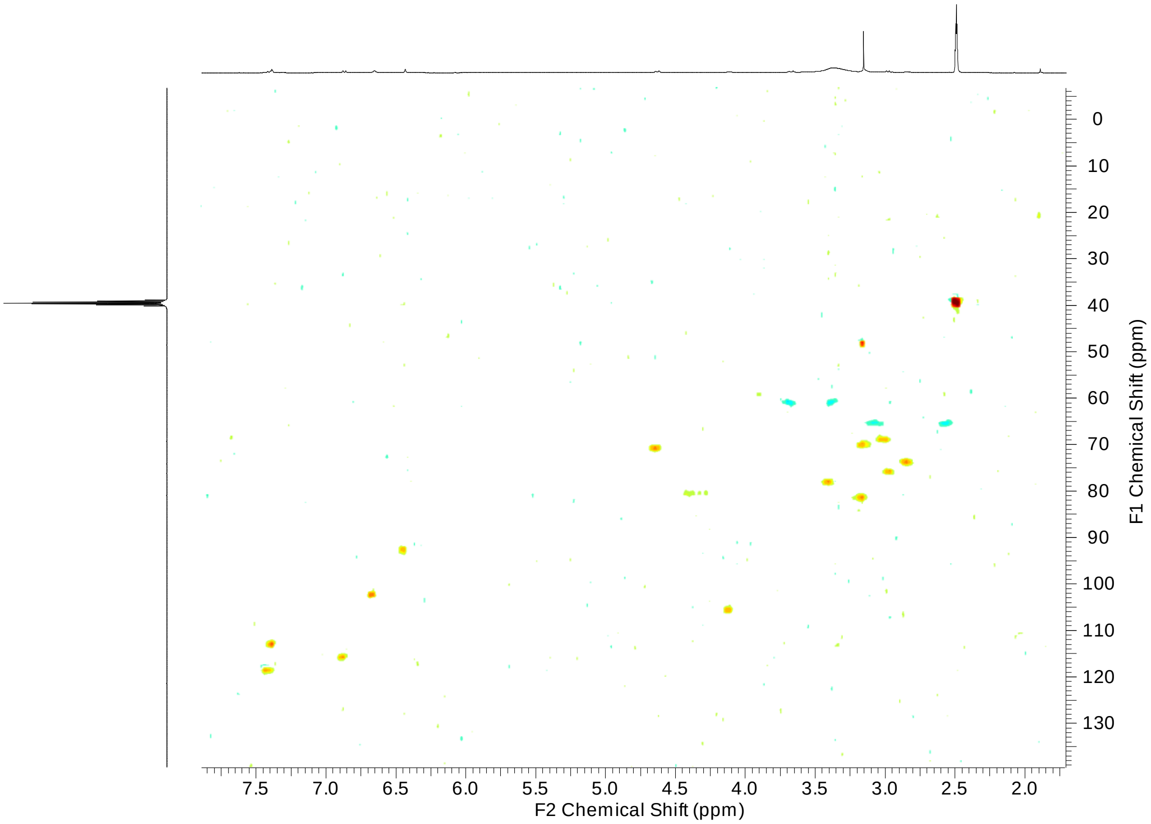

Figure S11. HSQC spectrum of isoorientin-4″-*O*-xyloside (**3a**) recorded in DMSO-d_6.

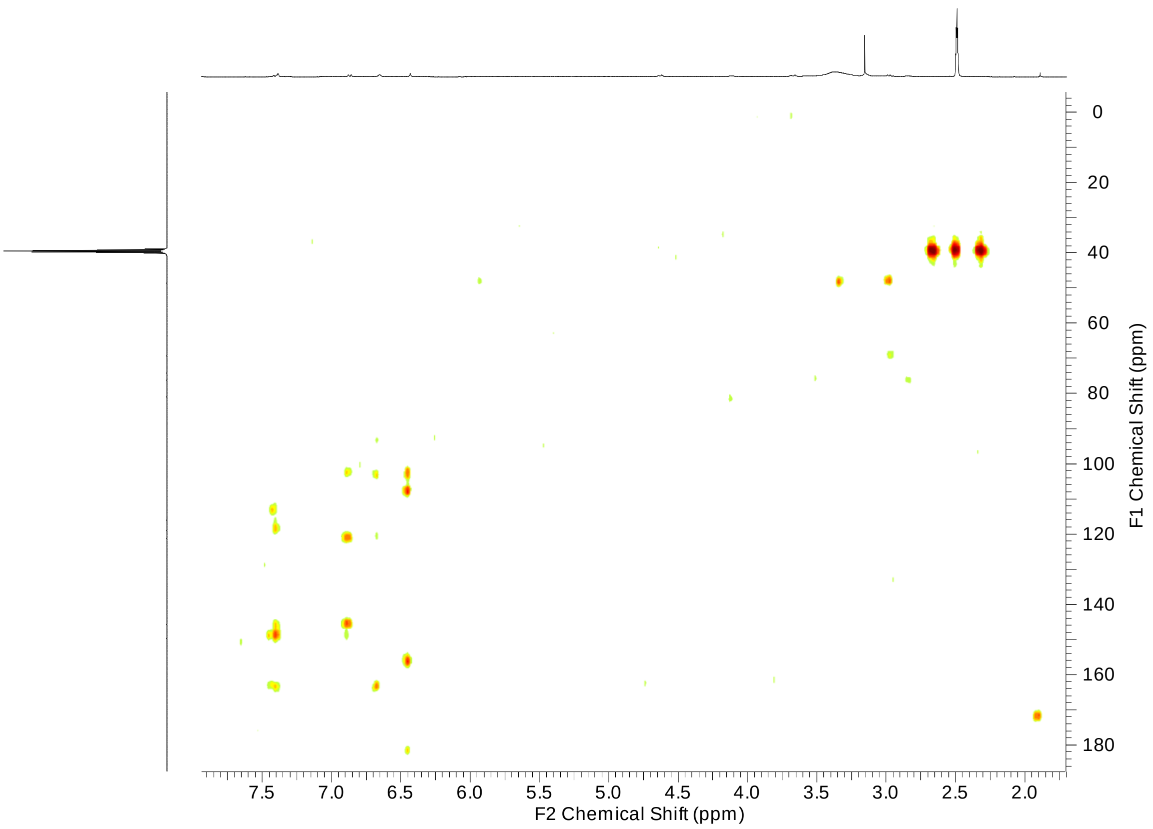

Figure S12. HMBC spectrum of isoorientin-4′′-*O*-xyloside (**3a**) recorded in DMSO-d_6.

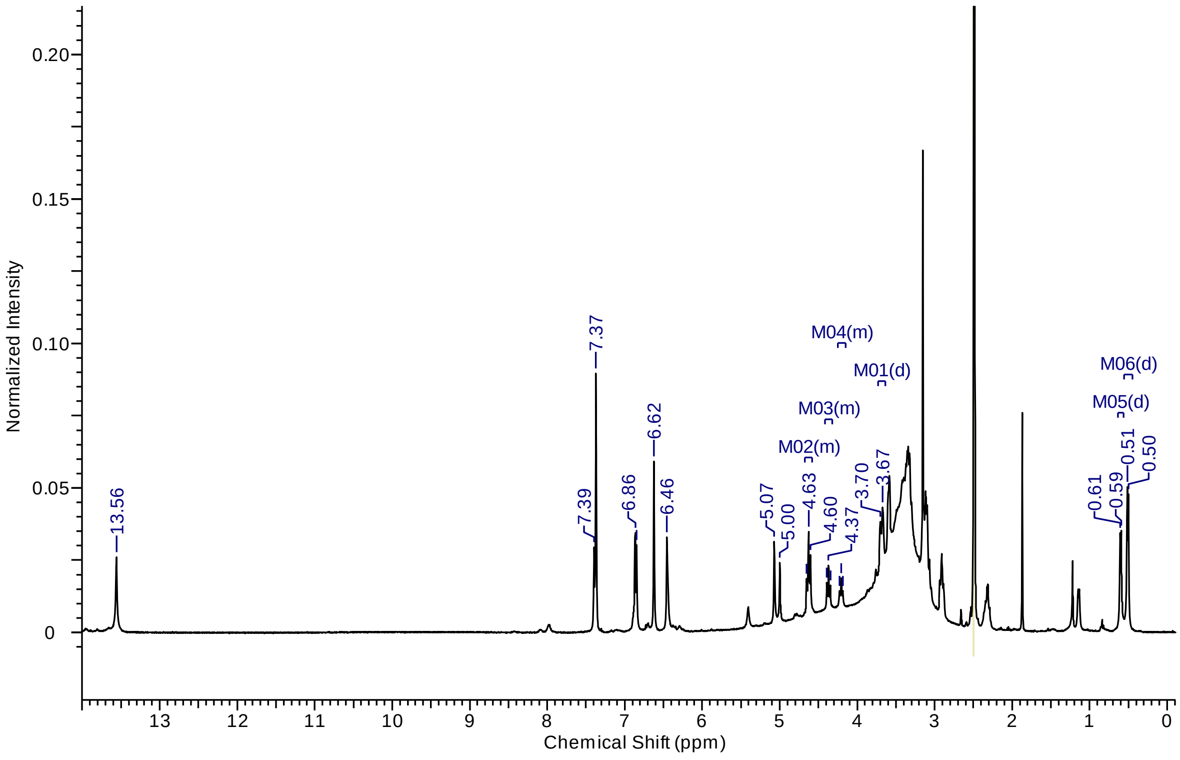

Figure S13. ^{1}H-NMR spectrum of isoorientin-4′′-*O*-xyloside (**4a**) recorded in DMSO-d_6

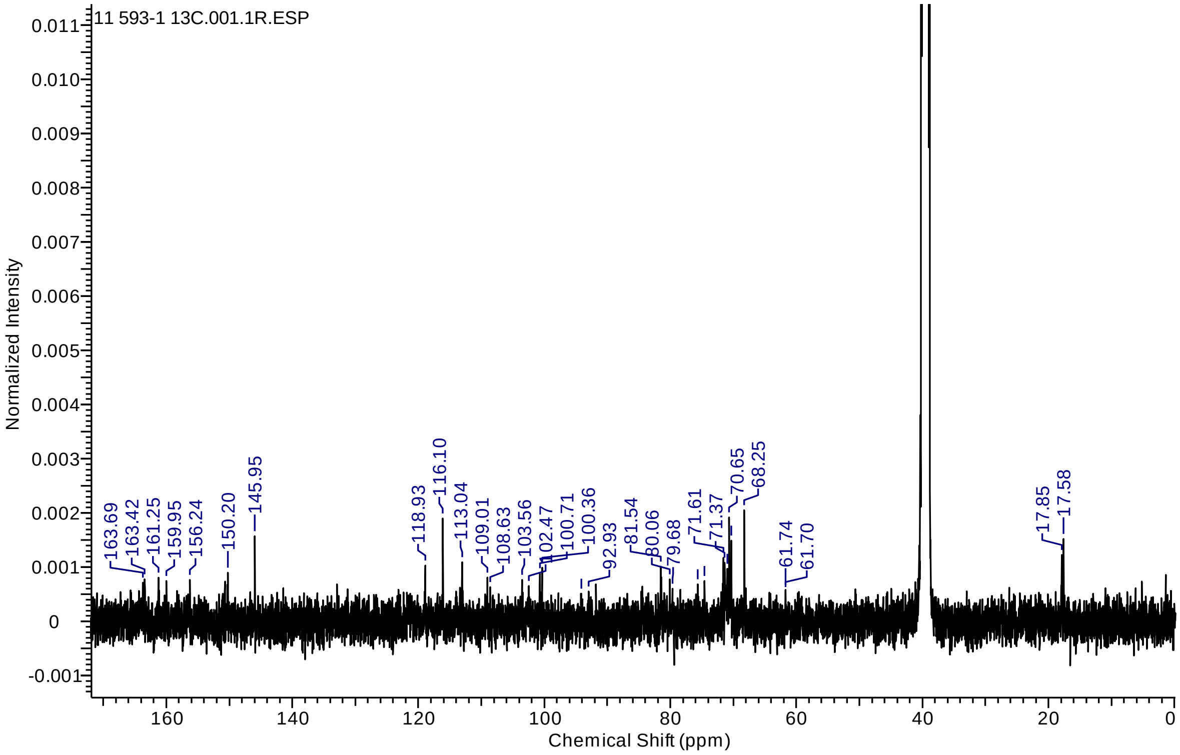

Figure S14. ^{13}C-NMR spectrum of isoorientin-4′′-*O*-xyloside (**4a**) recorded in DMSO-d_6.

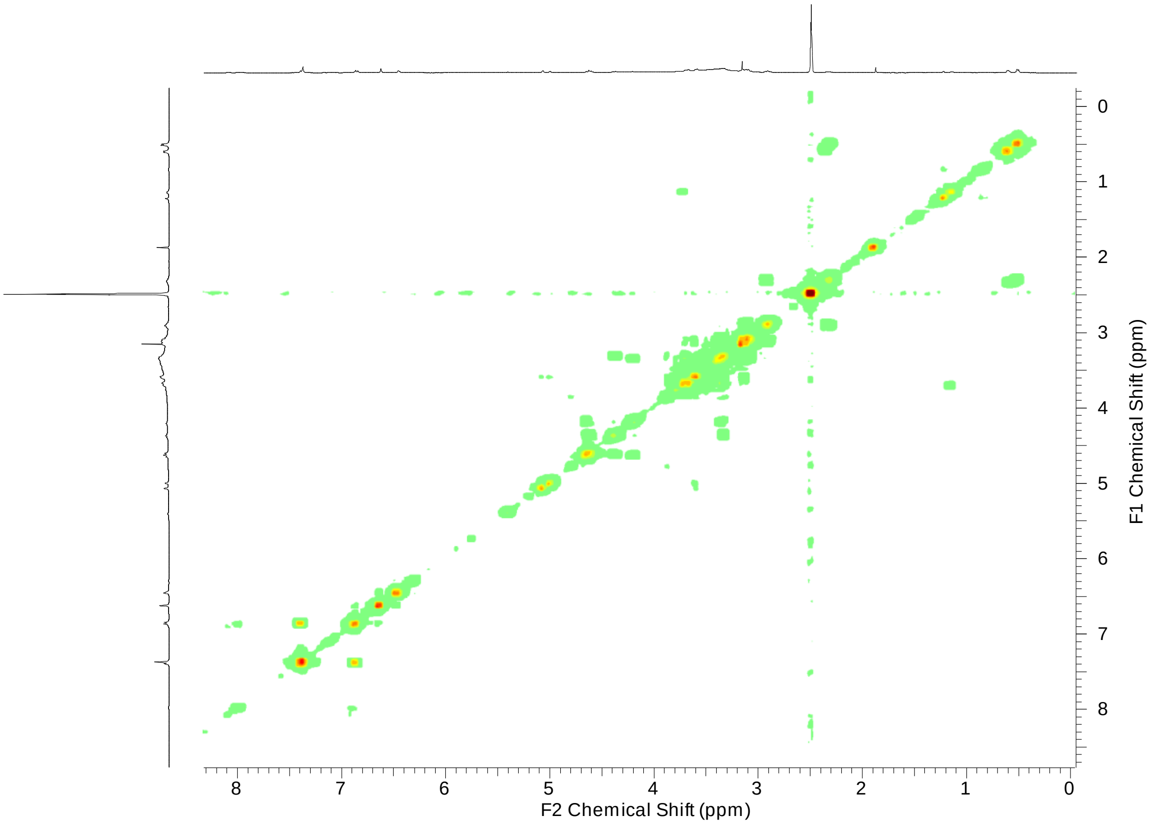

Figure S15. COSY spectrum of isoorientin-4′′-*O*-xyloside (**4a**) recorded in DMSO-d_6.

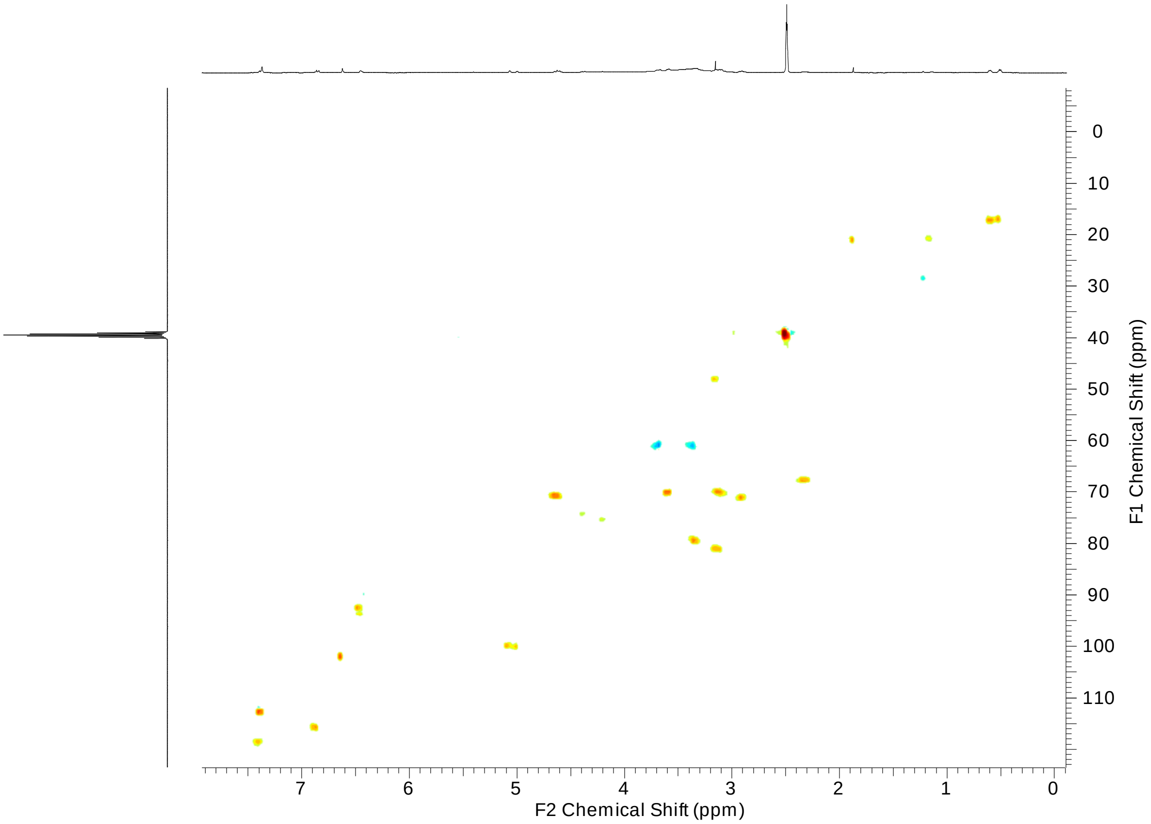

Figure S16. HSQC spectrum of isoorientin-4″-*O*-xyloside (**4a**) recorded in DMSO-d_6.

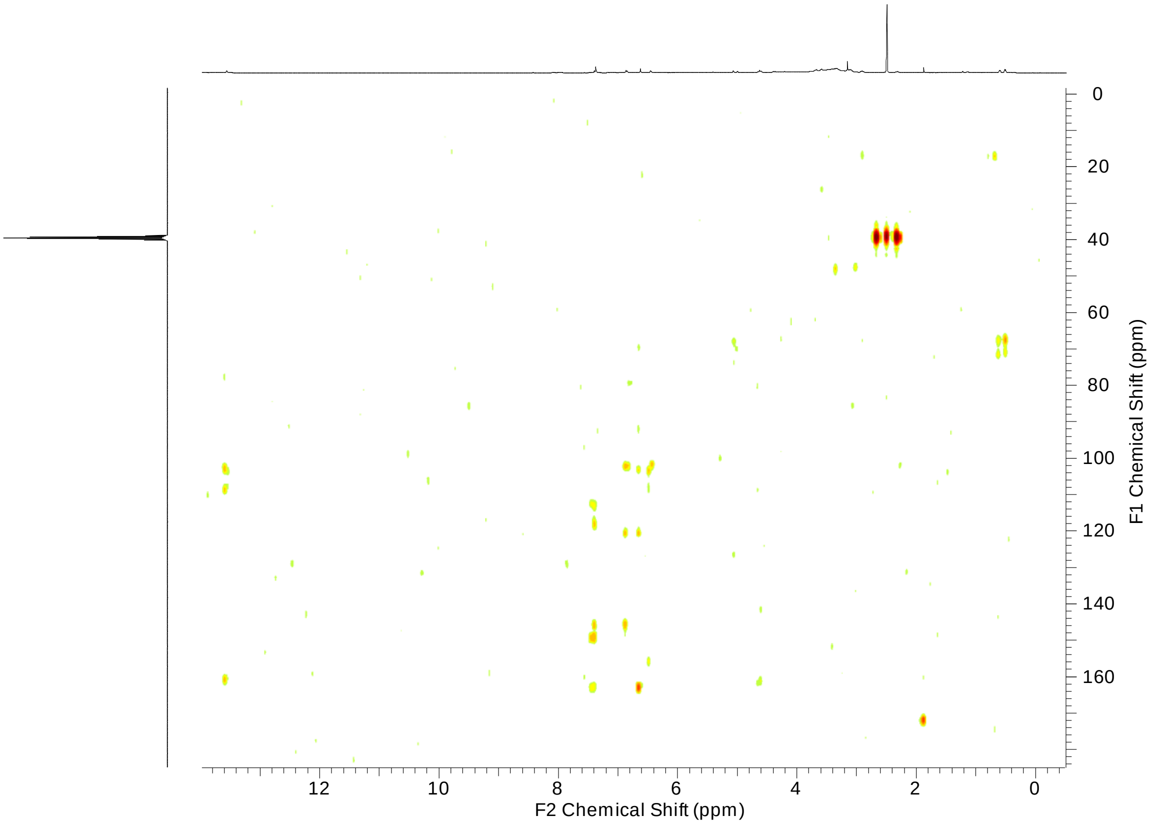

Figure S17. HMBC spectrum of isoorientin-4′′-*O*-xyloside (**4a**) recorded in DMSO-d_6.

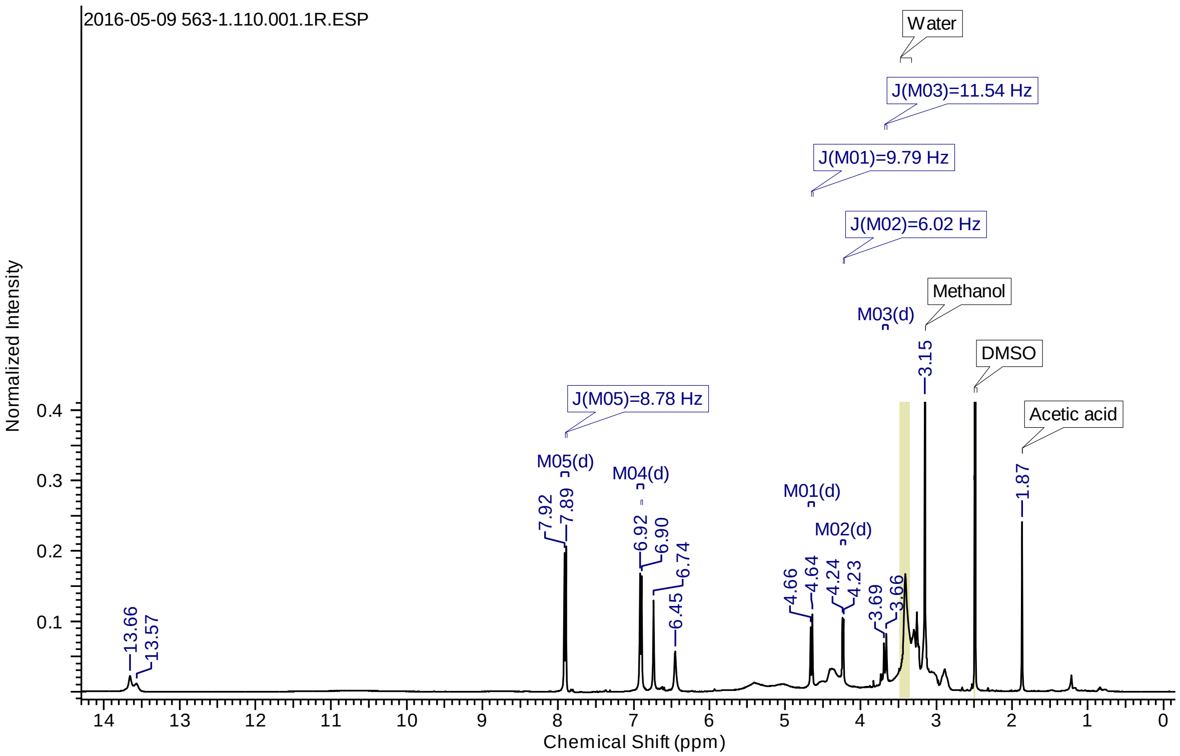

Figure S18. ^{1}H-NMR spectrum of isovitexin-2″-*O*-xyloside (**5a**) recorded in DMSO-d_6.

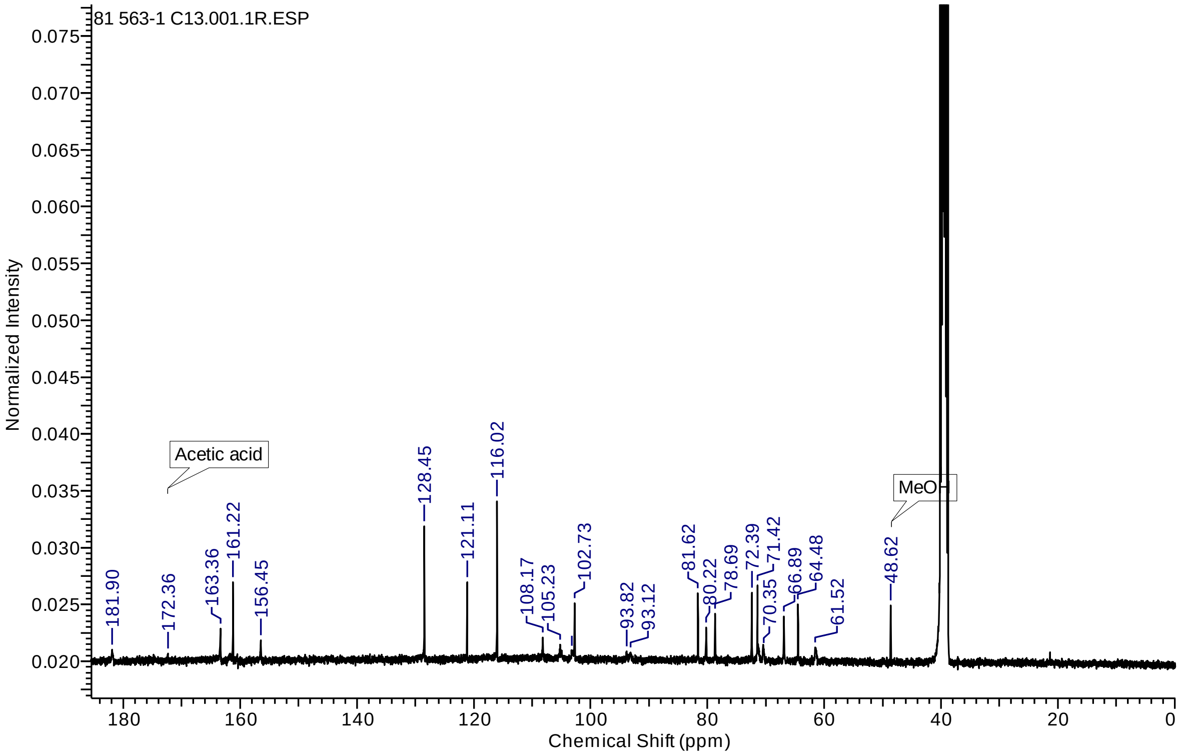

Figure S19. ^{13}C-NMR spectrum of isovitexin-2″-*O*-xyloside (**5a**) recorded in DMSO-d_6.

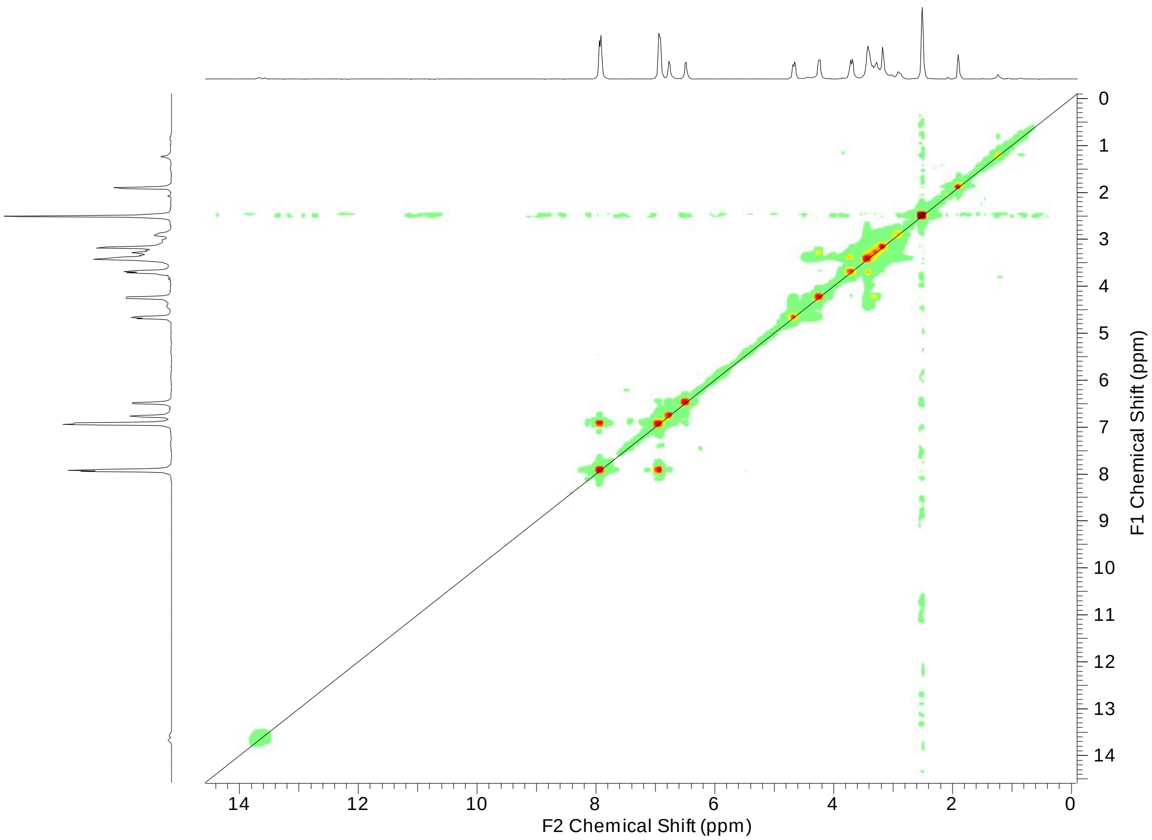

Figure S20. COSY spectrum of isovitexin-2″-*O*-xyloside (**5a**) recorded in DMSO-d_6.

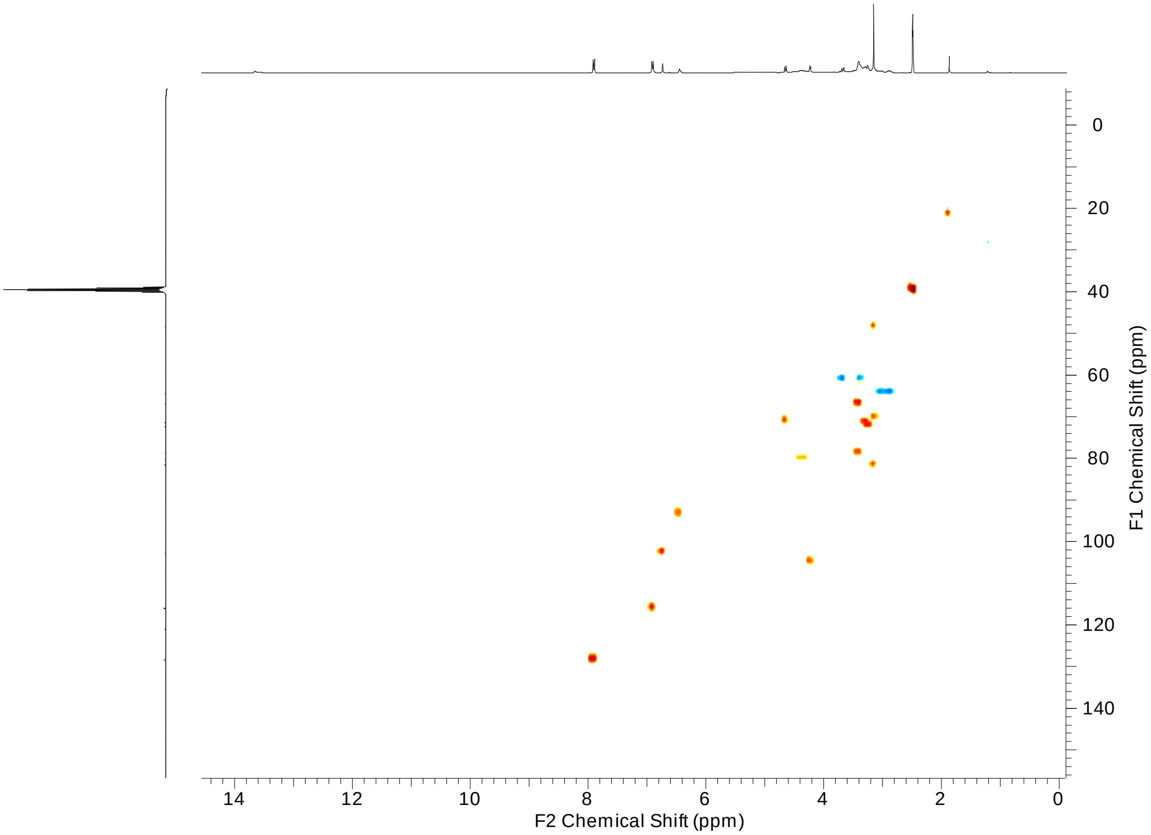

Figure S21. HSQC spectrum of isovitexin-2″-*O*-xyloside (**5a**) recorded in DMSO-d_6.

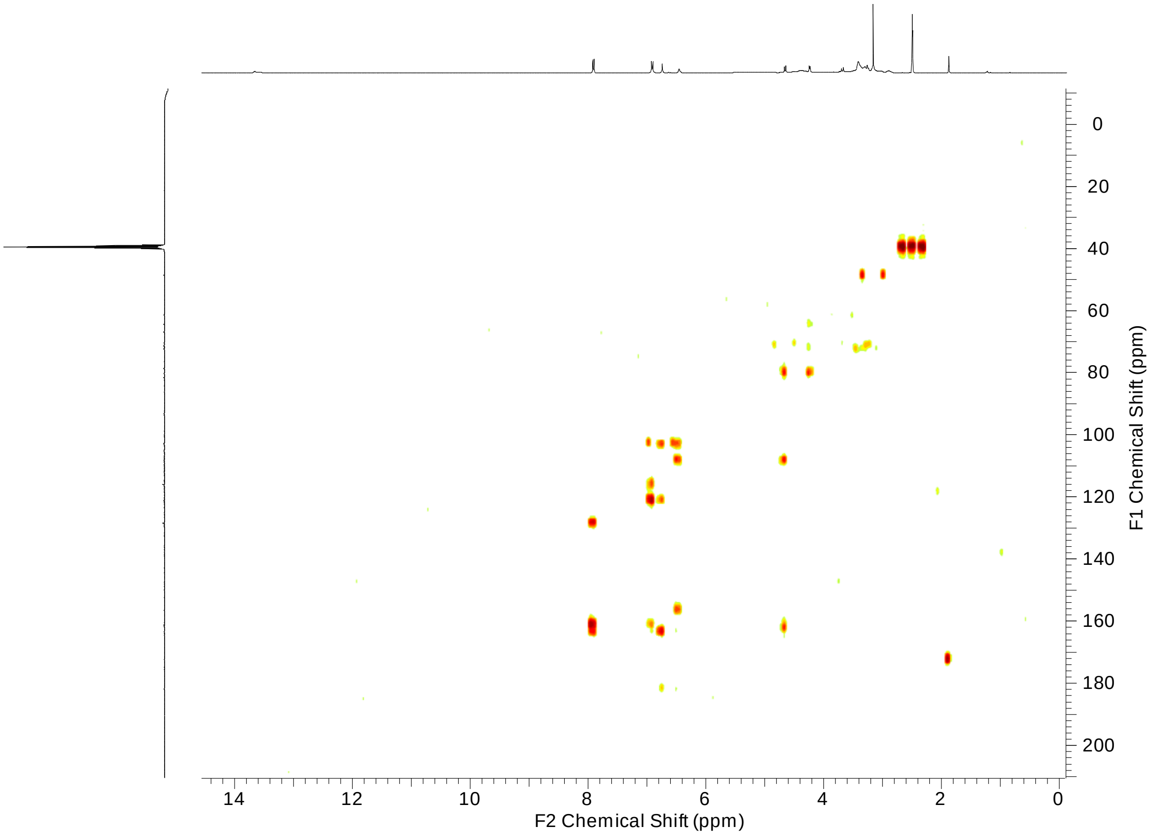

Figure S22. HMBC spectrum of isovitexin-2″-*O*-xyloside (**5a**) recorded in DMSO-d_6.

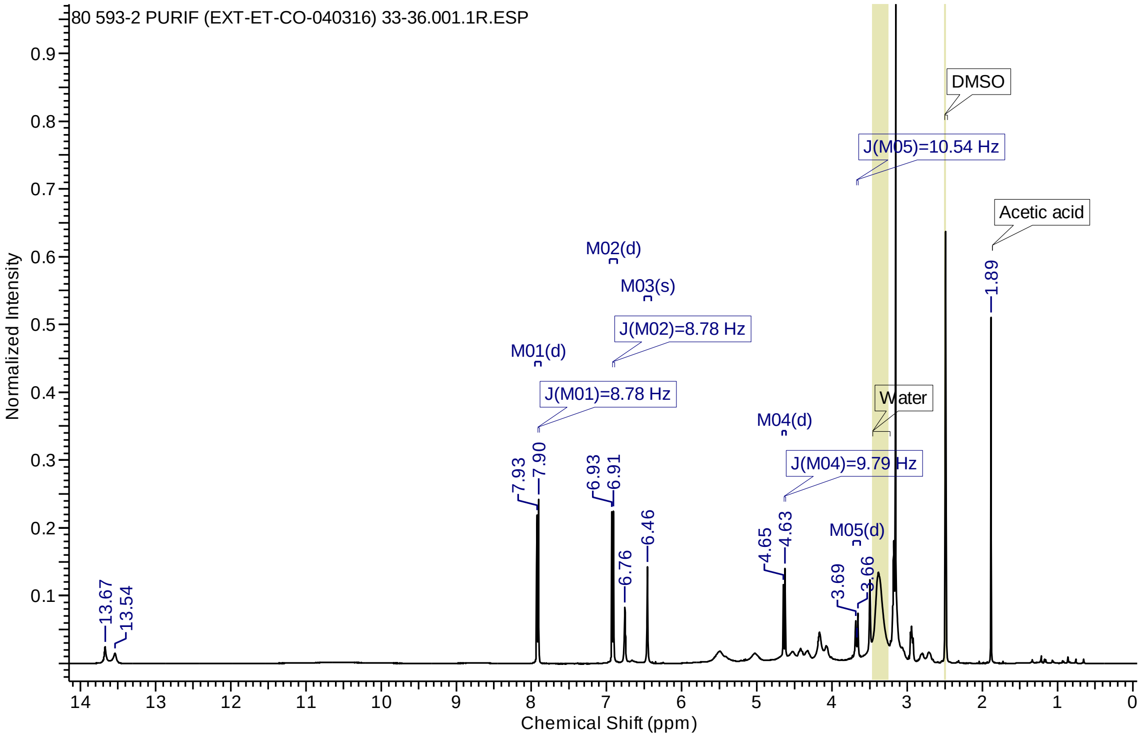

Figure S23. ^{1}H-NMR spectrum of isovitexin-2′′-*O*-glucoside (**6a**) recorded in DMSO-d_6.

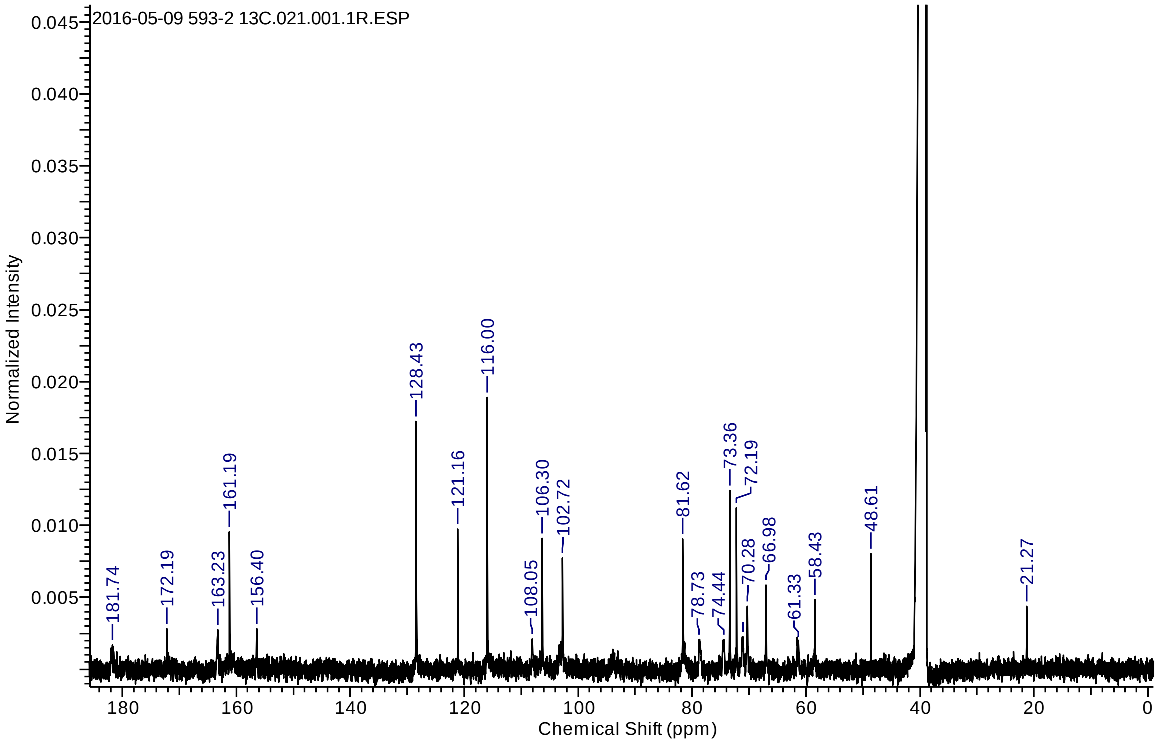

Figure S24. ^{13}C-NMR spectrum of isovitexin-2′′-*O*-glucoside (**6a**) recorded in DMSO-d_6.

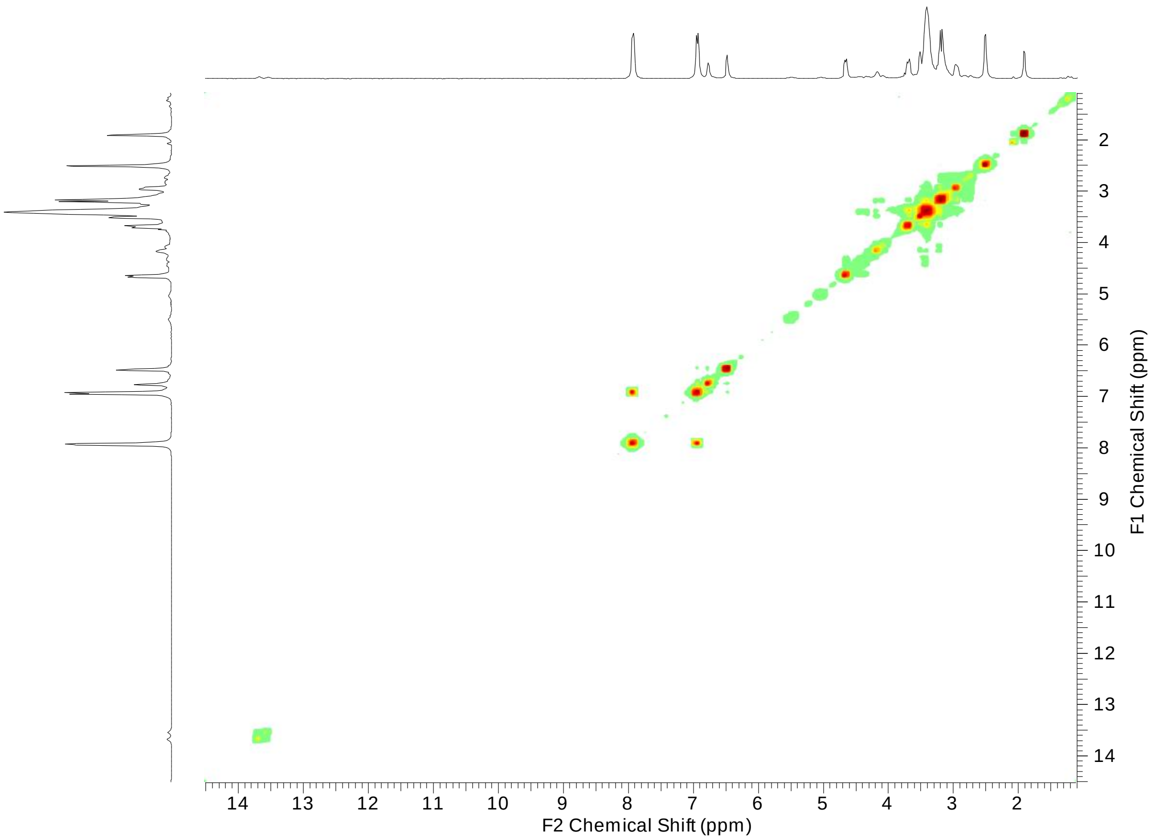

Figure S25. COSY spectrum of isovitexin-2′′-*O*-glucoside (**6a**) recorded in DMSO-d_6.

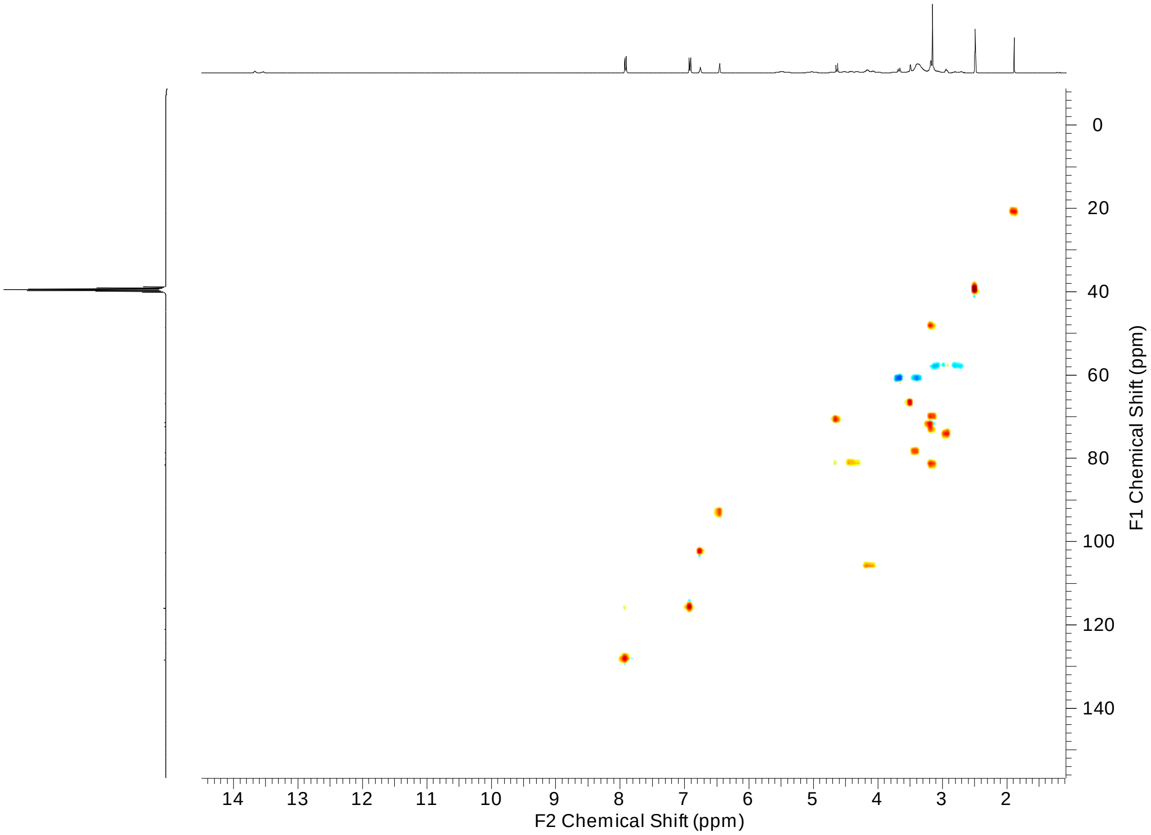

Figure S26. HSQC spectrum of isovitexin-2″-*O*-glucoside (**6a**) recorded in DMSO-d_6.

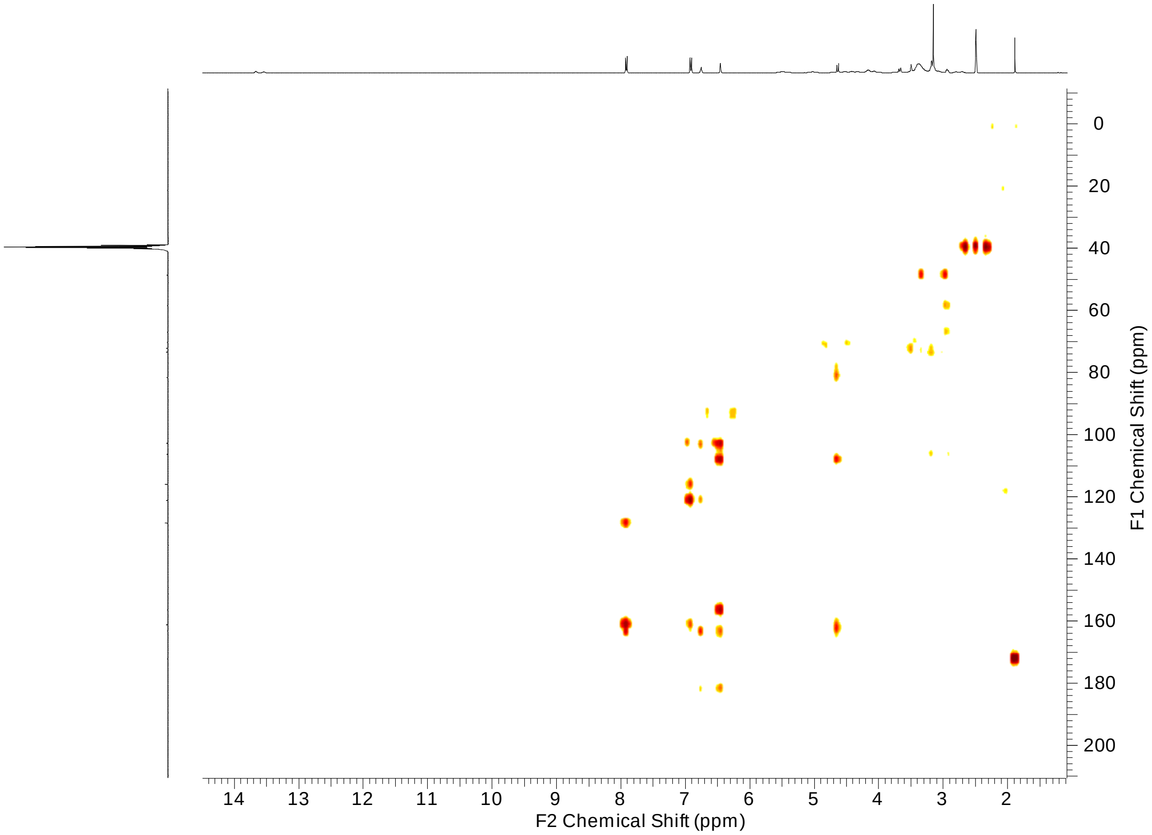

Figure S27. HMBC spectrum of isovitexin-2″-*O*-glucoside (**6a**) recorded in DMSO-d_6.

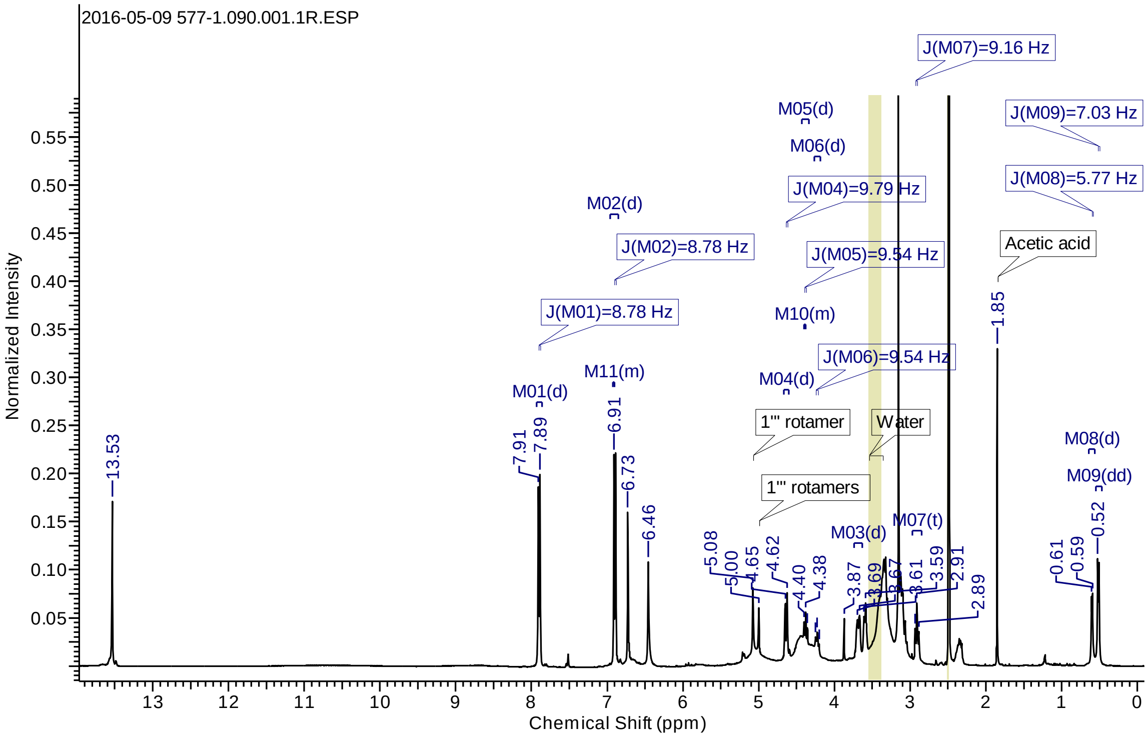

Figure S28. ^{1}H-NMR spectrum of isovitexin-2″-*O*-rhamnoside (**7a**) recorded in DMSO-d_6.

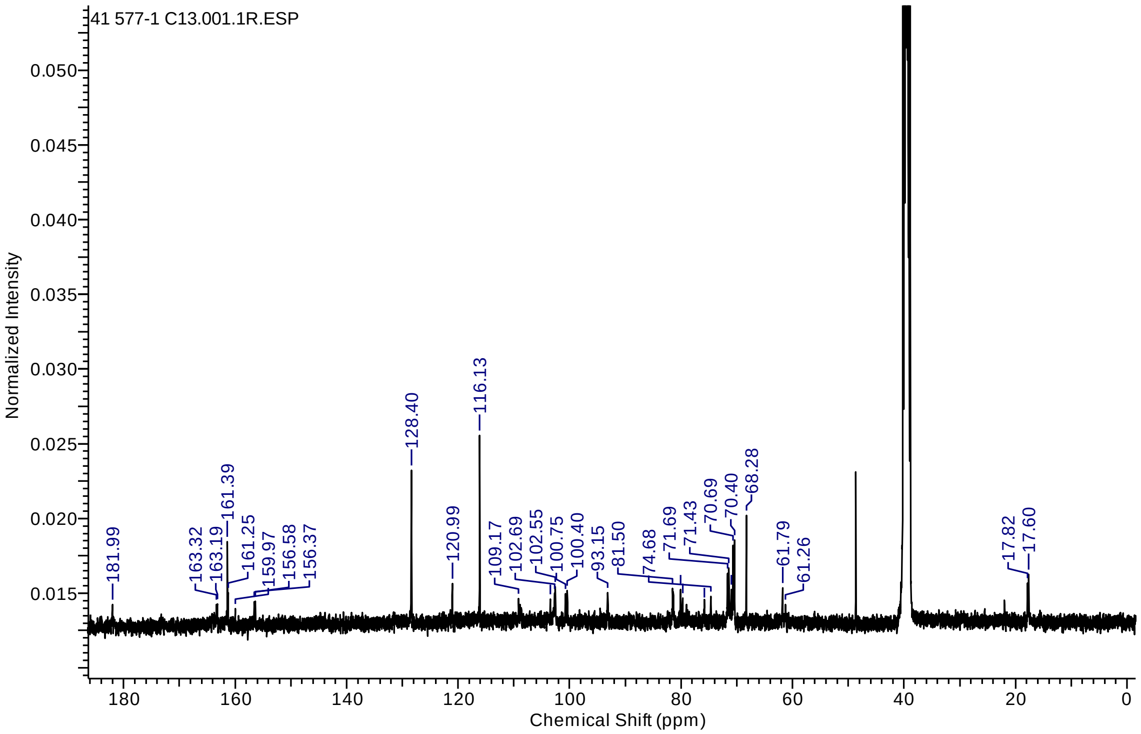

Figure S29. ^{13}C-NMR spectrum of isovitexin-2″-*O*-rhamnoside (**7a**) recorded in DMSO-d_6.

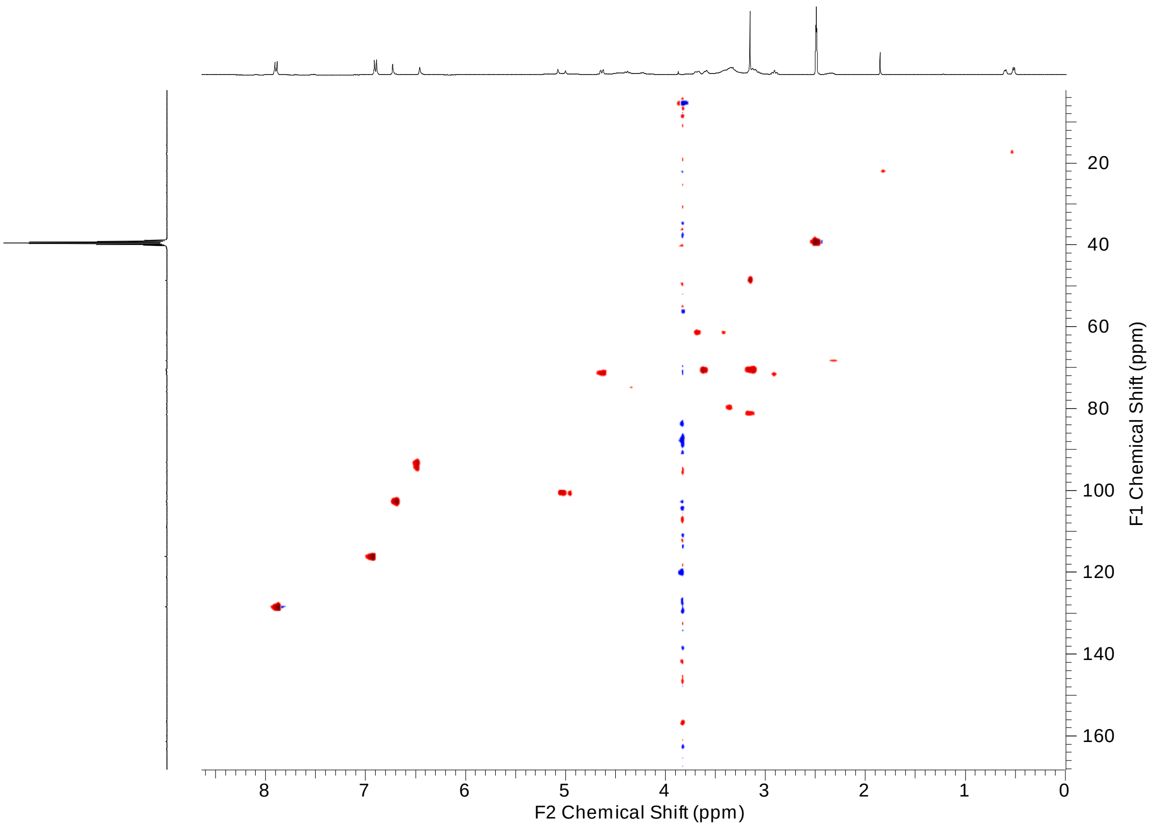

Figure S30. HSQC spectrum of isovitexin-2″-*O*-rhamnoside (**7a**) recorded in DMSO-d_6.

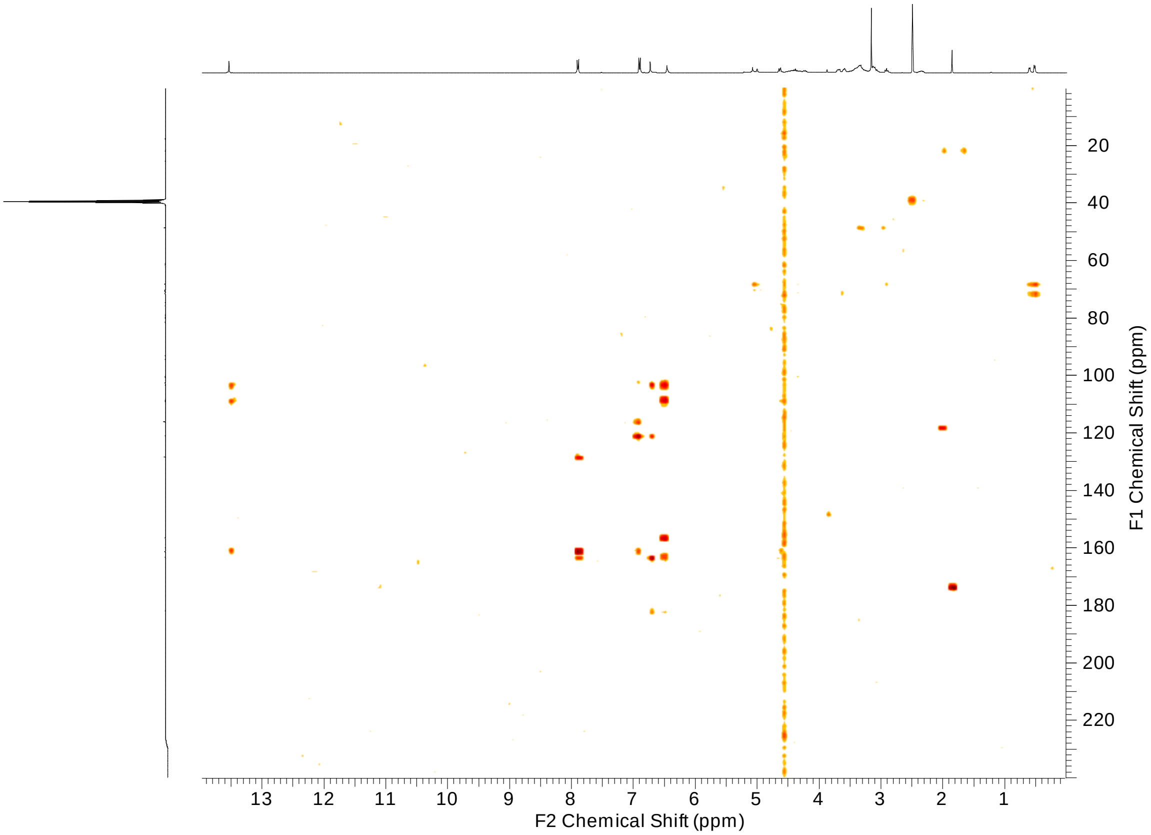

Figure S31. HMBC spectrum of isovitexin-2″-*O*-rhamnoside (**7a**) recorded in DMSO-d_6.

Table S3. Mass spectral data of compounds **1a-7a** identified in *Cecropia obtusifolia*.

No.	Compound	Molecular formula	HESI neg full MS	HESI neg ddMS^2	HESI pos full MS	HESI pos ddMS^2
**1a**	Orientin	$C_{21}H_{20}O_{11}$	447.09336 [M-H]$^-$<br>493.09884 [M-H+FA]$^-$	357.1 [M-H-90(C-Glu)]$^-$ → $^{0,2}X^-$<br>327.1 [M-H-120(C-Glu)]$^-$ → $^{0,3}X^-$	449.10724 [M+H]$^+$	431.1 [M+H-H_2O]$^+$<br>413.1 [M+H-2H_2O]$^+$<br>383.1 [M+H-66(C-Glu)]$^+$ → $^{2,3}X^+$-2H_2O<br>353.1 [M+H-96(C-Glu)]$^+$ → $^{0,4}X^+$-2H_2O<br>329.1 [M+H-120(C-Glu)]$^+$ → $^{0,2}X^+$-2H_2O<br>299.1 [M+H-150(C-Glu)]$^+$ → $^{0,1}X^+$
**2a**	Isoorientin-2"-O-xyloside	$C_{26}H_{28}O_{15}$	579.13597 [M-H]$^-$	459.1 [M-H-120(C-Glu)]$^-$<br>429.1 [M-H-132(O-Xyl)-H_2O]$^-$ → Z_1^-<br>357.1 [M-H-132(O-Xyl)-90(C-Glu)]$^-$ → $^{0,2}X^-$<br>327.1 [M-H-132(O-Xyl)-120(C-Glu)]$^-$ → $^{0,3}X^-$	581.14979 [M+H]$^+$	449.1 [M+H-132(O-Xyl)]$^+$<br>431.14 [M+H-132(O-Xyl)-H_2O]$^+$ → Z_1^+<br>413.1 [M+H-132(O-Xyl)-2H_2O]$^+$ → Z_1^+-H_2O<br>395.1 [M+H-132(O-Xyl)-3H_2O]$^+$ → Z_1^+-2H_2O<br>353.10 [M+H-132(O-Xyl)-96(C-Glu)]$^+$ → $^{0,4}X^+$-2H_2O<br>329.1 [M+H-132(O-Xyl)-120(C-Glu)]$^+$→ $^{0,2}X^+$-2H_2O<br>299.1 [M+H-132(O-Xyl)-150(C-Glu)]$^+$ → $^{0,1}X^+$
**3a**	Isoorientin-4"-O-xyloside	$C_{26}H_{28}O_{15}$	579.13596 [M-H]$^-$	459.1 [M-H-120(C-Glu)]$^-$<br>429.1 [M-H-132(O-Xyl)-H_2O]$^-$ → Z_1^-<br>357.1 [M-H-132(O-Xyl)-90(C-Glu)]$^-$ → $^{0,2}{}_0^-$<br>327.1 [M-H-132(O-Xyl)-120(C-Glu)]$^-$ → $^{0,3}X^-$	581.14975 [M+H]$^+$	449.1 [M+H-132(O-Xyl)]$^+$<br>431.1 [M+H-132(O-Xyl)-H_2O]$^+$ → Z_1^+<br>413.1 [M+H-132(O-Xyl)-2H_2O]$^+$ → Z_1^+-H_2O<br>395.1 [M+H-132(O-Xyl)-3H_2O]$^+$ → Z_1^+-2H_2O<br>353.1 [M+H-132(O-Xyl)-96(C-Glu)]$^+$ → $^{0,4}X^+$-2H_2O<br>329.1 [M+H-132(O-Xyl)-120(C-Glu)]$^+$→ $^{0,2}X^+$-2H_2O<br>299.1 [M+H-132(O-Xyl)-150(C-Glu)]$^+$ → $^{0,1}X^+$
**4a**	Isoorientin-2"-O-rhamnoside	$C_{27}H_{30}O_{15}$	593.15176 [M-H]$^-$	473.1 [M-H-120(C-Glu)]$^-$<br>429.1 [M-H-132(O-Rha)-H_2O]$^-$ → Z_1^-<br>357.1 [M-H-146(O-Rha)-90(C-Glu)]$^-$ → $^{0,2}X^-$<br>327.1 [M-H-146(O-Rha)-120(C-Glu)]$^-$ → $^{0,3}X^-$	595.16505 [M+H]$^+$	449.1 [M+H-146(O-Rha)]$^+$<br>431.1 [M+H-132(O-Rha)-H_2O]$^+$ → Z_1^+<br>413.1 [M+H-132(O-Rha)-2H_2O]$^+$ → Z_1^+-H_2O<br>395.1 [M+H-132(O-Rha)-3H_2O]$^+$ → Z_1^+-2H_2O<br>353.1 [M+H-132(O-Rha)-96(C-Glu)]$^+$ → $^{0,4}X^+$-2H_2O<br>329.1 [M+H-132(O-Rha)-120(C-Glu)]$^+$→ $^{0,2}X^+$-2H_2O<br>299.1 [M+H-132(O-Rha)-150(C-Glu)]$^+$ → $^{0,1}X^+$
**5a**	Isovitexin-2"-O-xyloside	$C_{26}H_{28}O_{14}$	563.14069 [M-H]$^-$	413.1 [M-H-162(O-Xyl)-H_2O]$^-$ → Z_1^-<br>341.1[M-H-162(O-Xyl)-90(C-Glu)]$^-$ → $^{0,2}X^-$<br>311.1 [M-H-162(O-Xyl)-120(C-Glu)]$^-$ → $^{0,3}X^-$<br>293.0 [M-H-162(O-Xyl) -120(C-Glu)-H_2O]$^-$	565.15510 [M+H]$^+$	433.1 [M+H-162(O-Xyl)]$^+$<br>415.1 [M+H-162(O-Xyl)-H_2O]$^+$ → Z_1^+<br>397.1 [M+H-162(O-Xyl)-2H_2O]$^+$ → Z_1^+-H_2O<br>379.1 [M+H-162(O-Xyl)-3H_2O]$^+$ → Z_1^+-2H_2O<br>337.1 [M+H-162(O-Xyl)-96(C-Glu)]$^+$ → $^{0,4}X^+$-2H_2O<br>313.1 [M+H-162(O-Xyl)-120(C-Glu)]$^+$ → $^{0,2}X^+$-2H_2O<br>283.1 [M+H-162(O-Xyl)-150(C-Glu)]$^+$ → $^{0,1}X^+$
**6a**	Isovitexin-2"-O-glucoside	$C_{27}H_{30}O_{15}$	593.15186 [M-H]$^-$	413.1 [M-H-162(O-Glu)-H_2O]$^-$ → Z_1^-<br>341.1 [M-H-162(O-Glu)-90(C-Glu)]$^-$ → $^{0,2}X^-$<br>311.1 [M-H-162(O-Glu)-120(C-Glu)]$^-$ → $^{0,3}X^-$<br>293.0 [M-H-162(O-Glu) -120(C-Glu)-H_2O]$^-$	595.16566 [M+H]$^+$	433.1 [M+H-162(O-Glu)]$^+$<br>415.1 [M+H-162(O-Glu)-H_2O]$^+$ → Z_1^+<br>397.1 [M+H-162(O-Glu)-2H_2O]$^+$ → Z_1^+-H_2O<br>379.1 [M+H-162(O-Glu)-3H_2O]$^+$ → Z_1^+-2H_2O<br>337.1 [M+H-162(O-Glu)-96(C-Glu)]$^+$ → $^{0,4}X^+$-2H_2O<br>313.1 [M+H-162(O-Glu)-120(C-Glu)]$^+$ → $^{0,2}X^+$-2H_2O<br>283.1 [M+H-162(O-Glu)-150(C-Glu)]$^+$ → $^{0,1}X^+$
**7a**	Isovitexin-2"-O-rhamnoside	$C_{27}H_{30}O_{14}$	577.15641 [M-H]$^-$	413.1 [M-H-162(O-Rha)-H_2O]$^-$ → Z_1^-<br>341.1 [M-H-162(O-Rha)-90(C-Glu)]$^-$ → $^{0,2}X^-$<br>311.1 [M-H-162(O-Rha)-120(C-Glu)]$^-$ → $^{0,3}X^-$<br>293.1 [M-H-162(O-Rha) -120(C-Glu)-H_2O]$^-$	579.17070 [M+H]$^+$	433.1 [M+H-162(O-Rha)]$^+$<br>415.1 [M+H-162(O-Rha)-H_2O]$^+$ → Z_1^+<br>397.1 [M+H-162(O-Rha)-2H_2O]$^+$ → Z_1^+-H_2O<br>379.1 [M+H-162(O-Rha)-3H_2O]$^+$ → Z_1^+-2H_2O1<br>337.1 [M+H-162(O-Rha)-96(C-Glu)]$^+$ → $^{0,4}X^+$-2H_2O<br>313.1 [M+H-162(O-Rha)-120(C-Glu)]$^+$ → $^{0,2}X^+$-2H_2O<br>283.1 [M+H-162(O-Rha)-150(C-Glu)]$^+$ → $^{0,1}X^+$

Table S4. ^{1}H NMR spectroscopic data (δ in ppm, J in Hz) for compounds **8a-11a**.

Position	8a	9a	10a	11a
1α	0.90	n.o.	1.30	n.o.
1β	1.94, dd (12.7, 4.4)	1.58	1.57	n.o.
2	3.61, dd (9.79, 4.27)	3.93 dt (12.05, 4.27)	3.88	3.88
3	2.90, d (9.8)	3.32	3.61, d (2.6)	3.61, d (2.6)
5	0.84	1.25	1.55	1.55
6	1.47	1.43	1.36	1.36
7α	1.33	1.31	1.28	1.65
7β	1.54	1.57	1.62	1.77
9	1.71	1.84	1.86	1.91
11α	2.05	2.00	2.01	2.00
11β	2.05	2.00	2.01	2.00
12	5.31[c]	5.31[c]	5.31[c]	5.33[c]
15α	1.20	1.26	1.67	1.67
15β	1.83	1.84	1.77	1.77
16α	2.55	2.62	2.61, dt (13.3, 13.3, 4.0)	2.31, dt (13.3, 13.3, 3.3)
16β	1.60b	1.65	1.71	1.71
18	2.50, br. s	2.50, br. s	2.51, br. s	3.04, br. s
19	-	-	-	3.26, d (3.8)
20	1.35	1.34[b]	1.34	-
21	n.o.	n.o.	1.72	1.67
22α	1.76	1.78	1.76	n.o.
22β	1.62	1.63	1.60	1.28
23	1.00, s	0.98, s	3.53, d (11.0)	3.53, d (11.0)
			3.38, d (10.8)	3.38, d (10.8)
24	0.80, s	0.86, s	0.77, s	0.77, s
25	1.00, s	0.98, s	1.01, s	1.02 s
26	0.77, s	0.76, s	0.77, s	0.74, s
27	1.32, s	1.28, s	1.34, s	1.30, s
29	1.19, s	1.19, s	1.19, s	0.94, s
30	0.92, d (6.53)	0.92, d (6.5)	0.93, d (6.8)	0.93 s
1’	5.31, d (8.0)	5.31, d (8.3)	5.31, d (8.3)	5.33, d (8.0)
2’	3.31	3.32	3.31	3.31
3’	3.33	3.32	3.32	3.32
4’	3.37	3.37	3.37	3.37
5’	3.37	3.37	3.39	3.39
6’A	3.79, dd (12.0, 2.3)	3.79, dd (11.8, 2.0)	3.79, dd (12.0, 4.0)	3.79, dd (12.0, 4.0)
6’B	3.67, dd (12.0, 4.5)	3.67, dd (12.0, 4.5)	3.67, dd (11.9, 4.4)	3.67, dd (11.9, 4.4)

[a] NMR data (δ) were measured in methanol-*d*4. Coupling constants (J) in Hz are given in parentheses.
[b] n.o.: not observed
[c] Overlapped signal with H-1′

Table S5. ^{13}C NMR assignments (δ in ppm) for compounds **8a-11a**.

Position	8a	9a	10a	11a
1	48.28	42.53	42.23	42.07
2	69.54	67.16	67.23	67.24
3	84.55	80.10	78.72	78.77
4	40.51	39.47	42.46	42.46
5	56.71	49.29	44.27	44.12
6	19.69	19.28	19.09	19.13
7	34.08	34.01	33.61	33.26
8	41.29	41.42	41.29	40.93
9	48.72	48.22	48.22	48.85
10	39.20	39.35	39.28	39.09
11	24.67	24.76	24.78	24.78
12	129.51	129.58	129.49	124.79
13	139.71	139.69	139.72	144.43
14	42.70	42.77	42.76	42.72
15	29.64	29.63	29.62	29.42
16	26.51	26.49	26.49	28.40
17	26.36	49.53	49.90	47.07
18	54.96	54.95	54.94	45.04
19	73.62	73.60	73.60	82.39
20	42.95	42.96	42.94	35.96
21	27.22	27.22	27.22	29.44
22	38.31	38.33	38.32	33.38
23	29.31	29.25	71.27	71.27
24	17.45	22.45	17.51	17.58
25	17.14	16.98	17.22	17.34
26	17.59	17.64	17.66	17.85
27	24.79	24.76	25.12	25.09
28	178.52	178.53	178.52	178.54
29	27.06	27.02	27.04	25.12
30	16.59	16.62	16.63	28.64
1’	95.78	95.77	95.75	95.78
2’	73.87	73.83	73.81	73.87
3’	78.60	78.59	78.58	78.58
4’	71.14	71.08	71.01	71.05
5’	78.32	78.29	78.26	78.28
6’	62.44	62.38	62.36	62.33

[a] NMR data (δ) were measured in methanol-*d*4.

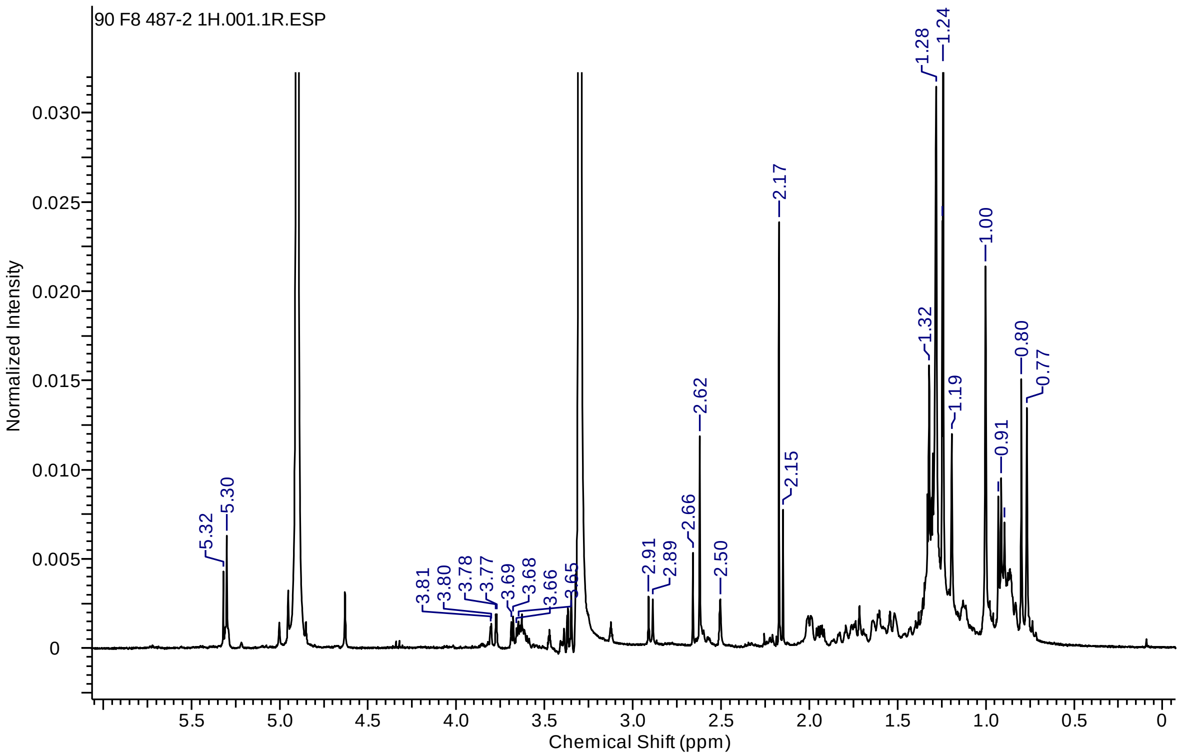

Figure S32. ^{1}H-NMR spectrum of tormentic acid 28-*O*-glucoside (**8a**) recorded in methanol-*d*.

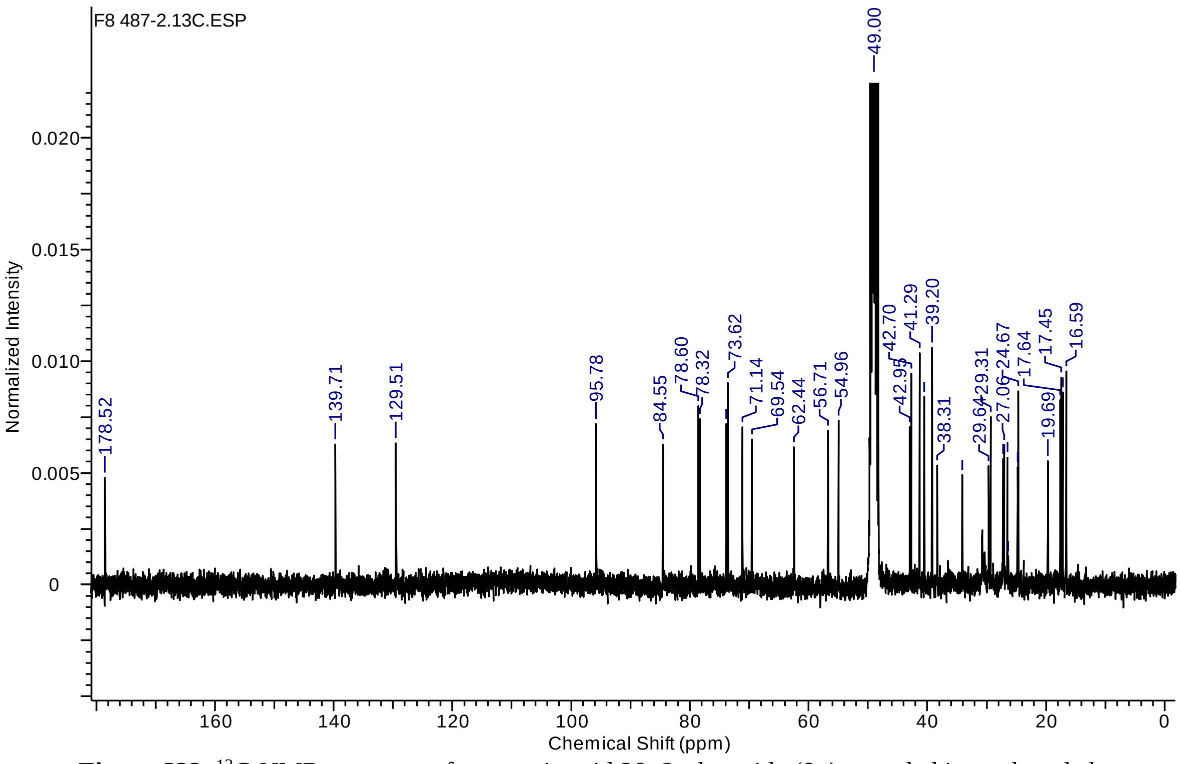

Figure S33. ^{13}C-NMR spectrum of tormentic acid 28-*O*-glucoside (**8a**) recorded in methanol-*d*.

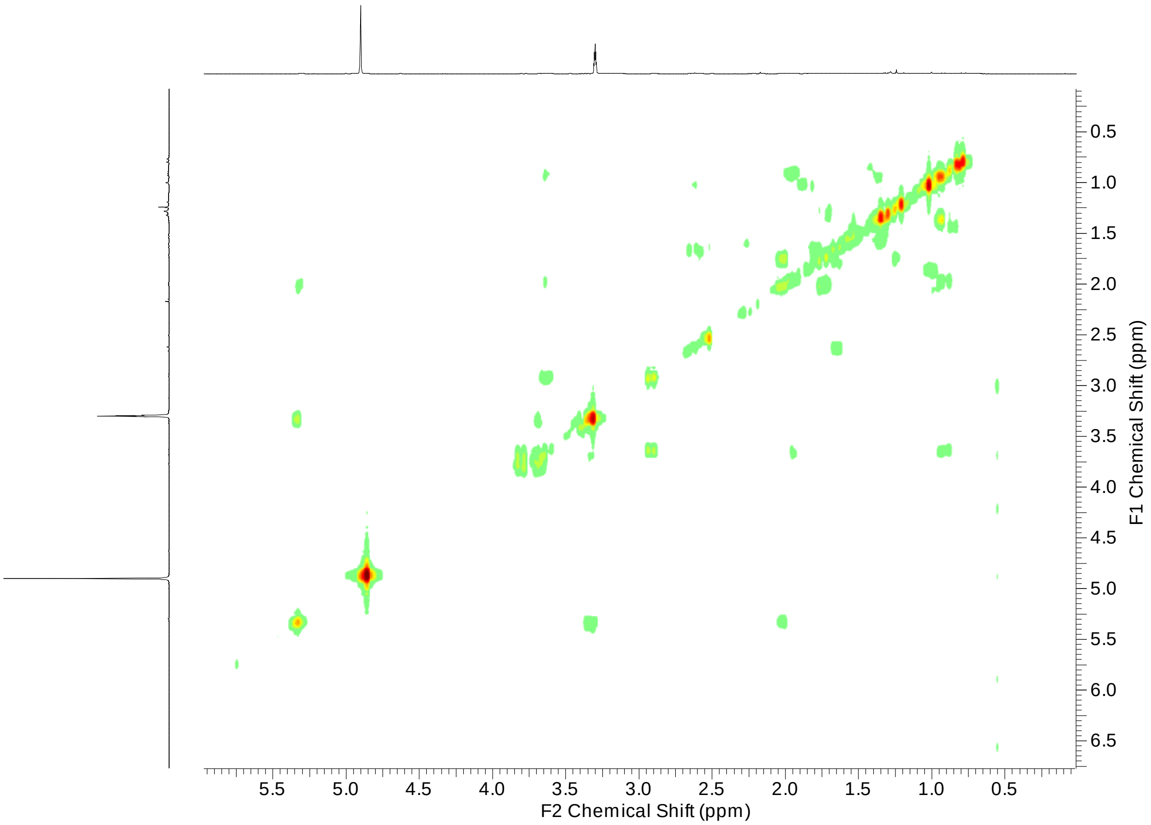

Figure S34. COSY spectrum of tormentic acid 28-*O*-glucoside (**8a**) recorded in methanol-*d*.

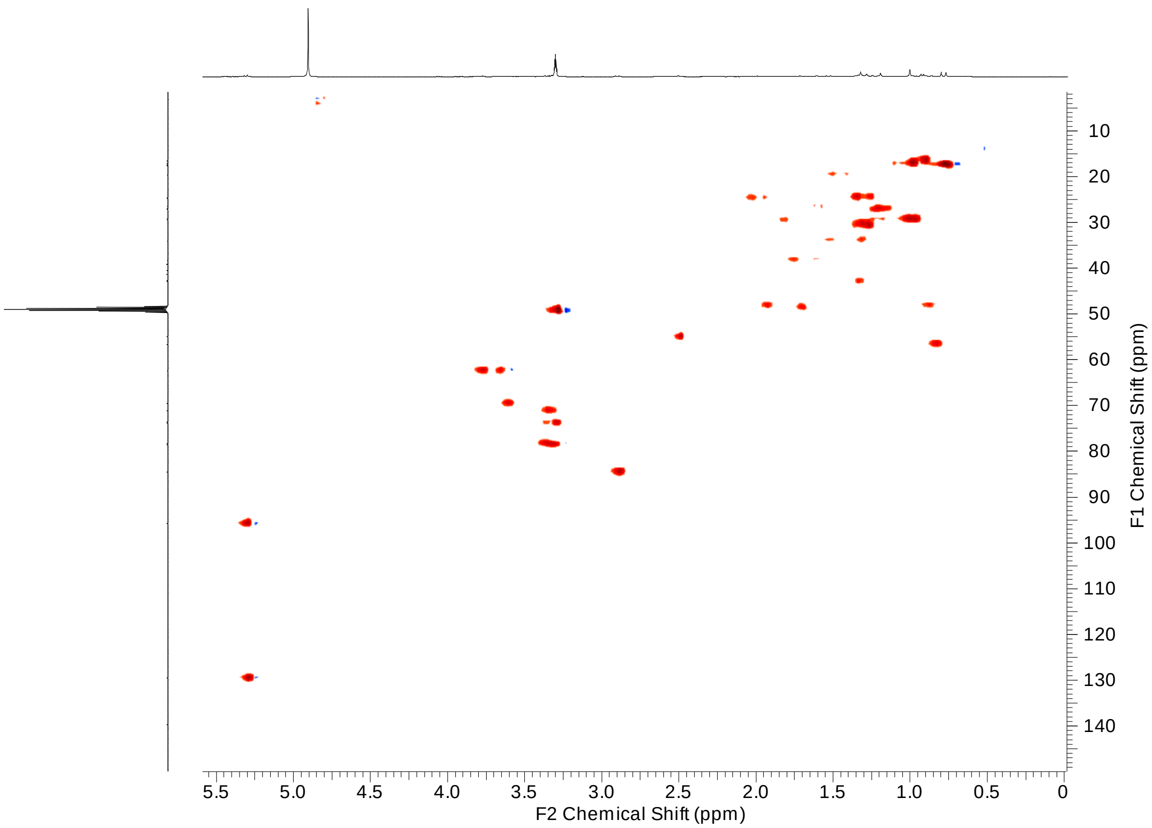

Figure S35. HSQC spectrum of tormentic acid 28-*O*-glucoside (**8a**) recorded in methanol-*d*.

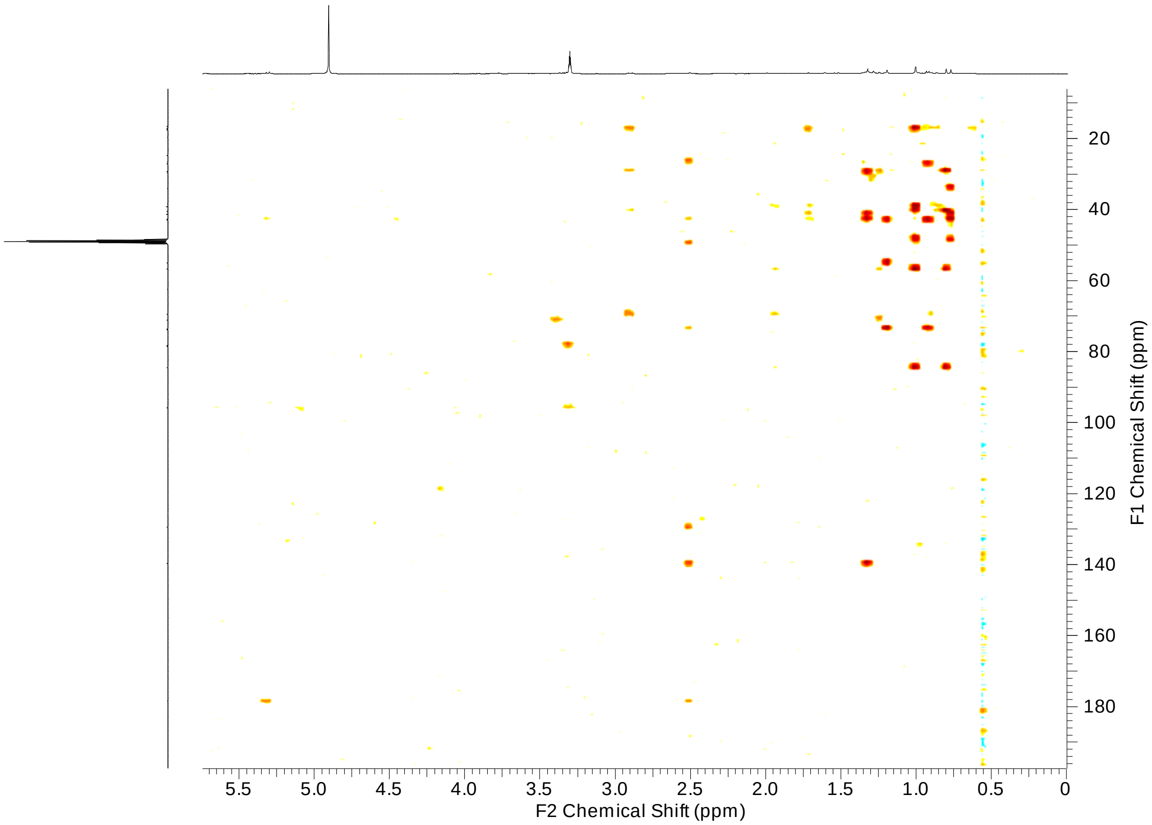

Figure S36. HMBC spectrum of tormentic acid 28-*O*-glucoside (**8a**) recorded in methanol-*d*.

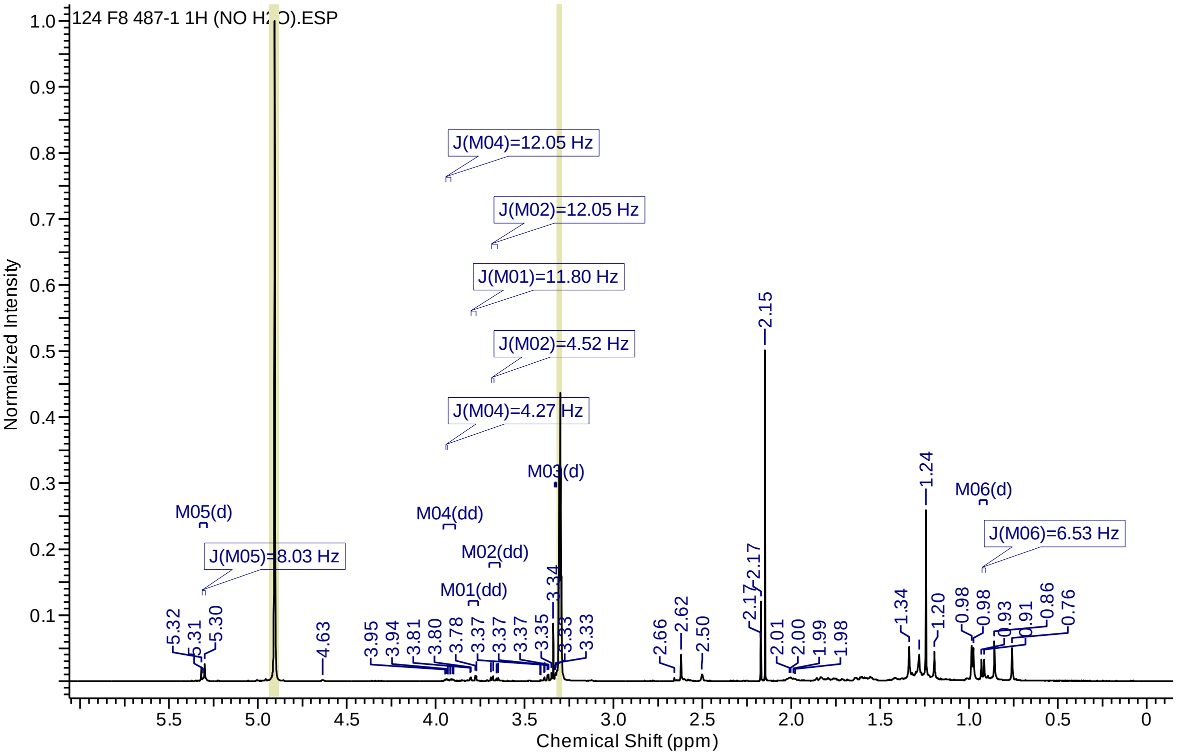

Figure S37. ^{1}H-NMR spectrum of euscaphic acid 28-*O*-glucoside (**9a**) recorded in methanol-*d*.

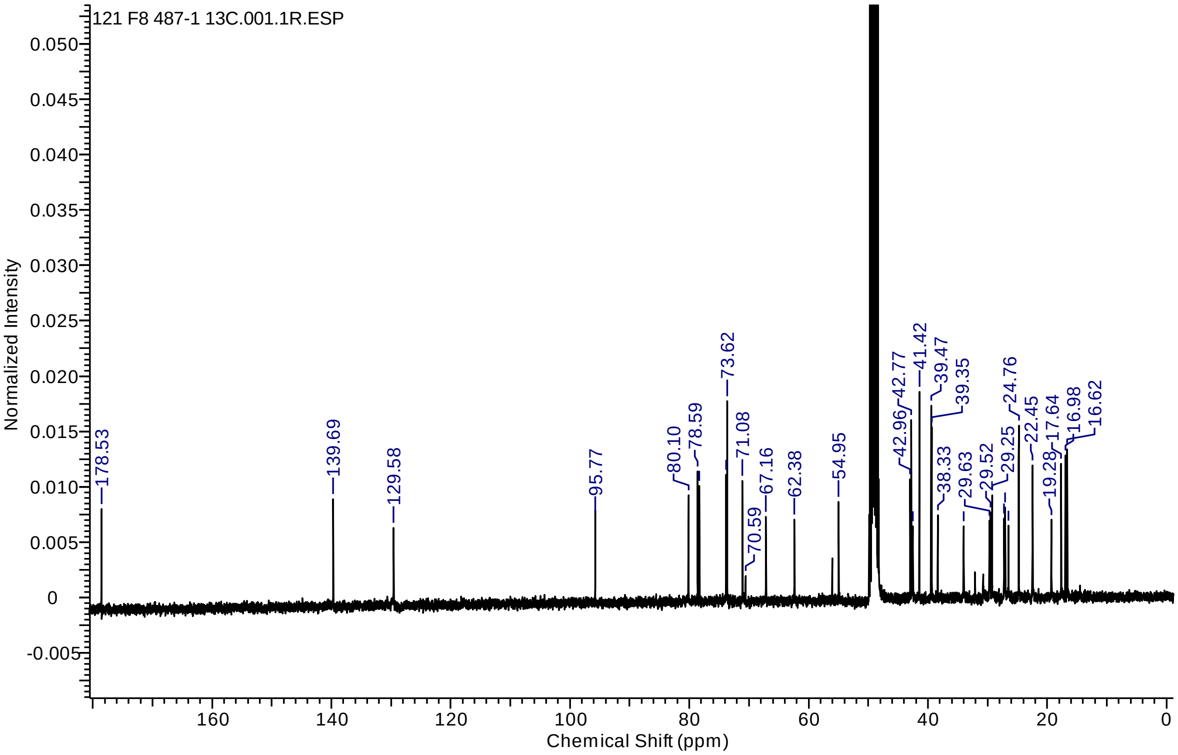

Figure S38. ^{13}C-NMR spectrum of euscaphic acid 28-*O*-glucoside (**9a**) recorded in methanol-*d*.

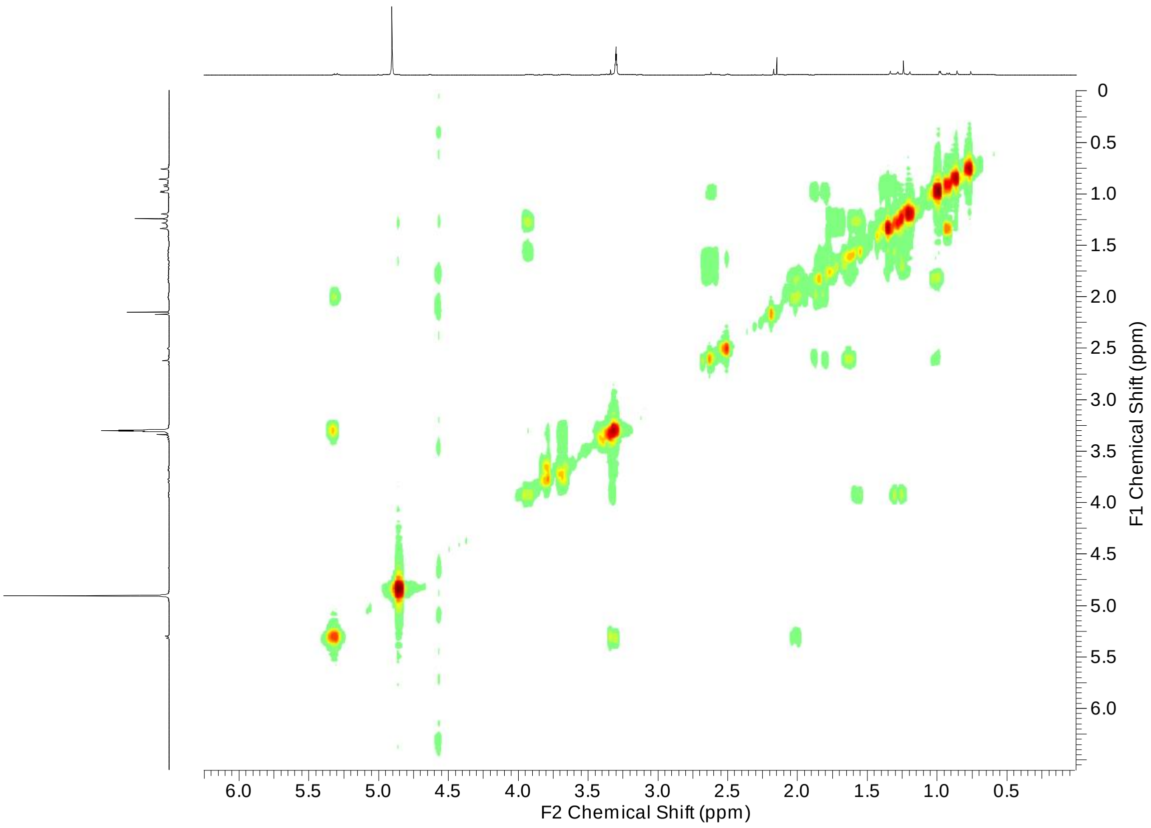

Figure S39. COSY spectrum of euscaphic acid 28-*O*-glucoside (**9a**) recorded in methanol-*d*.

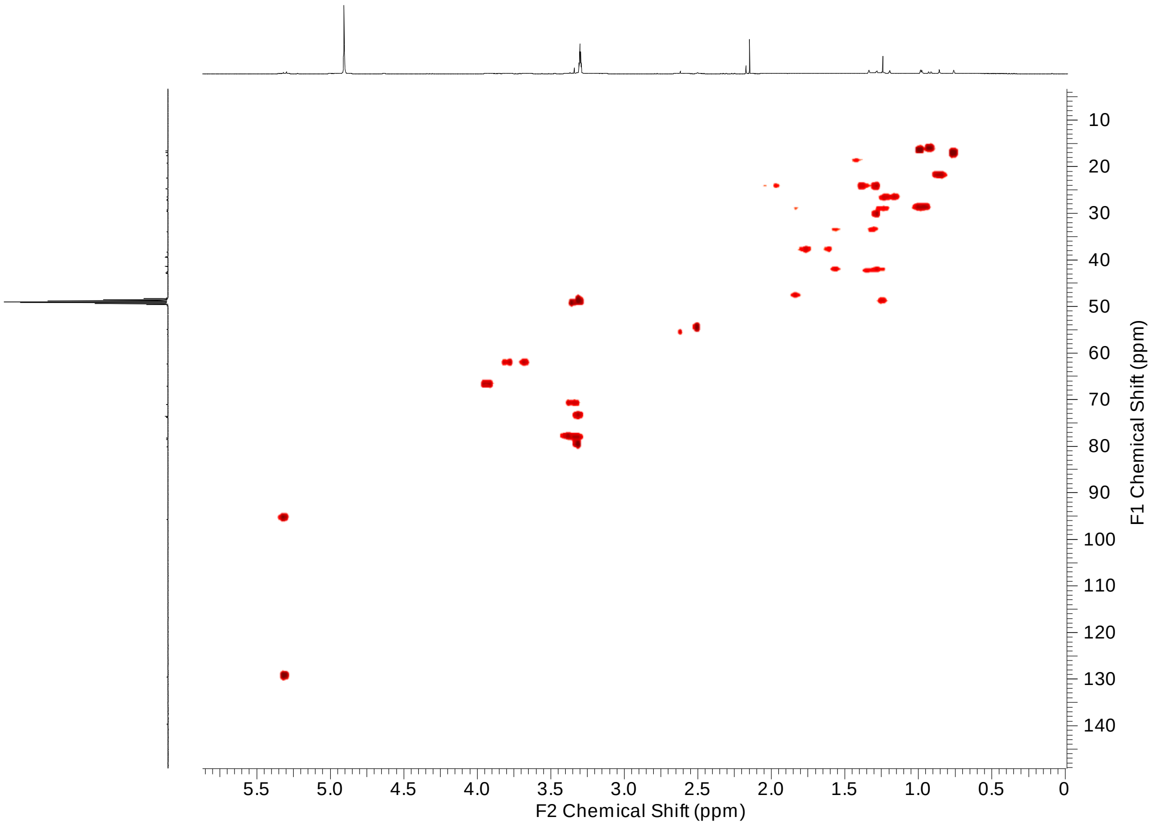

Figure S40. HSQC spectrum of euscaphic acid 28-*O*-glucoside (**9a**) recorded in methanol-*d*.

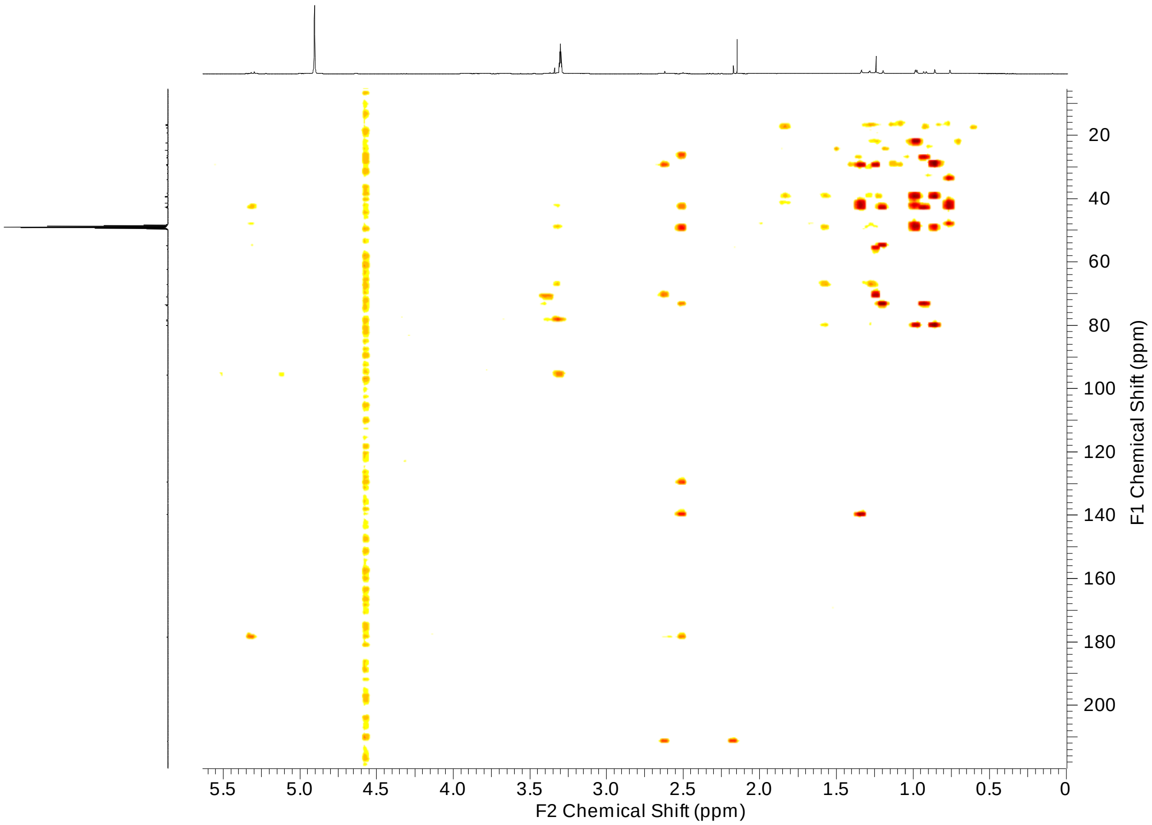

Figure S41. HMBC spectrum of euscaphic acid 28-*O*-glucoside (**9a**) recorded in methanol-*d*.

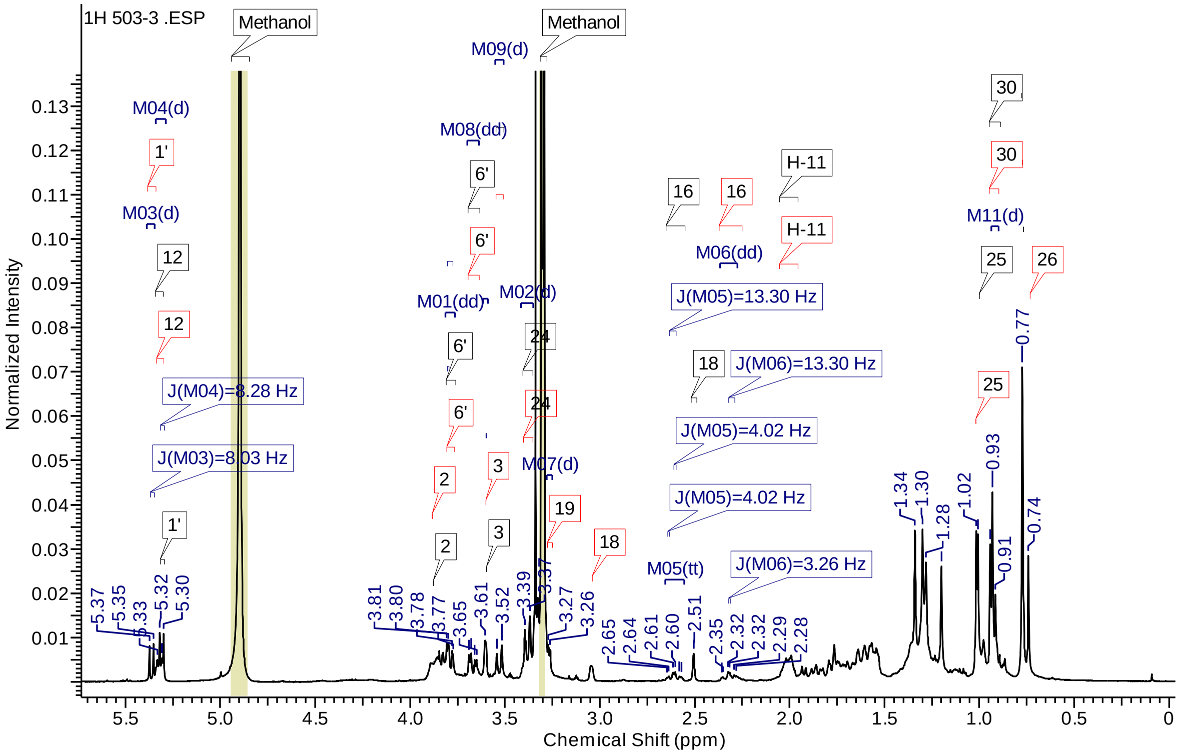

Figure S42. ^{1}H-NMR spectrum of niga-ichigoside F2 (**10a**) and buergericic acid 28-*O*-glucoside (**11a**) recorded in methanol-*d*.

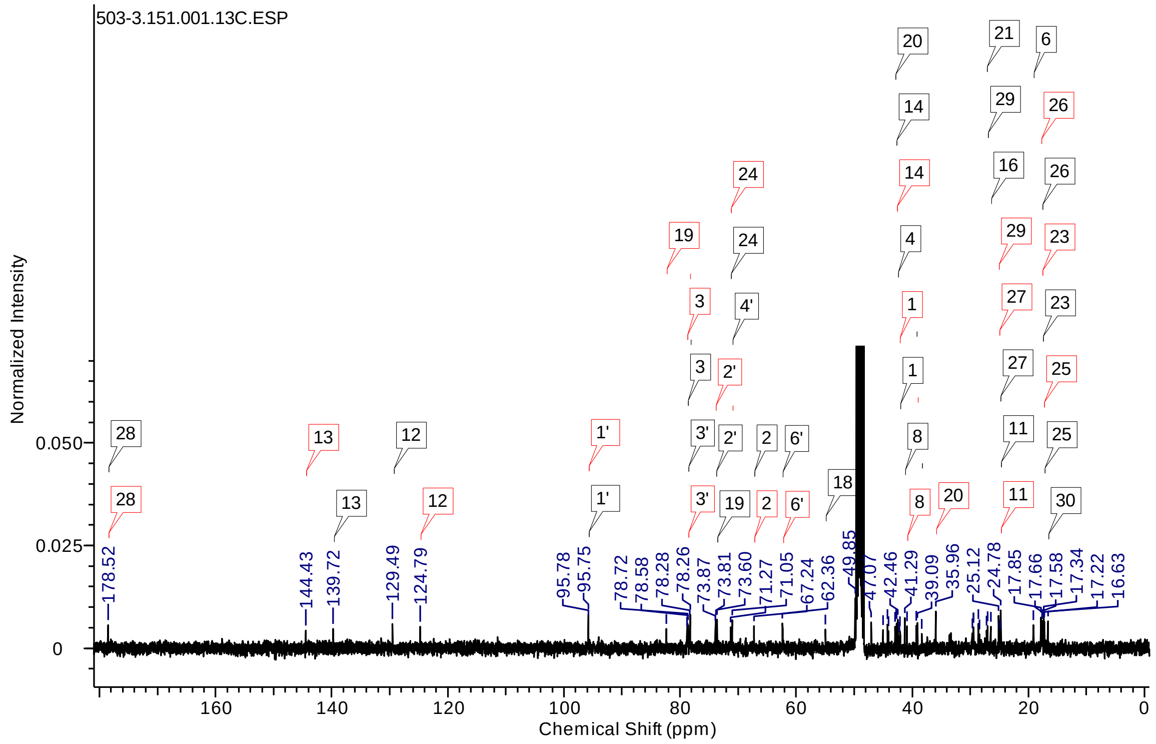

Figure S43. ^{13}C-NMR spectrum of niga-ichigoside F2 (**10a**) and buergericic acid 28-*O*-glucoside (**11a**) recorded in methanol-*d*.

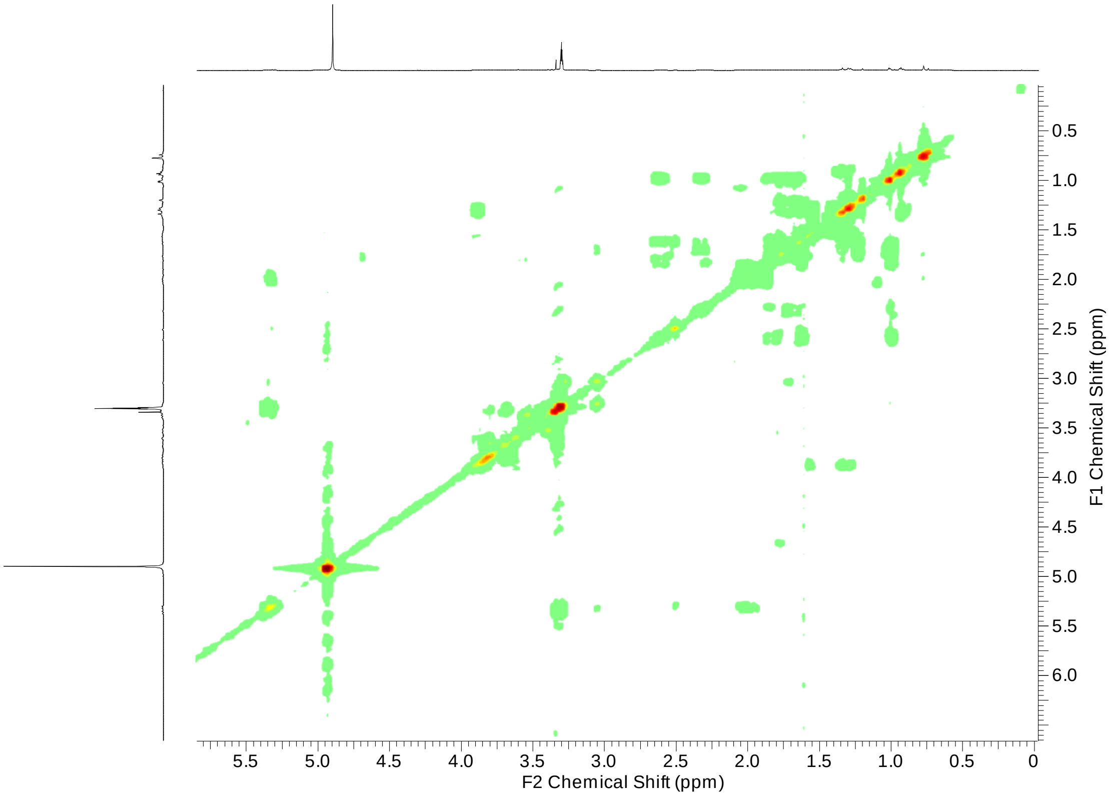

Figure S44. COSY spectrum of niga-ichigoside F2 (**10a**) and buergericic acid 28-*O*-glucoside (**11a**) recorded in methanol-*d*.

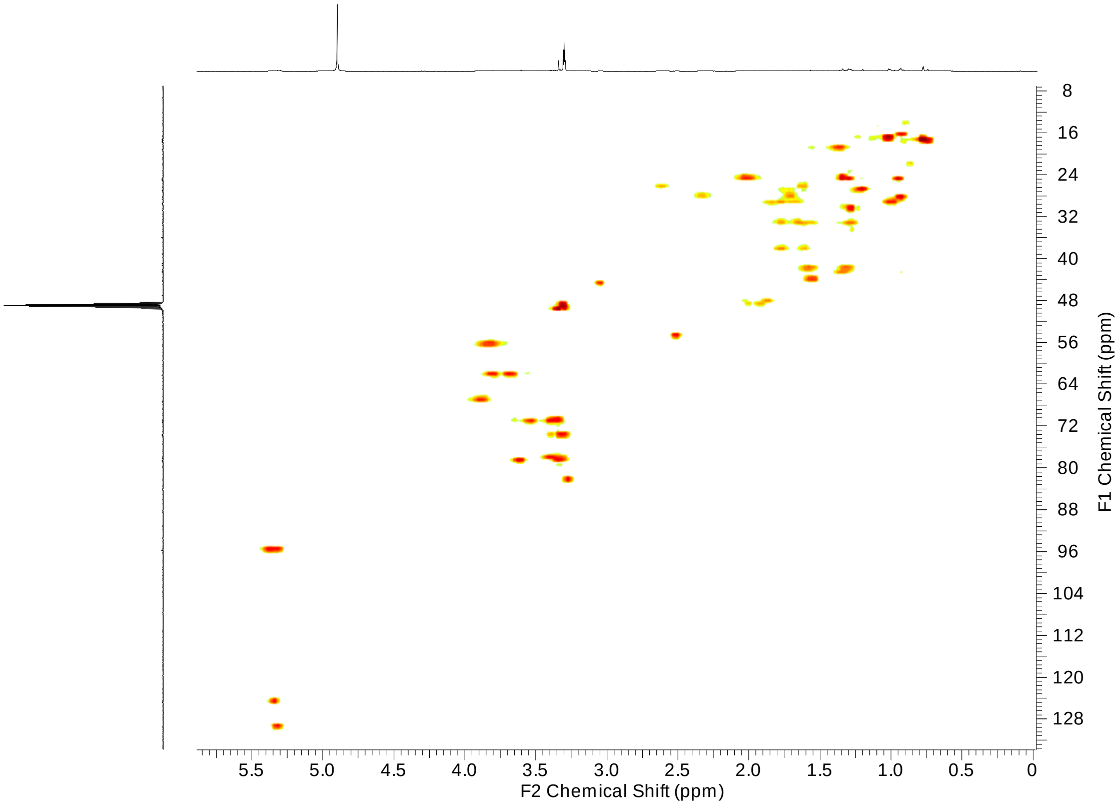

Figure S45. HSQC spectrum of niga-ichigoside F2 (**10a**) and buergericic acid 28-*O*-glucoside (**11a**) recorded in methanol-*d*.

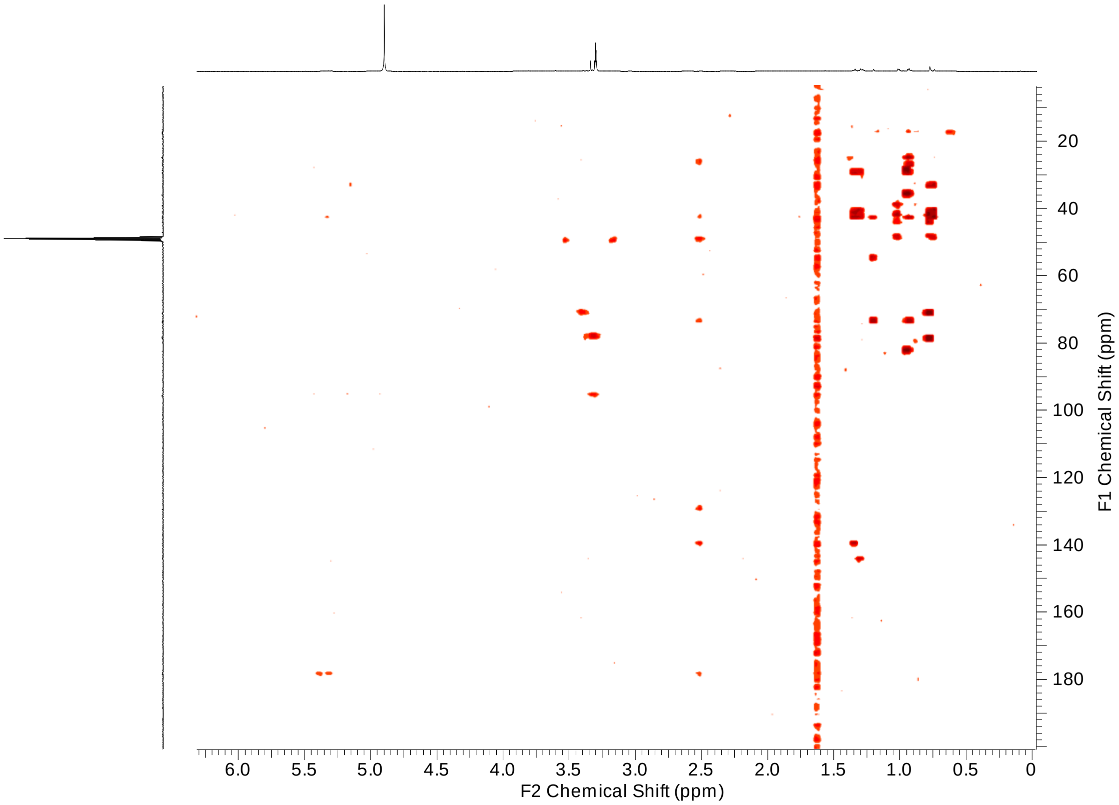

Figure S46. HMBC spectrum of niga-ichigoside F2 (**10a**) and buergericic acid 28-*O*-glucoside (**11a**) recorded in methanol-*d*.

Table S6. Phenolic contents (mg/g dry weight, DW) of leaf extracts from *Cecropia* species. Data represent the mean ± SD (n=4). In each column, different letters mean significant differences ($p<0.05$). TPC: total phenol content; mGAE/g DW, GAE: gallic acid equivalents. TFC: total flavonoid content; mg QE/g DW: quercetin equivalents. O: *C. obtusifolia*; P: *C. peltata*; I: *C. insignis*; and H, *C. hispidissima*.

**Plant**	**TPC (mg GAE/g)**	**TFC (mg RUE/g)**
O1	155.5 ± 7.94^{a}	109.5 ± 9.41^{a}
O2	199.8 ± 8.36^{b}	165.2 ± 19.81^{b}
O3	58.5 ± 2.11^{c}	31.9 ± 3.16^{c}
O4	437.9 ± 23.40^{e}	247.9 ± 18.68^{d}
O5	251.0 ± 9.92^{e}	187.1 ± 16.02^{b}
OC	175.9 ± 5.46^{ab}	128.1 ± 2.44^{a}
P1	129.7 ± 3.51^{f}	105.2 ± 4.33^{ae}
P2	107.2 ± 3.34^{fg}	79.5 ± 8.76^{e}
P3	102.2 ± 4.11^{g}	73.0 ± 9.47^{e}
P4	111.2 ± 6.87^{fg}	80.2 ± 5.03^{e}
PC	31.7 ± 1.39^{h}	14.9 ± 1.23^{c}
I1	160.7 ± 4.96^{a}	114.2 ± 6.18^{af}
I2	160.9 ± 7.20^{a}	98.4 ± 8.50^{ef}
H1	191.8 ± 6.70^{b}	160.2 ± 11.62^{b}
H2	159.5 ± 14.87^{a}	114.0 ± 14.05^{af}

Table S7. Chromatographic and spectral data for the identification of compounds from *Cecropia* species leaves. [a]Tentative identification based on accurate mass. [b]Identification with an analytical standard. [c]Identification by comparison with pure compounds isolated from *C. obtusifolia* (identification number as described in *Isolation and identification of compounds from Cecropia obtusifolia*).*Previously reported in *C. obtusifolia*, *C. peltata*, *C. insignis* and/or *C.hisspidisima*. CFC: caffeic acid, QA: quinic acid, hex: hexose, pen: pentose, deo: deoxyhexose, glu: glucose, xyl: xylose, rha: rhamnose, mal: malonyl.

Peak number	Compound identification	λ_{max} (nm)	Molecular formula	ESI negative full MS	ESI negative mode MS^E	ESI positive full MS	ESI positive mode MS^E	RT (min)
**1**	Chlorogenic acid isomer[a]	216, 326	$C_{16}H_{18}O_9$	353.0880 [M-H]$^-$	191.1 [M-H-162(CFA)]$^-$, 179.0 [M-H-174(QA)]$^-$, 173.1 [M-H-162(CFA)-H_2O]$^-$, 161.0 [M-H-174.0(QA)-H_2O]$^-$	377.0836 [M+Na]$^+$, 355.1030 [M+H]$^+$	163.0 [M+H-174(QA)-H_2O]$^+$, 145.0 [M+H-174(QA)-2H_2O]$^+$, 135.0 [M+H-174(QA)-H_2O-CO]$^+$, 117.0 [M+H-174(QA)-2H_2O-CO]$^+$	9.68
**2**	Chlorogenic acid[b,*]	216, 326	$C_{16}H_{18}O_9$	353.0859 [M-H]$^-$	191.1 [M-H-162(CFA)]$^-$, 179.0 [M-H-174(QA)]$^-$, 173.1 [M-H-162(CFA)-H_2O]$^-$, 161.0 [M-H-174(QA)-H_2O]$^-$	377.0840 [M+Na]$^+$, 355.1023 [M+H]$^+$	163.0 [M+H-174(QA)-H_2O]$^+$, 145.0 [M+H-174(QA)-2H_2O]$^+$, 135.0 [M+H-174(QA)-H_2O-CO]$^+$, 117.0 [M+H-174(QA)-2H_2O-CO]$^+$	15.64
**3**	Luteolin *C*-hexoside-*C*-pentoside[a]	217, 271, 347	$C_{26}H_{28}O_{15}$	579.1353 [M-H]$^-$	519.1 [M-H-60(*C*-pen)]$^-$, 489.1 [M-H-90(*C*-pen)]$^-$, 459.1 [M-H-120(*C*-hex)]$^-$, 429.1 [M-H-150(*C*-hex)]$^-$, 399.1 [M-H-120(*C*-hex)-60(*C*-pen)]$^-$, 369.1 [M-H-150 (*C*-hex)-60(*C*-pen)]$^-$, 341.1 [M-H-134 (*C*-hex)-104 (*C*-pen)]$^-$	603.1313 [M+Na]$^+$, 581.1503 [M+H]$^+$	563.1 [M+H-H_2O]$^+$, 545.1 [M+H-2H_2O]$^+$, 527.1 [M+H-3H_2O]$^+$, 497.1 [M+H-84(*C*-hex)]$^+$, 443.1 [M+H-138(*C*-hex)]$^+$, 425.1 [M+H-156(*C*-hex)]$^+$, 407.1 [M+H-174 (*C*-hex)]$^+$, 395.1 [M+H-120 (*C*-pen)-66(*C*-hex)]$^+$, 365.1 [M+H-120 (*C*-pen)-96(*C*-hex)]$^+$, 353.1 [M+H-90(*C*-pent)-138(*C*-hex)]$^+$, 341.1 [M+H-120(*C*-pen)-120(*C*-hex)]$^+$	23.88
**4**	Luteolin *C*-hexoside-*O*-hexoside[a]	217, 271, 347	$C_{27}H_{30}O_{16}$	609.1461 [M-H]$^-$	489.1 [M-H-120(*C*-hex)]$^-$, 429.1 [M-H-162(*O*-hex)-H_2O]$^-$	633.1425 [M+Na]$^+$, 611.1609 [M+H]$^+$	449.1 [M+H-162 (*O*-hex)]$^+$, 431.1 [M+H-162 (*O*-hex)-H_2O]$^+$, 413.1 [M+H-162 (*O*-hex)-2H_2O]$^+$, 395.1 [M+H-162 (*O*-hex)-3H_2O]$^+$, 383.1 [M+H-162 (*O*-hex)-66 (*C*-hex)]$^+$, 353.1 [M+H-162 (*O*-hex)-96 (*C*-hex)]$^+$, 329.1 [M+H-162 (*O*-hex)-120 (*C*-hex)]$^+$, 299.1 [M+H-162 (*O*-hex)-150 (*C*-hex)]$^+$	25.60
**5**	Isoorientin-2''-*O*-xyloside (**2a**)[c]	217, 271, 348	$C_{26}H_{28}O_{15}$	579.1404 [M-H]$^-$	459.1 [M-H-120 (*C*-glu)]$^-$, 429.1 [M-H-132(*O*-xyl)-H_2O]$^-$, 357.1 [M-H-90(*C*-glu)-132(*O*-xyl)]$^-$, 339.0 [M-H-90(*C*-glu)-132(*O*-xyl)-H_2O]$^-$, 327.0 [M-H-120(*C*-glu)-132(*O*-xyl)]$^-$, 309.0 [M-H-120(*C*-glu)-132(*O*-xyl)-H_2O]$^-$, 298.0 [M-H-120(*C*-glu)-132(*C*-xyl)-CHO]$^-$	603.1328 [M+Na]$^+$, 581.1512 [M+H]$^+$	449.1 [M+H-132 (*O*-xyl)]$^+$, 431.1 [M+H-132 (*O*-xyl)-H_2O]$^+$, 413.1 [M+H-132 (*O*-xyl)-2H_2O]$^+$, 395.1 [M+H-132 (*O*-xyl)-3H_2O]$^+$, 383.1 [M+H-132 (*O*-xyl)-66 (*C*-glu)]$^+$, 353.1 [M+H-132 (*O*-xyl)-96 (*C*-glu)]$^+$, 329.1 [M+H-132 (*O*-xyl)-120 (*C*-glu)]$^+$, 299.1 [M+H-132 (*O*-xyl)-150 (*C*-glu)]$^+$	26.02

**6**	Luteolin *C*-hexoside-*O*-hexoside[a]	217, 271, 348	$C_{27}H_{30}O_{16}$	609.1442 $[M-H]^-$	489.1 [M-H-120 (*C*-hex)]$^-$,<br>429.1 [M-H-162(*O*-hex)-H_2O]$^-$	633.1434 [M+Na]$^+$,<br>611.1617 [M+H]$^+$	449.1066 [M+H-162 (*O*-hex)]$^+$,<br>431.0977 [M+H-162 (*O*-hex)-H_2O]$^+$,<br>413.0869 [M+H-162 (*O*-hex)-2H_2O]$^+$,<br>395.0784 [M+H-162 (*O*-hex)-3H_2O]$^+$,<br>383.0746 [M+H-162 (*O*-hex)-66(*C*-hex)]$^+$,<br>353.0648 [M+H-162 (*O*-hex)-96(*C*-hex)]$^+$,<br>329.0636 [M+H-162 (*O*-hex)-120(*C*-hex)]$^+$,<br>299.0532 [M+H-162 (*O*-hex)-150(*C*-hex)]$^+$	26.40
**7**	Isoorientin-4″-*O*-xyloside (**3a**)[c]	217, 275, 348	$C_{26}H_{28}O_{15}$	579.1354 $[M-H]^-$	459.1 [M-H-120(*C*-glu)]$^-$,<br>429.1 [M-H-132(*O*-pen)-H_2O]$^-$,<br>357.1 [M-H-90(*C*-glu)-132(*O*-xyl)]$^-$,<br>339.0 [M-H-90(*C*-glu)-132(*O*-xyl)-H_2O]$^-$,<br>327.0 [M-H-120(*C*-glu)-132(*O*-xyl)]$^-$,<br>309.0 [M-H-120(*C*-glu)-132(*O*-xyl)-H_2O]$^-$,<br>298.0 [M-H-120(*C*-glu)-132(*C*-xyl)-CHO]$^-$	603.1360 [M+Na]$^+$,<br>581.1511 [M+H]$^+$	449.1 [M+H+H-132 (*O*-xyl)]$^+$,<br>431.1 [M-132 (*O*-xyl)-H_2O]$^+$,<br>413.1 [M+H-132(*O*-xyl)-2H_2O]$^+$,<br>395.1 [M+H-132(*O*-xyl)-3H_2O]$^+$,<br>383.1 [M+H-132(*O*-xyl)-66(*C*-glu)]$^+$,<br>353.1 [M+H-132(*O*-xyl)-96(*C*-glu)]$^+$,<br>329.1 [M+H-132(*O*-xyl)-120(*C*-glu)]$^+$,<br>299.1 [M+H-132(*O*-xyl)-150(*C*-glu)]$^+$	27.39
**8**	Apigenin *C*-hexoside-*C*-pentoside[a]	218, 273, 335	$C_{26}H_{28}O_{14}$	563.1389 $[M-H]^-$	503.1 [M-H-60(*C*-pen)]$^-$,<br>473.1 [M-H-90(*C*-pen)]$^-$,<br>443.1 [M-H-120(*C*-hex)]$^-$,<br>425.1 [M-H-120(*C*-hex)-H_2O]$^-$,<br>383.1 [M-H-120(*C*-hexo)-60(*C*-pen)]$^-$,<br>353.1 [M-H-120(*C*-hex)-90(*C*-pen)]$^-$	565.1580 [M+H]$^+$	511.1 [M+H-3H_2O]$^+$,<br>481.1 [M+H-84(*C*-hex)]$^+$,<br>427.1 [M+H-138(*C*-hex)]$^+$,<br>409.1 [M+H-90(*C*-pen)-66(*C*-hex)]$^+$,<br>391.1 [M+H-90(*C*-pen)-84(*C*-hex)]$^+$	27.80
**9**	Apigenin *C*-hexoside-*C*-pentoside[a]	218, 273, 335	$C_{26}H_{28}O_{14}$	563.1390 $[M-H]^-$	473.1 [M-H-90 (*C*-pen)]$^-$,<br>443.1 [M-H-120(*C*-hex)]$^-$,<br>383.1 [M-H-120(*C*-hex)-60(*C*-pent)]$^-$,<br>353.1 [M-H-120(*C*-hex)-90(*C*-pent)]$^-$	565.1588 [M+H]$^+$	511.1 [M+H-3H_2O]$^+$,<br>481.1 [M+H-84(*C*-hex)]$^+$,<br>427.1 [M+H-138(*C*-hex)]$^+$,<br>409.1 [M+H-90(*C*-pen)-66(*C*-hex)]$^+$,<br>391.1 [M+H-90(*C*-pen)-84(*C*-hex)]$^+$	28.12
**10**	Isoorientin[b,*]	218, 271, 345	$C_{21}H_{20}O_{11}$	447.1055 $[M-H]^-$	429.1 [M-H-H_2O]$^-$,<br>357.1 [M-H-90(*C*-glu)]$^-$,<br>327.0 [M-H-120(*C*-glu)]$^-$	449.1079 [M+H]$^+$	413.1 [M+H-2H_2O]$^+$,<br>395.1 [M+H-3H_2O]$^+$,<br>353.1 [M+H-96(*C*-glu)]$^+$,<br>329.1 [M+H-120(*C*-glu)]$^+$,<br>299.1 [M+H-150(*C*-glu)]$^+$	29.34
**11**	Orientin (**1a**)[b,*]	218, 271, 345	$C_{21}H_{20}O_{11}$	447.1009 $[M-H]^-$	411.0 [M-H-2H_2O]$^-$,<br>357.1 [M-H-90(*C*-glu)]$^-$,<br>327.1 [M-H-120(*C*-glu)]$^-$	449.1070 [M+H]$^+$	413.1 [M+H-2H_2O]$^+$,<br>395.1 [M+H-3H_2O]$^+$,<br>383.1 [M+H-66(*C*-glu)]$^+$,<br>353.1 [M+H-96(*C*-glu)]$^+$,<br>329.1 [M+H-120(*C*-glu)]$^+$,<br>299.1 [M+H-150(*C*-glu)]$^+$	30.15
**12**	Isoorientin-2″-*O*-rhamnoside (**4a**)[c]	217, 271, 345	$C_{27}H_{30}O_{15}$	593.1568 $[M-H]^-$	473.1 [M-H-120(*C*-glu)]$^-$,<br>429.1 [M-H-146(*O*-rha)-H_2O]$^-$,<br>357.1 [M-H-90(*C*-glu)-146(*O*-rha)]$^-$,<br>327.0 [M-H-120(*C*-glu)-146(*O*-rha)]$^-$,<br>309.0 [M-H-120(*C*-glu)-146(*O*-rha)-H_2O]$^-$,<br>298.0 [M-H-120(*C*-glu)-146(*O*-rha)-H_2O-CHO]$^-$	617.1476 [M+Na]$^+$,<br>595.1659 [M+H]$^+$	449.1 [M+H-146 (*O*-rha)]$^+$,<br>431.1 [M+H-146(*O*-rha)-H_2O]$^+$,<br>413.1 [M+H-146(*O*-rha)-2H_2O]$^+$,<br>395.1 [M+H-146(*O*-rha)-3H_2O]$^+$,<br>383.1 [M+H-146(*O*-rha)-66(*C*-hex)]$^+$,<br>353.1 [M+H-146(*O*-rha)-96(*C*-hex)]$^+$,<br>329.1 [M+H-146(*O*-rha)-120(*C*-hex]$^+$,<br>299.1 [M+H-146(*O*-rha)-150(*C*-hex)]$^+$	30.15

**13**	Luteolin *C*-hexoside-*O*-pentoside[a]	218,<br>273,<br>335	$C_{26}H_{28}O_{15}$	579.1343 [M-H]$^-$	459.1 [M-H-120(*C*-hex)]$^-$,<br>447.1 [M-H-132(*O*-pen)],<br>429.1 [M-H-132(*O*-pen)- H_2O],<br>357.1 [M-H-132(*O*-pen)-90(*C*-hex)],<br>327.1 [M-H-132(*O*-pen)-120(*C*-hex)]	603.1346 [M+Na]$^+$,<br>581.1506 [M+H]$^+$	449.1 [M+H-132(*O*-pen)]$^+$,<br>431.1 [M+H-132(*O*-pen)-H_2O]$^+$,<br>413.1 [M+H-132(*O*-pen)-2H_2O]$^+$,<br>395.1 [M+H-132(*O*-pen)-3H_2O]$^+$,<br>383.1 [M+H-132(*O*-pen)-66(*C*-hex)]$^+$,<br>353.1 [M+H-132(*O*-pen)-96(*C*-hex)]$^+$,<br>329.1 [M+H-132(*O*-pen)-120(*C*-hex)]$^+$,<br>299.1 [M+H-132(*O*-pen)-150(*C*-hex)]$^+$	30.20
**14**	Isovitexin-2′′-*O*-glucoside (**6a**)[c]	218,<br>273,<br>335	$C_{27}H_{30}O_{15}$	593.1516 [M-H]$^-$	473.1 [M-120 (*C*-glu),<br>413.1 [M-H-162(*O*-glu) -H_2O]$^-$,<br>341.1 [M-H-162(*O*-glu)-90(*C*-glu)]$^-$,<br>323.1 [M-H-162(*O*-glu)-90(*C*-glu)-H_2O]$^-$,<br>311.1 [M-H-162(*O*-glu)-120(*C*-glu)]$^-$,<br>293.0 [M-H-162(*O*-glu)-120(*C*-glu)-H_2O]$^-$	617.1478 [M+Na]$^+$,<br>595.1662 [M+H]$^+$	433.1 [M+H-162(*O*-glu)]$^+$,<br>415.1 [M-H-162(*O*-glu)-H_2O]$^+$,<br>397.1 [M-H-162(*O*-glu)-2H_2O]$^+$,<br>379.1 [M-H-162(*O*-glu)-3H_2O]$^+$,<br>367.1 [M-H-162(*O*-glu)-66(*C*-glu)]$^+$,<br>337.1 [M-H-162(*O*-glu)-96(*C*-glu)]$^+$,<br>313.1 [M-H-162(*O*-glu)-120(*C*-glu)]$^+$,<br>283.1 [M+H-162(*O*-glu)-150(*C*-glu)]$^+$	33.57
**15**	Isovitexin-2′′-*O*-xyloside (**5a**)[c]	216,<br>273,<br>335	$C_{26}H_{28}O_{14}$	563.1448 [M-H]$^-$	413.1 [M-H-132(*O*-xyl)-H_2O]$^-$,<br>293.4 [M-H-132(*O*-xyl)-120(*C*-glu)-H_2O]$^-$	587.1379 [M+Na]$^+$,<br>565.1562 [M+H]$^+$	433.1 [M+H-132(*O*-xyl)]$^+$,<br>415.1 [M-H-132(*O*-xyl)-H_2O]$^+$,<br>397.1 [M-H-132(*O*-xyl)-2H_2O]$^+$,<br>379.1 [M-H-132(*O*-xyl)-3H_2O]$^+$,<br>367.1 [M-H-132(*O*-xyl)-66(*C*-glu)]$^+$,<br>337.1 [M-H-132(*O*-xyl)-96(*C*-glu)]$^+$,<br>313.1 [M-H-132(*O*-xyl)-120(*C*-glu)]$^+$,<br>283.1 [M+H-132(*O*-xyl)-150(*C*-glu)]$^+$	35.14
**16**	Vitexin[b,*]	216,<br>268,<br>337	$C_{21}H_{20}O_{10}$	431.0956 [M-H]$^-$	413.1 [M-H-H_2O]$^-$,<br>341.1 [M-H-90(*C*-glu)]$^-$,<br>323.1 [M-H-90(*C*-glu)-H_2O]$^-$,<br>311.1 [M-H-120(*C*-glu)]$^-$,<br>293.0 [M-H-120(*C*-glu)-H_2O]$^-$	433.1127 [M+H]$^+$	415.1 [M-H-H_2O]$^+$,<br>397.1 [M-H-2H_2O]$^+$,<br>379.1 [M-H-3H_2O]$^+$,<br>367.1 [M-H-66(*C*-glu)]$^+$,<br>337.1 [M-H-96(*C*-glu)]$^+$,<br>313.1 [M-H-120(*C*-glu)]$^+$,<br>283.1 [M+H-150(*C*-glu)]$^+$	36.82
**17**	Apigenin *C*-hexoside-*O*-pentoside[a]	217,<br>273,<br>335	$C_{26}H_{28}O_{14}$	563.1396 [M-H]$^-$	413.1 [M-H-132(*O*-pen)-H_2O]$^-$,<br>341.1 [M-H-90(*C*-hex)-132(*O*-pen)]$^-$,<br>323.1 [M-H-90(*C*-hex)-132(*O*-pen)-H_2O]$^-$,<br>311.1 [M-H-120 *C*-hex)-132(*O*-pen)]$^-$,<br>293.0 [M-H-120(*C*-hex)-132(*O*-pen)-H_2O]$^-$	565.1554 [M+H]$^+$	433.1 [M+H-132(*O*-pen)]$^+$,<br>415.1 [M-H-132(*O*-pen)-H_2O]$^+$,<br>397.1 [M-H-132(*O*-pen)-2H_2O]$^+$,<br>379.1 [M-H-132(*O*-pen)-3H_2O]$^+$,<br>367.1 [M-H-132(*O*-pen)-66(*C*-hex)]$^+$,<br>337.1 [M-H-132(*O*-pen)-96(*C*-hex)]$^+$,<br>313.1 [M-H-132(*O*-pen)-120(*C*-hex)]$^+$,<br>283.1 [M+H-132(*O*-pen)-150(*C*-hex)]$^+$	36.97
**18**	Diosmetin *C*-hexoside-*O*-pentoside[a]	217,<br>275,<br>345	$C_{27}H_{30}O_{15}$	593.1494 [M-H]$^-$	443.1 [M-H-132(*O*-pen)-H_2O]$^-$,<br>323.1 [M-H-132(*O*-pen)-120(*C*-hex)-H_2O]$^-$	595.1663 [M+H]$^+$	463.1 [M+H-132(*O*-pen)]$^+$,<br>445.1 [M-H-132(*O*-pen)-H_2O]$^+$,<br>427.1 [M-H-132(*O*-pen)-2H_2O]$^+$,<br>409.1 [M-H-132(*O*-pen)-3H_2O]$^+$,<br>397.1 [M-H-132(*O*-pen)-66(*C*-hex)]$^+$,<br>367.1 [M-H-132(*O*-pen)-96(*C*-hex)]$^+$,<br>343.1 [M-H-132(*O*-pen)-120(*C*-hex)]$^+$,<br>313.1 [M+H-132(*O*-pen)-150(*C*-hex)]$^+$	37.40

**19**	Isovitexin 2″-*O*-rhamnoside (**7a**)[c]	217, 273, 335	$C_{27}H_{30}O_{14}$	577.1612 [M-H]$^-$	413.1 [M-H-146(*O*-rha)-H_2O]$^-$,<br>293.0 [M-H-146(*O*-rha)-H_2*O*-120(*C*-glu)]$^-$	601.1538 [M+Na]$^+$,<br>579.1719 [M+H]$^+$	433.1 [M+H-146(*O*-rha)]$^+$,<br>415.1 [M-H-146(*O*-rha)-H_2O]$^+$,<br>397.1 [M-H-146(*O*-rha)-2H_2O]$^+$,<br>379.1 [M-H-146(*O*-rha)-3H_2O]$^+$,<br>367.1 [M-H-146(*O*-rha)-66(*C*-glu)]$^+$,<br>337.1 [M-H-146(*O*-rha)-96(*C*-glu)]$^+$,<br>313.1 [M-H-146(*O*-rha)-120(*C*-glu)]$^+$,<br>283.1 [M+H-146(*O*-rha)-150(*C*-glu)]$^+$	38.36
**20**	Quercetin *O*-hexoside-*O*-deoxyhexoside[a]	214, 253, 338	$C_{27}H_{30}O_{16}$	609.1450 [M-H]$^-$	301.0 [M-H-146(*O*-deo)-162(*O*-hex)]$^-$	633.1439 [M+Na]$^+$,<br>611.1620 [M+H]$^+$	303.1 [M+H-146(*O*-deo)-162(*O*-hex)]$^+$	38.83
**21**	Isovitexin[b,*]	214, 270, 337	$C_{21}H_{20}O_{10}$	431.0859 [M-H]$^-$	341.1 [M-H-90]$^-$,<br>311.1 [M-H-120]$^-$,<br>293.1 [M-H-120(*C*-glu)-H_2O]$^-$	433.1159 [M+H]$^+$	415.1 [M-H-H_2O]$^+$,<br>397.1 [M-H-2H_2O]$^+$,<br>379.1 [M-H-3H_2O]$^+$,<br>367.1 [M-H-66(*C*-glu)]$^+$,<br>337.1 [M-H-96(*C*-glu)]$^+$,<br>313.1 [M-H-120(*C*-glu)]$^+$,<br>283.1 [M+H-150(*C*-glu)]$^+$	38.66
**22**	Apigenin *C*-hexoside-*O*-pentoside[a]	217, 273, 335	$C_{26}H_{28}O_{14}$	563.1462 [M-H]$^-$	443.1 [M-H-120(*C*-hex)]$^-$,<br>413.1 [M-H-132 (*O*-pen)-H_2O]$^-$,<br>341.1 [M-H-90(*C*-hex)-132(*O*-pen)]$^-$,<br>311.1 [M-H-120(*C*-hex)-132(*O*-pen)]$^-$,<br>293.1 [M-H-120(*C*-hex)-132(*O*-pen)-H_2O]$^-$	565.1561 [M+H]$^+$	433.1 [M+H-132(*O*-pen)]$^+$,<br>415.1 [M-H-132(*O*-pen)-H_2O]$^+$,<br>397.1 [M-H-132(*O*-pen)-2H_2O]$^+$,<br>379.1 [M-H-132(*O*-pen)-3H_2O]$^+$,<br>367.1 [M-H-132(*O*-pen)-66(*C*-hex)]$^+$,<br>337.1 [M-H-132(*O*-pen)-96(*C*-hex)]$^+$,<br>313.1 [M-H-132(*O*-pen)-120(*C*-hex)]$^+$,<br>283.1 [M+H-132(*O*-pen)-150(*C*-hex)]$^+$	38.66
**23**	Rutin[b,*]	217, 253, 354	$C_{27}H_{30}O_{16}$	609.1448 [M-H]$^-$	301.0 [M-H-146(*O*-rha)-162(*O*-glu)]$^-$	633.1486 [M+Na]$^+$,<br>611.1620 [M+H]$^+$	303.0 [M+H-146(*O*-rha)-162(*O*-glu)]$^+$	39.24
**24**	Diosmetin-*C*-hexoside-*O*-deoxyhexoside[a]	217, 278, 335	$C_{28}H_{32}O_{15}$	607.1664 [M-H]$^-$	443.1 [M-H-146(*O*-deo)-H_2O]$^-$,<br>323.1 [M-H-146(*O*-deo)-120(*C*-hex)-H_2O]$^-$	631.1642 [M+Na]$^+$,<br>609.1821 [M+H]$^+$	463.1 [M+H-146(*O*-deo)]$^+$,<br>445.1 [M-H-146(*O*-deo)-H_2O]$^+$,<br>427.1 [M+H-146(*O*-deo)-2H_2O]$^+$,<br>409.1 [M+H-146(*O*-deo)-3H_2O]$^+$,<br>397.1 [M+H-146(*O*-deo)-66(*C*-hex)]$^+$,<br>367.1 [M+H-146(*O*-deo)-96(*C*-hex)]$^+$,<br>343.1 [M+H-146(*O*-deo)-120(*C*-hex)]$^+$,<br>313.1 [M+H-146(*O*-deo)-150(*C*-hex)]$^+$	39.51
**25**	Diosmetin-*C*-hexoside[a]	216, 278, 335	$C_{22}H_{22}O_{11}$	461.1155 [M-H]$^-$	371.1 [M-H-90(*C*-hex)]$^-$,<br>341.1 [M-H-120(*C*-hex)]$^-$	463.1231 [M+H]$^+$	427.1 [M+H-2H_2O]$^+$,<br>409.1 [M+H-3H_2O]$^+$,<br>367.1 [M+H-96(*C*-hex)]$^+$,<br>343.1 [M+H-120(*C*-hex)]$^+$,<br>313.1 [M+H-150(*C*-hex)]$^+$	39.95
**26**	Luteolin-*O*-malonyl-*C*-hexoside[a]	216, 278, 350	$C_{24}H_{22}O_{14}$	533.0961 [M-H]$^-$,<br>489.1027 [M-H-44 (COO)]$^-$	489.1 [M-H-44(COO)]$^-$,<br>357.1 [M-H-86(*O*-mal:$C_3H_2O_3$)-90(*C*-hex)]$^-$,<br>327.1 [M-H-86(*O*-mal:$C_3H_2O_3$)-120(*C*-hex)]$^-$	535.1088 [M+H]$^+$	395.1 [M+H-86(*O*-mal)-3H_2O]$^+$,<br>377.1 [M+H-86(*O*-mal)-4H_2O]$^+$,<br>353.1 [M+H-86(*O*-mal)-66(*C*-hex)]$^+$,<br>329.1 [M+H-86(*O*-mal)-120(*C*-hex)]$^+$,	39.97

							299.1 [M+H-86(*O*-mal)-150(*C*-hex)]$^{+}$	
**27**	Quercetin-*O*-hexoside[a,*]	215, 253, 354	$C_{21}H_{20}O_{12}$	463.0869 [M-H]$^{-}$	300.0 [M-2H-162(*O*-glu)]$^{-}$	487.0861 [M+Na]$^{+}$, 465.1031 [M+H]$^{+}$	303.1 [M+H-162(*O*-hex)]$^{+}$	40.50
**28**	Quercetin-*O*-hexoside[a,*]	215, 253, 354	$C_{21}H_{20}O_{12}$	463.0868 [M-H]$^{-}$	300.0 [M-2H-162(*O*-glu)]$^{-}$	487.0937 [M+Na]$^{+}$, 465.1094 [M+H]$^{+}$	303.1 [M+H-162(*O*-hex)]$^{+}$	41.00
**29**	Luteolin-*O*-malonyl-*C*-hexoside[a]	217, 275, 347	$C_{24}H_{22}O_{14}$	533.0937 [M-H]$^{-}$, 489.1045 [M-H-44 (COO)]$^{-}$	489.1 [M-H-44(COO)]$^{-}$, 357.1 [M-H-86(*O*-mal)-90 (*C*-hex)]$^{-}$, 327.1 [M-H-86(*O*-mal)-120(*C*-hex)]$^{-}$	535.1078 [M+H]$^{+}$	499.1 [M-H-2H$_2$O]$^{+}$, 395.1 [M+H-86 (*O*-mal)-3H$_2$O]$^{+}$, 377.1 [M+H-86 (*O*-mal)-4H$_2$O]$^{+}$, 353.1 [M+H-86(*O*-mal)-66(*C*-hex)]$^{+}$, 329.1 [M+H-86(*O*-mal)-120(*C*-hex)]$^{+}$, 299.1 [M+H-86(*O*-mal)-150(*C*-hex)]$^{+}$	42.34
**30**	Quercetin *O*-pentoside[a]	216, 253, 352	$C_{20}H_{18}O_{11}$	433.0763 [M-H]$^{-}$	300.0 [M-2H-132(*O*-pen)]$^{-}$	457.0740 [M+Na]$^{+}$, 435.0920 [M+H]$^{+}$	303.1 [M+H-132(*O*-pen)]$^{+}$	43.23
**31**	Apigenin-*O*-malonyl-*C*-hexoside[a]	218, 278, 335	$C_{24}H_{22}O_{13}$	517.0990 [M-H]$^{-}$, 473.1108 [M-H-44 (COO)]$^{-}$	473.1 [M-H-44(COO)]$^{-}$, 341.1 [M-H-86(*O*-mal)-90(*C*-hex)]$^{-}$, 311.1 [M-H-86(*O*-mal)-120(*C*-hex)]$^{-}$	519.1135 [M+H]$^{+}$	483.1 [M-H-2H$_2$O]$^{+}$, 397.1 [M+H-86(*O*-mal)-2H$_2$O]$^{+}$, 379.1 [M+H-86(*O*-mal)-3H$_2$O]$^{+}$, 337.1 [M+H-86(*O*-mal)-96(*C*-hex)]$^{+}$, 313.1 [M+H-86(*O*-mal)-120(*C*-hex)]$^{+}$, 283.1 [M+H-86(*O*-mal)-150(*C*-hex)]$^{+}$	42.72
**32**	Apigenin-*O*-deoxyhexoside-*O*-malonyl-*C*-hexoside[a]	218, 275, 335	$C_{30}H_{32}O_{17}$	663.1522 [M-H]$^{-}$, 619.1648 [M-H-44 (COO)]$^{-}$	619.2 [M-H-44 (COO)]$^{-}$, 559.1 [M-H-86(*O*-mal)-H$_2$O]$^{-}$, 473.1 [M-44(COO)-146(*O*-deo)]$^{-}$, 455.1 [M-44(COO)-146(*O*-deo)-H$_2$O]$^{-}$, 413.1 [M-H-146(*O*-deo)-86(*O*-mal)-H$_2$O]$^{-}$, 395.0 [M-H-146(*O*-deo)-86(*O*-mal)-2H$_2$O]$^{-}$, 341.1 [M-H-146(*O*-deo)-86(*O*-mal)-90(*C*-hex)]$^{-}$, 323.1 [M-H-146(*O*-deo)-86(*O*-mal)-90(*C*-hex)-H$_2$O]$^{-}$, 311.1 [M-H-146(*O*-deo)-86(*O*-mal)-120(*C*-hex)]$^{-}$, 293.1 [M-H-146(*O*-deo)-86(*O*-mal)-120(*C*-hex)-H$_2$O]$^{-}$	687.1528 [M+Na)]$^{+}$, 665.1712 [M+H]$^{+}$	519.1 [M+H-146(*O*-deo)]$^{+}$, 501.1 [M-H-146(*O*-deo)-H$_2$O]$^{+}$, 483.1 [M+H-146(*O*-deo)-2H$_2$O]$^{+}$, 379.1 [M+H-146(*O*-deo)-86(*O*-mal)-3H$_2$O]$^{+}$, 337.1 [M+H-146(*O*-deo)-86(*O*-mal)-96(*C*-hex)]$^{+}$, 313.1 [M+H-146(*O*-deo)-86(*O*-mal)-120(*C*-hex)]$^{+}$, 283.1 [M+H-146(*O*-deo)-86(*O*-mal)-150(*C*-hex)]$^{+}$	43.70
**33**	Quercetin *O*-pentoside[a]	218, 253, 353	$C_{20}H_{18}O_{11}$	433.0794 [M-H]$^{-}$	300.0 [M-2H-132(*O*-pen)]$^{-}$	457.0742 [M+Na]$^{+}$, 435.0925 [M+H]$^{+}$	303.1 [M+H-132(*O*-pen)]$^{+}$	43.87
**34**	Apigenin-*O*-malonyl-*C*-hexoside[a]	218, 270, 338	$C_{24}H_{22}O_{13}$	517.1000 [M-H]$^{-}$, 473.1093 [M-H-44 (COO)]$^{-}$	473.1 [M-H-44(COO)]$^{-}$, 341.1 [M-H-86(*O*-mal) -90(*C*-hex)]$^{-}$, 311.1 [M-H-86(*O*-mal)-120(*C*-hex)]$^{-}$	519.1126 [M+H]$^{+}$	483.1 [M+H-2H$_2$O]$^{+}$, 397.1 [M+H-86 (*O*-mal)-2H$_2$O]$^{+}$, 379.1 [M+H-86 (*O*-mal)-3H$_2$O]$^{+}$, 337.1 [M+H-86(*O*-mal)-96(*C*-hex)]$^{+}$, 313.1 [M+H-86(*O*-mal)-120(*C*-hex)]$^{+}$, 283.1 [M+H-86(*O*-mal)-150(*C*-hex)]$^{+}$	44.80

**35**	Niga-ichigoside F2 (**10a**)[c]	-	$C_{36}H_{58}O_{11}$	711.3909 [M-H+HCOOH]$^-$	503.3 [M-H-162(*O*-glu)]$^-$	689.3870 [M+Na]$^+$	-	46.79
**36**	Buergericic acid 28-*O*-glucoside (**11a**)[c]	-	$C_{36}H_{58}O_{11}$	711.3909 [M-H+HCOOH]$^-$	503.3 [M-H-162(*O*-glu)]$^-$	689.3870 [M+Na]$^+$	-	46.79
**37**	Quercetin *O*-hexoside-*O*-hexoside[a]	219, 253, 340	$C_{27}H_{30}O_{17}$	625.1205 [M-H]$^-$	463.1 [M-H-162(*O*-hex)]$^-$, 301.0 [M-H-162(*O*-hex)-162(*O*-hex)]$^-$	649.1179 [M+Na]$^+$, 627.1353 [M+H]$^+$	301.1 [M+H-162(*O*-hex)-162(*O*-hex)]$^+$	47.68
**38**	Triterpenoid saponin-*O*-hexoside 1[a]	-	$C_{36}H_{58}O_{11}$	711.3911 [M-H+HCOOH]$^-$	503.3 [M-H-162(*O*-hex)]$^-$	689.3864 [M+Na]$^+$	-	49.09
**39**	Triterpenoid saponin-*O*-hexoside 2[a]	-	$C_{36}H_{58}O_{11}$	711.3903 [M-H+HCOOH]$^-$	503.3 [M-H-162(*O*-hex)]$^-$	689.3829 [M+Na]$^+$	-	50.25
**40**	Triterpenoid saponin-*O*-hexoside 3[a]	-	$C_{36}H_{58}O_{11}$	711.3918 [M-H+HCOOH]$^-$	503.3 [M-H-162(*O*-hex)]$^-$	689.3830 [M+Na]$^+$	-	53.04
**41**	(+)-Vaccinin A (**13a**)[c]	220, 280, 315, 393	$C_{24}H_{16}O_9$	447.0713 [M-H]$^-$	337.0 [M-H-$C_6H_6O_2$]$^-$, 325.0 [M-H-$C_7H_6O_2$]$^-$, 323.0 [M-H-$C_7H_8O_2$]$^-$, 295.0 [M-H-$C_8H_8O_3$]$^-$, 283.0 [M-H-$C_9H_8O_3$]$^-$,	449.0891 [M+H]$^+$	327.0 [M+H-$C_7H_6O_2$]$^+$, 297.0 [M+H-$C_8H_8O_3$]$^+$	53.64
**42**	(+)-Mururin A (**12a**)[c]	220, 280, 315, 393	$C_{24}H_{16}O_9$	447.0714 [M-H]$^-$	429.1 [M-H-H_2O]$^-$, 403.1 [M-H-C_2H_4O]$^-$, 337.0 [M-H-$C_6H_6O_2$]$^-$, 325.0 [M-H-$C_7H_6O_2$]$^-$, 323.0 [M-H-$C_7H_8O_2$]$^-$, 295.0 [M-H-$C_8H_8O_3$]$^-$, 283.0 [M-H-$C_9H_8O_3$]$^-$,	449.0870 [M+H]$^+$	431.1 [M+H-H_2O]$^+$, 339.1 [M+H-$C_6H_6O_2$]$^+$, 327.0 [M+H-$C_7H_6O_2$]$^+$, 311.1 [M+H-$C_7H_6O_3$]$^+$, 297.0 [M+H-$C_8H_8O_3$]$^+$, 285.0 [M+H- $C_9H_8O_3$]$^+$	53.97
**43**	Mururin/vaccinin isomer[a]	220, 280, 315, 393	$C_{24}H_{16}O_9$	447.0742[M-H]$^-$	337.0 [M-H-$C_6H_6O_2$]$^-$, 325.0 [M-H-$C_7H_6O_2$]$^-$, 323.0 [M-H-$C_7H_8O_2$]$^-$, 295.0 [M-H-$C_8H_8O_3$]$^-$,	449.0862 [M+H]$^+$	339.1 [M+H-$C_6H_6O_2$]$^+$, 327.0 [M+H-$C_7H_6O_2$]$^+$, 311.1 [M+H-$C_7H_6O_3$]$^+$	55.10
**44**	Kaji-ichigoside F1 (**9a**)[c]	-	$C_{36}H_{58}O_{10}$	695.3992 [M-H+HCOOH]$^-$	649.4 [M-H]$^-$, 487.3 [M-H-162(*O*-hex)]$^-$	673.3915 [M+Na]$^+$	-	56.14
**45**	Tormentoside (**8a**)[c]	-	$C_{36}H_{58}O_{10}$	695.3991 [M-H+HCOOH]$^-$	649.4 [M-H]$^-$, 487.3 [M-H-162(*O*-hex)]$^-$	673.3909 [M+Na]$^+$	-	56.62
**46**	Triterpenoid saponin-*O*-hexoside 4[a]	-	$C_{36}H_{58}O_{10}$	695.4008 [M-H+HCOOH]$^-$	487.3 [M-H-162(*O*-hex)]$^-$	673.3911 [M+Na]$^+$	-	56.91
**47**	Triterpenoid saponin-*O*-hexoside 5[a]	-	$C_{36}H_{58}O_{10}$	695.3997 [M-H+HCOOH]$^-$	487.3 [M-H-162(*O*-hex)]$^-$	673.3913 [M+Na]$^+$	-	60.16

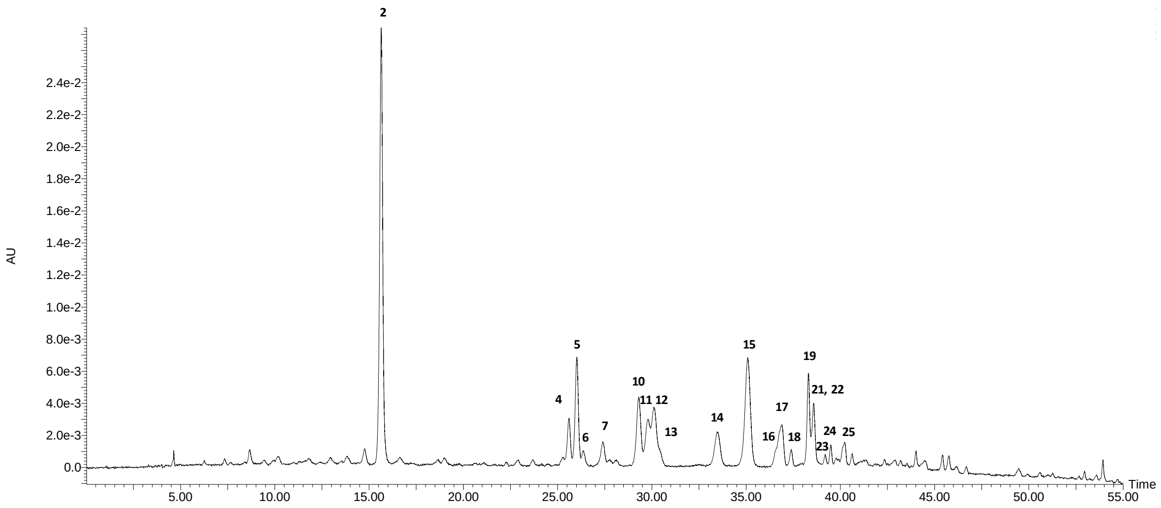

Figure S47. HPLC-UV chromatogram recorded at 340 nm of *C. obtusifolia* (O1)

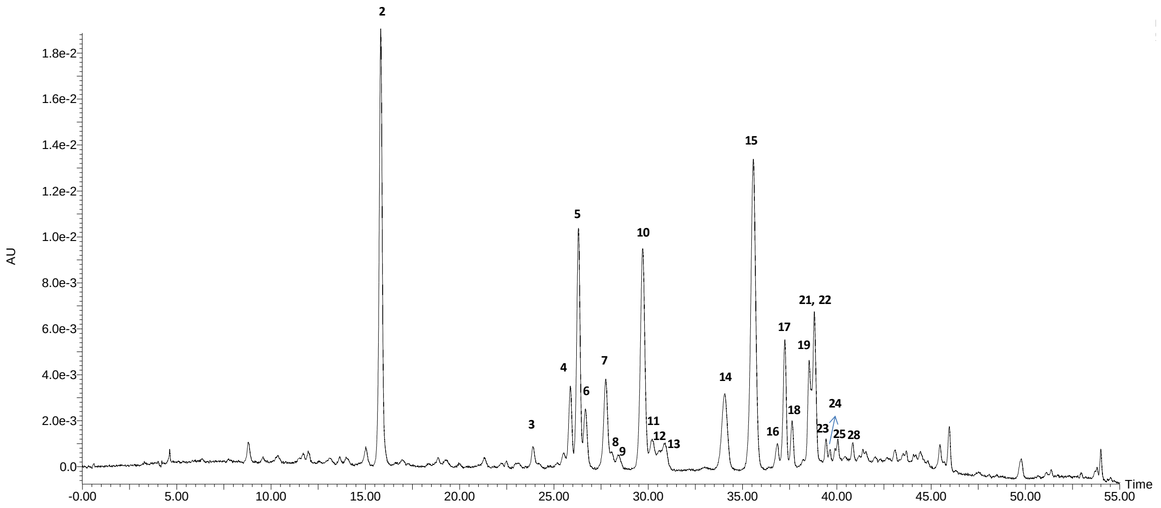

Figure S48. HPLC-UV chromatogram recorded at 340 nm of *C. obtusifolia* (O2)

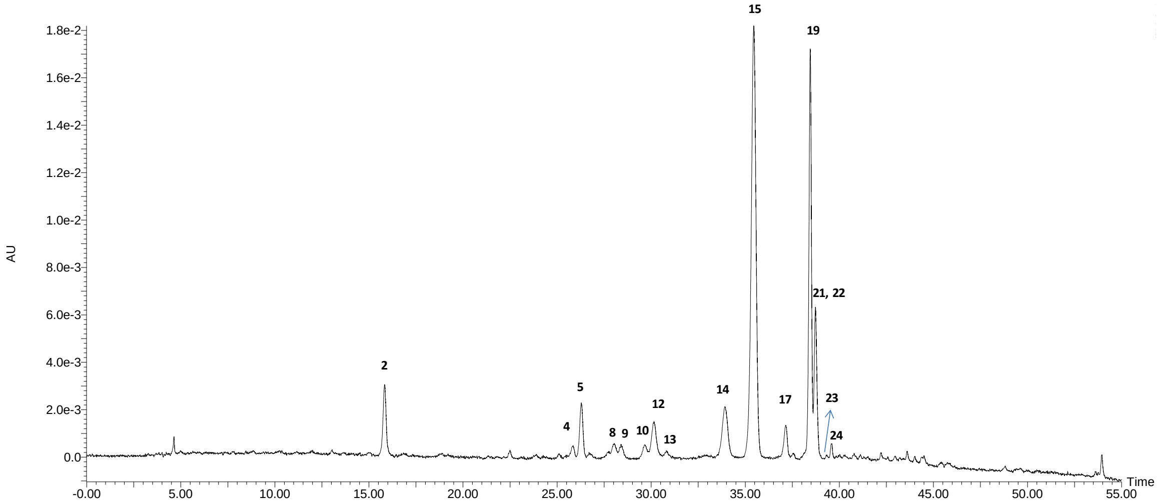

Figure S49. HPLC-UV chromatogram recorded at 340 nm of *C. obtusifolia* (O3).

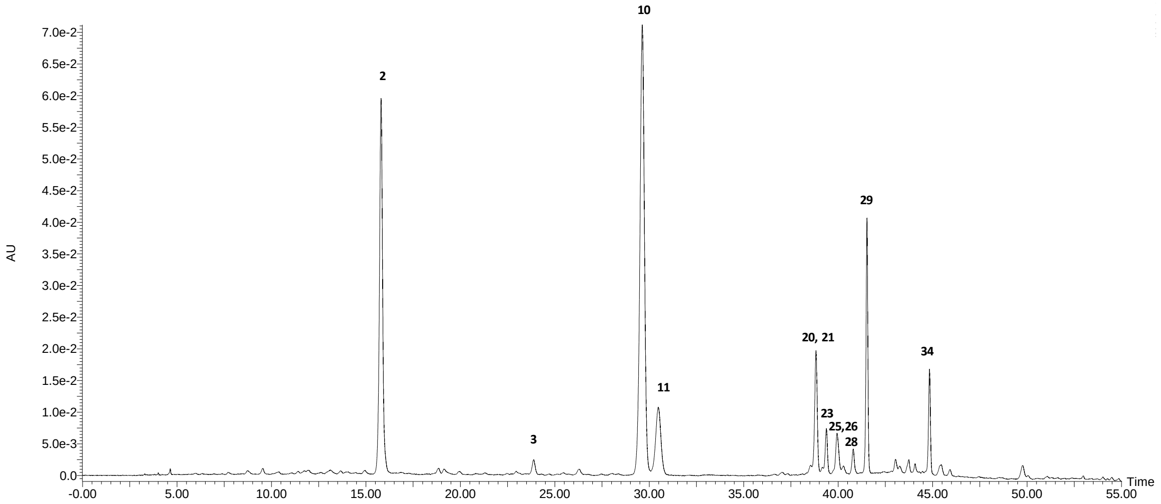

Figure S50. HPLC-UV chromatogram recorded at 340 nm of *C. obtusifolia* (O4)

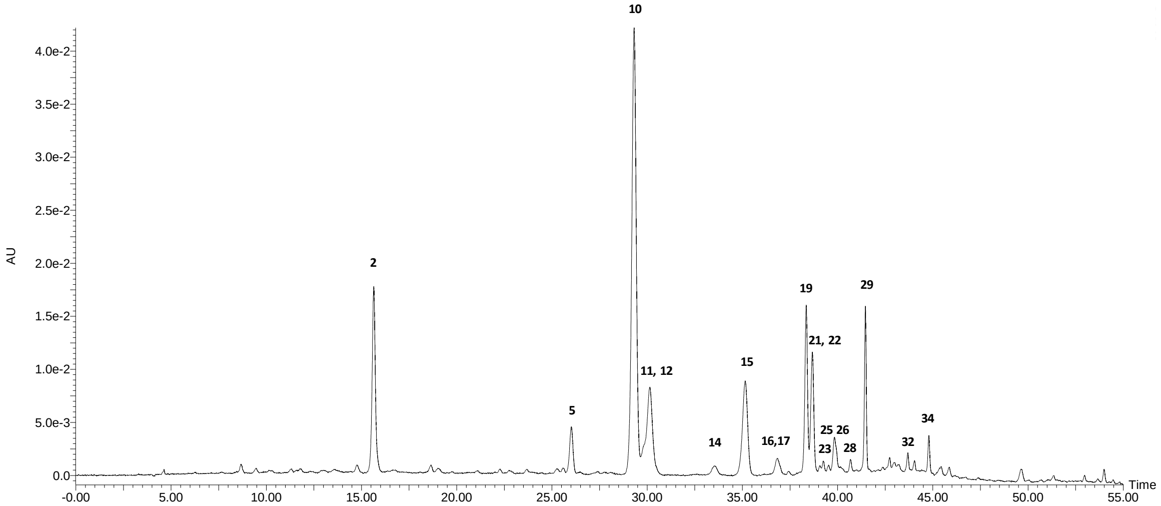

Figure S51. HPLC-UV chromatogram recorded at 340 nm of *C. obtusifolia* (O5)

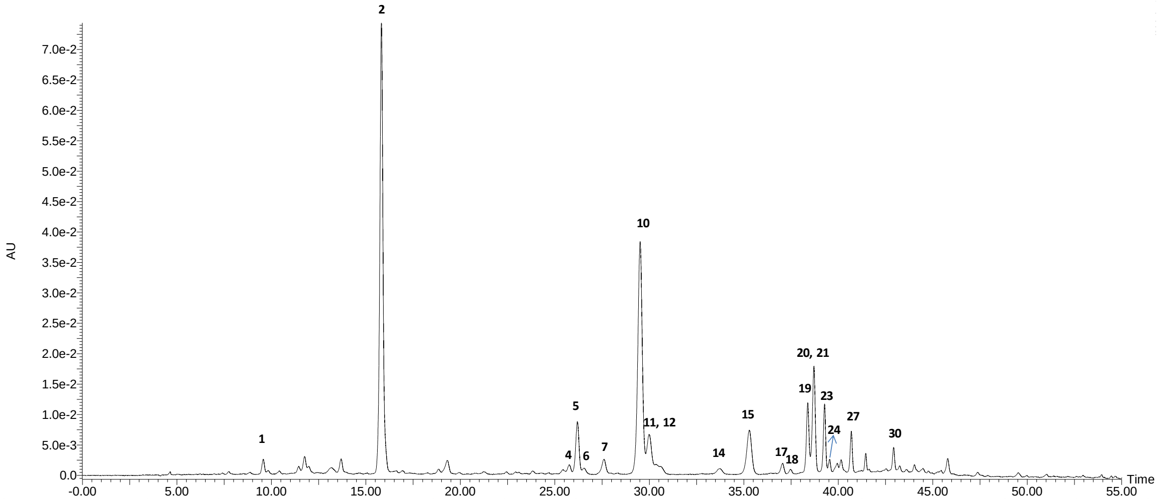

Figure S52. HPLC-UV chromatogram recorded at 340 nm of Guarumbo Tea 200 g, Nopalife (OC)

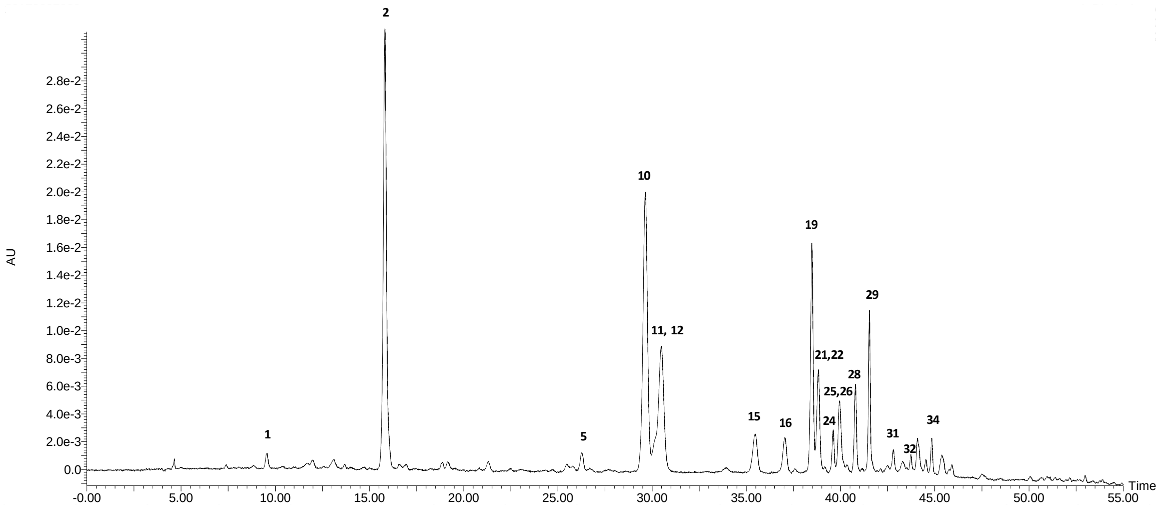

Figure S53. HPLC-UV chromatogram recorded at 340 nm of *C. peltata* (P1)

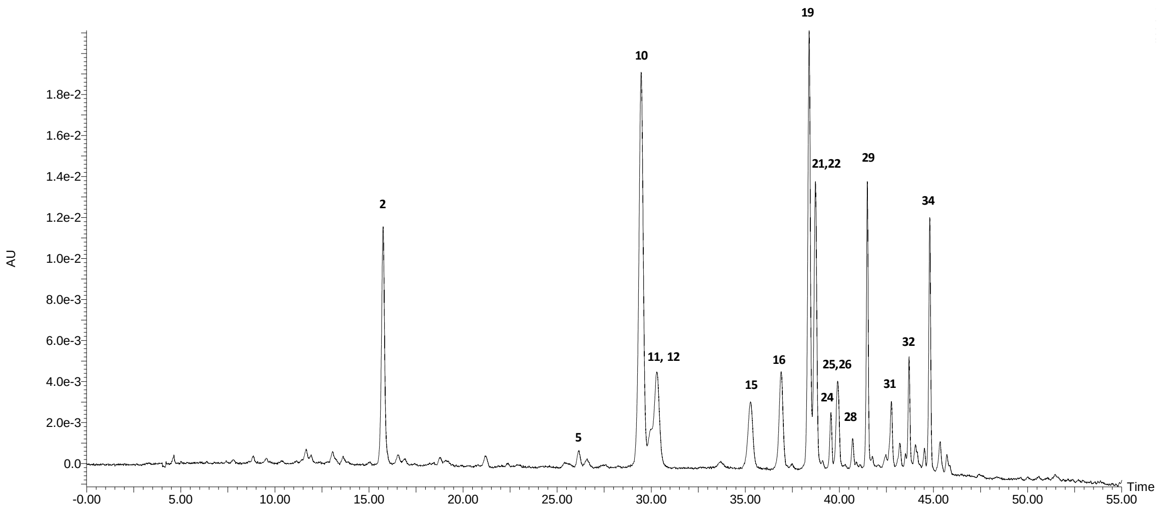

Figure S54. HPLC-UV chromatogram recorded at 340 nm of *C. peltata* (P2)

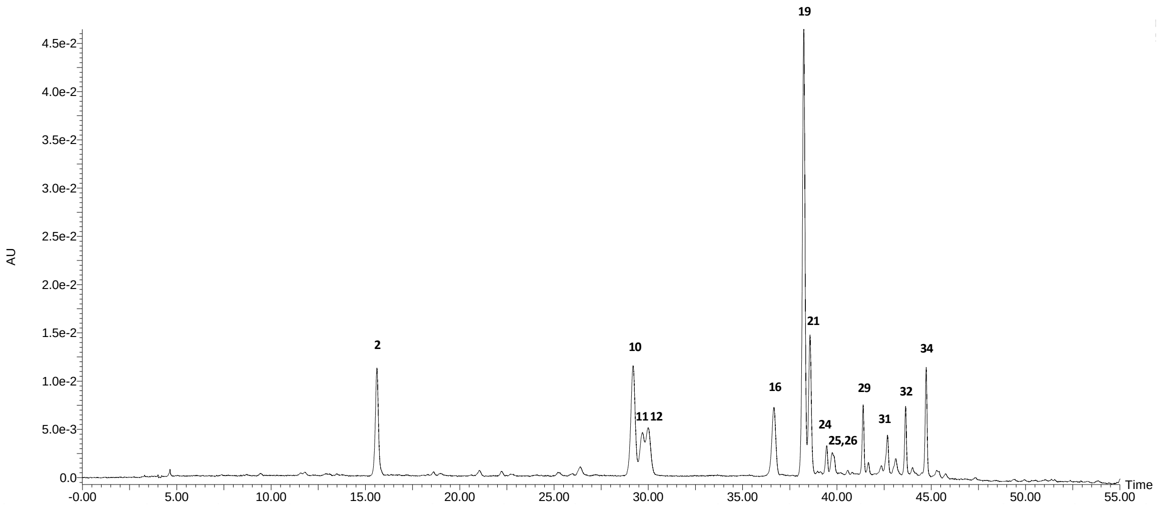

Figure S55. HPLC-UV chromatogram recorded at 340 nm of *C. peltata* (P3)

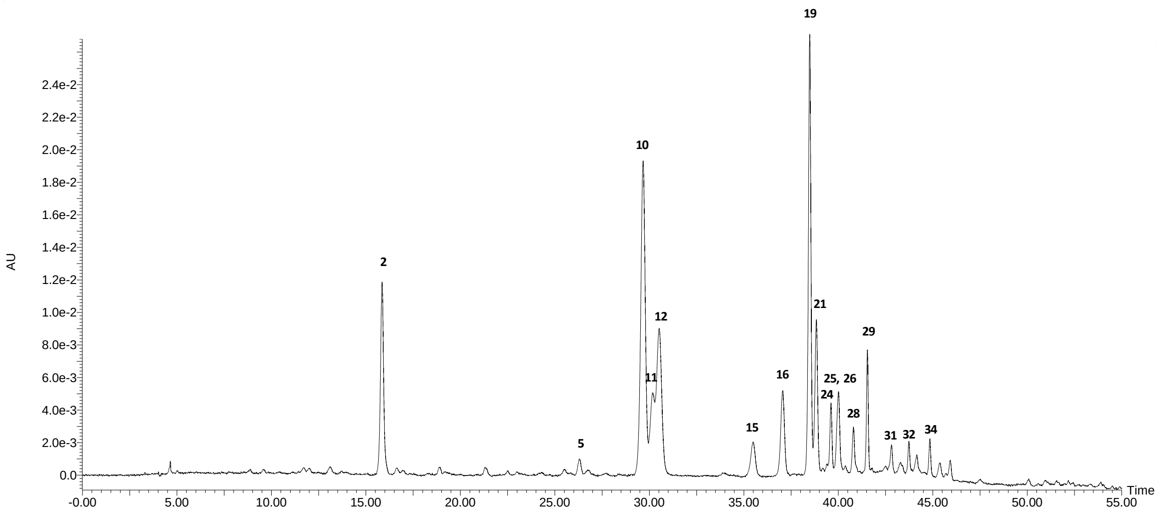

Figure S56. HPLC-UV chromatogram recorded at 340 nm of *C. peltata* (P4)

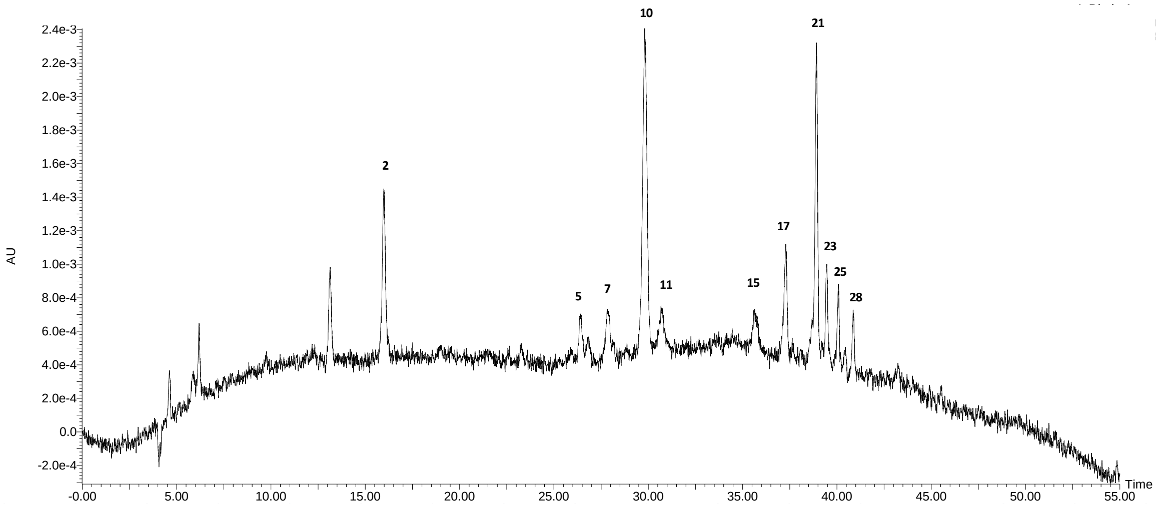

Figure S57. HPLC-UV chromatogram recorded at 340 nm of Embauba tea poweder, NaturVitae (PC)

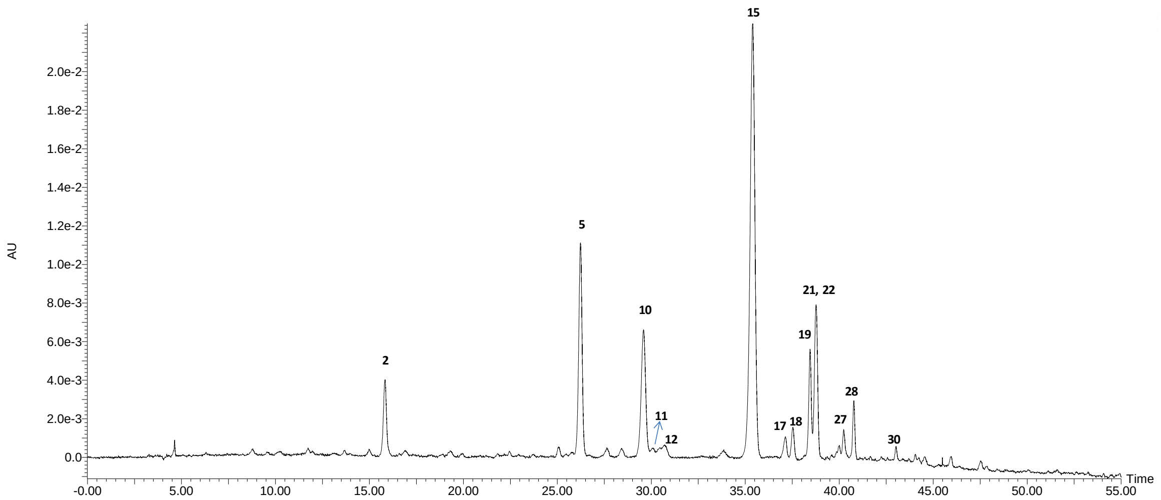

Figure S58. HPLC-UV chromatogram recorded at 340 nm of *C. insignis* (I1)

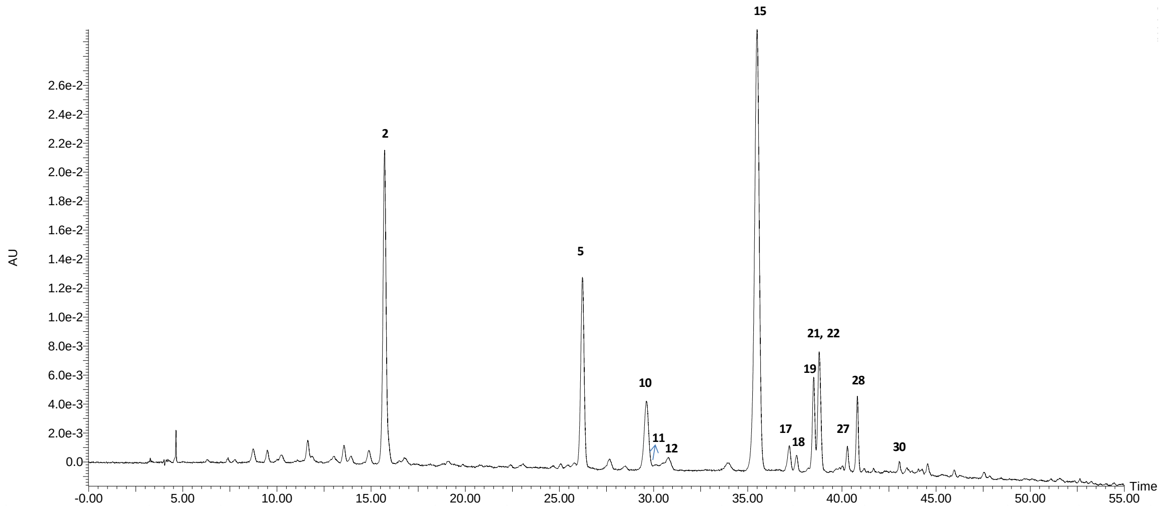

Figure S59. HPLC-UV chromatogram recorded at 340 nm of *C. insignis* (CI2)

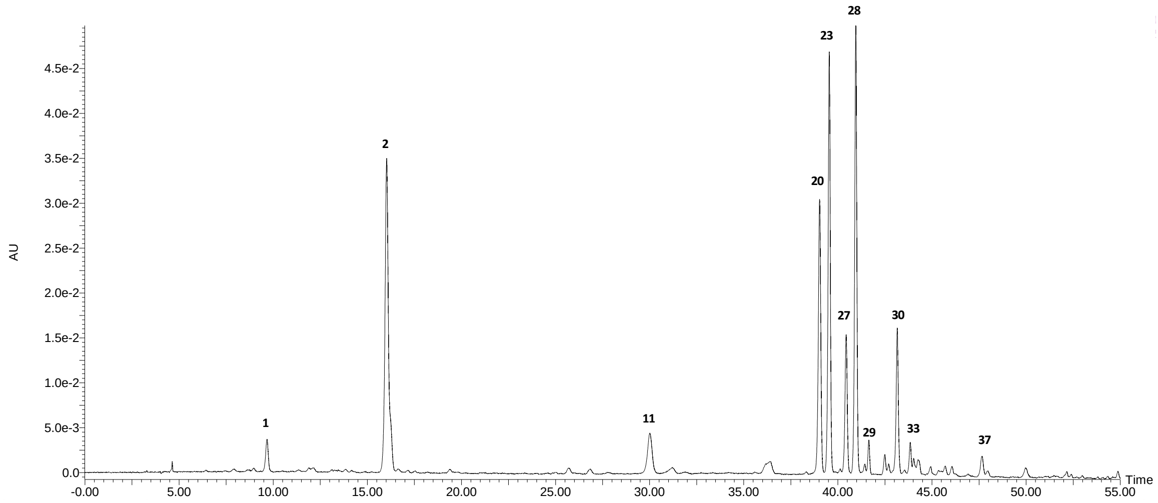

Figure S60. HPLC-UV chromatogram recorded at 340 nm of *C. hispidissima* (H1)

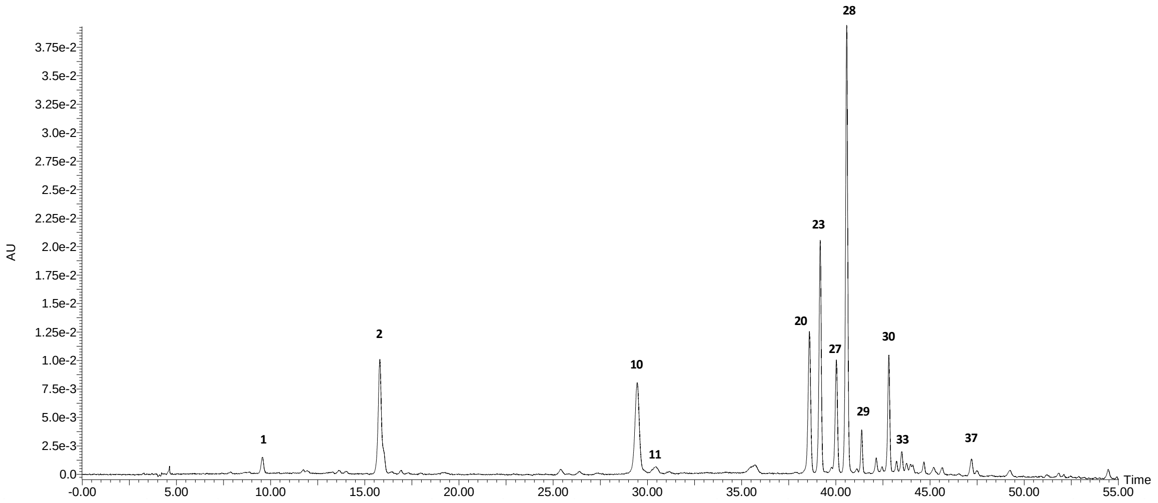

Figure S61. HPLC-UV chromatogram recorded at 340 nm of *C. hispidissima* (H2)
